# External oxidant-free electrooxidative [3 + 2] annulation between phenol and indole derivatives

**DOI:** 10.1038/s41467-017-00873-1

**Published:** 2017-10-03

**Authors:** Kun Liu, Shan Tang, Pengfei Huang, Aiwen Lei

**Affiliations:** 1 College of Chemistry and Molecular Sciences, the Institute for Advanced Studies (IAS), Wuhan University, Wuhan, 430072 Hubei China; 2 State Key Laboratory for Oxo Synthesis and Selective Oxidation, Lanzhou Institute of Chemical Physics, Chinese Academy of Sciences, Lanzhou, 730000 China

## Abstract

Intermolecular [3 + 2] annulation is one of the most straightforward approaches to construct five membered heterocycles. However, it generally requires the use of functionalized substrates. An ideal reaction approach is to achieve dehydrogenative [3 + 2] annulation under oxidant-free conditions. Here we show an electrooxidative [3 + 2] annulation between phenols and *N-*acetylindoles under undivided electrolytic conditions. Neither external chemical oxidants nor metal catalysts are required to facilitate the dehydrogenation processes. This reaction protocol provides an environmentally friendly way for the selective synthesis of benzofuroindolines. Various *N*-acetylindoles bearing different C-3 and C-2 substituents are suitable in this electrochemical transformation, furnishing corresponding benzofuroindolines in up to 99% yield.

## Abstract (web summary)

Electrochemical oxidation provides a green alternative to the use of hazardous chemical oxidants and forcing conditions. Here, the authors show the electrocatalytic cross-coupling of phenols and indoles to generate biologically relevant benzofuroindolines in high yields.

## Introduction

Five-membered heterocycles are highly important structural motifs, which widely exist in natural products, pharmaceuticals, and fundamental materials^1–3^. Intermolecular [3 + 2] annulation are mostly employed approaches to construct five-membered heterocycles. One typical method is 1,3-dipole cycloaddition, which generally requires the use of functionalized substrates such as nitrones, azomethine ylides, azomethine imine, and azides^4–7^. To fulfill the demand by green and sustainable chemistry, oxidative [3 + 2] annulation has been gradually developed to pursuit the synthesis of five-membered heterocycles from readily available starting materials^8–12^. However, external chemical oxidants are often required to facilitate the dehydrogenation processes, which lead to the decreased atom economy of the overall transformation. In addition, using strong chemical oxidants can also cause over-oxidation issues, which makes it difficult to achieve high reaction efficiency. Developing dehydrogenative [3 + 2] annulation under oxidant-free conditions may provide solutions to these problems. Anodic oxidation represents an effective alternative to the oxidation by external chemical oxidants^13–19^. Over the past decade, increasing efforts have been made to achieving dehydrogenative cross-coupling under electrochemical conditions^20–35^.

**Fig. 1:**
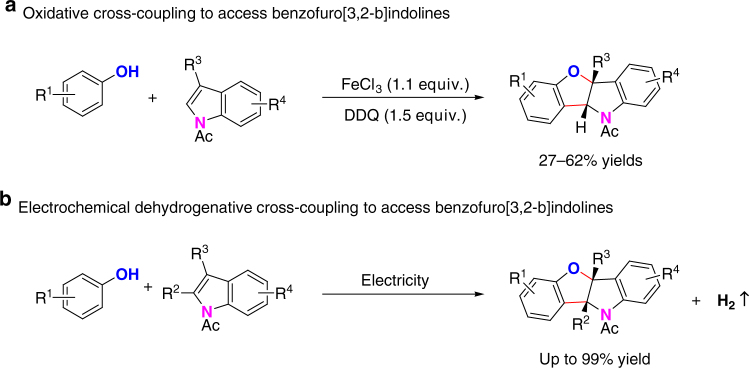
Synthesis of benzofuro[3,2-b]indolines. **a** Synthesis of benzofuro[3,2-b]indolines by Vincent and co-workers. **b** Electrochemical synthesis of benzofuro[3,2-b]indolines under external oxidant-free conditions

Benzofuroindoline motifs exist in some important bioactive natural products such as diazonamides^36, 37^, azonazines^38^, and phalarine^39^. The direct oxidative [3 + 2] annulation between phenols and indoles provides a straightforward and atom economic way for the synthesis of benzofuroindolines, which could potentially lead to the formation of two regioisomers. In analogous to the natural bias, the oxidative [3 + 2] annulation between phenols and indoles usually gives benzofuro[2,3-b]indolines. Over the past decade, different reaction protocols for the synthesis of benzofuro[2,3-b]indoline moiety have been developed by Harran^37, 40^, Danishefsky^41^, Vincent^42, 43^, and others^44–46^. In contrast, attempts on phenol–indoles coupling for the synthesis of benzofuro[3,2-b]indolines have rarely succeeded^47^. In 2014, Vincent and co-workers reported an oxidative [3 + 2] annulation between phenols and 3-substituted *N*-acetylindoles for the synthesis of benzofuro[3,2]indolines using excess amount of FeCl_3_ and 2,3-dicyano-5,6-dichlorobenzoquinone, and the reaction yields ranged from 27 to 62% (Fig. 1a)^48^. It is highly desirable to develop more efficient phenol–indole [3 + 2] annulation for the synthesis of benzofuro[3,2-b]indolines.

Here we present an efficient electrooxidative [3 + 2] annulation between phenols and *N*-acetylindoles in a simple undivided cell. It enabled the selective synthesis of benzofuro[3,2]indolines under external oxidant- and catalyst-free conditions. Various *N-*acetylindoles bearing different C-3 substituents are suitable in this electrochemical transformation and can afford corresponding benzofuro[3,2]indolines in up to 99% yield.

## Results

### Investigation of reaction conditions

We anticipated that electrochemical anodic oxidation might provide a way to achieve external oxidant-free dehydrogenative [3 + 2] annulation (Fig. 1b). *p*-Methoxylphenol (**1a**) and 3-methyl-*N*-acetylindole (**2a**) were chosen as model substrates to test the reaction conditions. Utilizing *n*Bu_4_NBF_4_ as the electrolyte and 1,1,1,3,3,3-hexafluoroisopropyl alcohol (HFIP)/CH_2_Cl_2_ as co-solvents, benzofuro[3,2-b]indoline **3a** could be obtained in a quantitative yield under 10 mA constant current for 1.8 h in an undivided cell (Table 1, entry 1). Decreased reaction yields were obtained when increasing or decreasing the operating current (Table 1, entries 2 and 3). In addition, solvent effect was also investigated in this transformation. CH_2_Cl_2_ was not indispensable for this electrochemical dehydrogenative cross-coupling reaction. A good reaction efficiency could still be achieved when HFIP was used as the sole solvent (Table 1, entry 4). However, HFIP was found to be crucial since no desired product could be obtained when dichloromethane was used solely or HFIP was replaced by methanol (Table 1, entries 5 and 6). As for the electrolyte used, the counter anion had slight effect on the reaction efficiency. *n*Bu_4_NClO_4_ and *n*Bu_4_NPF_6_ were also suitable in this transformation (Table 1, entries 7 and 8). The effect of the electrode material was also explored. Both replacing graphite rod anode by platinum plate anode and replacing platinum plate cathode by graphite rod cathode led to lower reaction yields (Table 1, entries 9 and 10). However, platinum plate cathode was possible to be replaced by cheap nickel plate cathode for this dehydrogenative [3 + 2] annulation reaction (Table 1, entry 11). Importantly, the reaction could be conducted under atmospheric conditions with a high reaction efficiency (Table 1, entry 12). Obviously, no reaction took place without electric current under air atmosphere (Table 1, entry 13).

**Table 1:** Effects of reaction parameters^a^


Entry	Variation from the standard conditions	Yield
1	None	99%
2	5 mA instead of 10 mA, 3.6 h	85%
3	20 mA instead of 10 mA, 0.9 h	86%
4	Without CH_2_Cl_2_	78%
5	Without HFIP	ND
6	MeOH instead of HFIP	ND
7	*n*Bu_4_NClO_4_ instead of *n*Bu_4_NBF_4_	90%
8	*n*Bu_4_NPF_6_ instead of *n*Bu_4_NBF_4_	85%
9	Platinum plate anode instead of graphite rod anode	78%
10	Graphite rod cathode instead of platinum plate cathode	71%
11	Nickel plate cathode instead of platinum plate cathode	93%
12	Under air	94%
13	No electric current, under air	NR

ND not detected, NR no reaction.

^a^Standard conditions: graphite rod anode (*ϕ* 6 mm), platinum plate cathode (15 mm × 15 mm × 0.3 mm), constant current = 10 mA, **1a** (0.20 mmol), **2a** (0.30 mmol), *n*Bu_4_NBF_4_ (0.20 mmol), solvent (10 ml), room temperature, N_2_, 1.8 h (3.4 F). Yields are determined by GC analysis with biphenyl as the internal standard

### Substrate scope

**Fig. 2:**
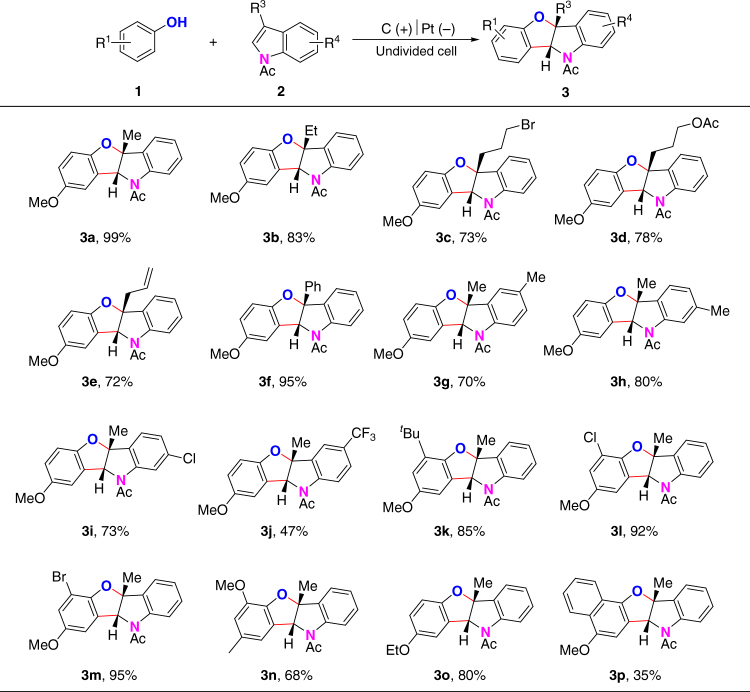
Synthesis of benzofuro[3,2-b]indolines from 3-substituted *N*-acetylindoles. Reaction conditions: graphite rod anode (*ϕ* 6 mm), platinum plate cathode (15 mm × 15 mm × 0.3 mm), constant current = 10 mA (*J* ≈ 16.7 mA/cm^2^), **1** (0.20 mmol), **2** (0.30 mmol), *n*Bu_4_NBF_4_ (0.20 mmol), HFIP/CH_2_Cl_2_ (6.0 ml/4.0 ml), room temperature, N_2_, 1.8 h (3.4 F). Isolated yields are shown

To demonstrate the applicability of this transformation, we first turned to explore the substrate scope for the synthesis of benzofuro[3,2-b]indolines (Fig. 2). The reaction between *p*-methoxylphenol and *N*-acyl indoles bearing different C-3 substituents such as simple alkyl, functionalized alkyl, allyl and phenyl groups were all suitable in this dehydrogenative [3 + 2] annulation reaction, affording corresponding benzofuro[3,2-b]indolines in good to excellent yields (**3a**–**3f**). *N*-acetylindoles bearing electron neutral substituents such as methyl group and chloride at C-5 or C-6 position also showed good reactivity in the synthesis of benzofuro[3,2-b]indolines (**3g**–**3i**). Strong electron-withdrawing trifluoromethyl group at the C-5 position led to a decreased reaction yield (**3j**). Strong electron-donating groups at C-5 or C-6 position were not tolerated under the electrochemical conditions. In the next step, efforts were also taken to test the scope of phenols. Alkoxy substituents at the *ortho* or *para* position of phenols were essential for achieving good reaction efficiency. Electron neutral phenols showed decreased reactivity under the standard conditions. *p*-Methoxyphenol bearing a bulky *tert*-butyl group at the C-2 position was still able to synthesize benzofuro[3,2-b]indoline in a good yield (**3k**). *Ortho*-halide substituents including Cl and Br were well tolerated in the synthesis of benzofuro[3,2-b]indolines (**3l** and **3m**). Phenols bearing methoxyl group at the *ortho* position or bearing ethoxyl group at the *para* position both afforded the desired products in good yields (**3n** and **3o**). Electron-rich 4-methoxynaphtol could also afford the desired benzofuroindoline **3p** in 35% yield.

**Fig. 3:**
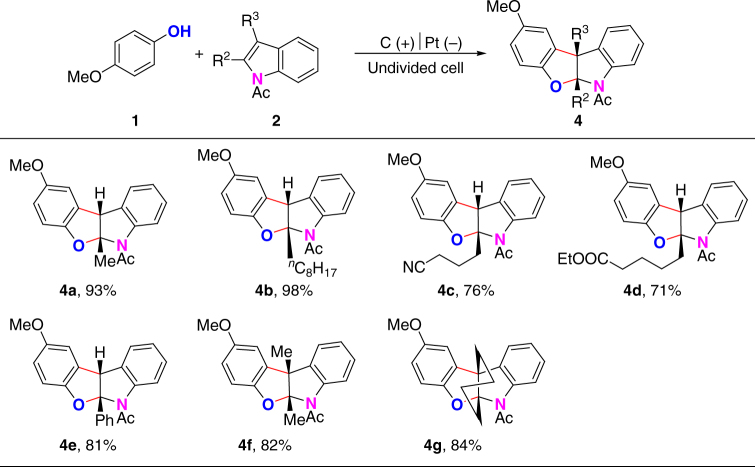
Synthesis of benzofuro[2,3-b]indolines from 2-substituted *N*-acetylindoles. Reaction conditions: graphite rod anode (*ϕ* 6 mm), platinum plate cathode (15 mm × 15 mm × 0.3 mm), constant current = 10 mA, **1a** (0.20 mmol), **2a** (0.30 mmol), *n*Bu_4_NBF_4_ (0.20 mmol), HFIP/CH_2_Cl_2_ (6.0 ml/4.0 ml), room temperature, N_2_, 1.8 h (3.4 F). Isolated yields are shown

Besides 3-substituted *N-*acetylindoles, 2-substituted *N*-acetylindoles were also applied as substrates in this transformation (Fig. 3). Under the standard conditions, the reaction between *p*-methoxylphenol and 2-methyl-*N*-acetylindole selectively furnished benzofuro[2,3-b]indoline **4a**. Similarly, the reactions of other 2-substituted *N-*acetylindoles with *p*-methoxylphenol were only able to give benzofuro[2,3-b]indolines (**4b**–**4e**). Moreover, 2,3-disubstituted *N*-acetylindoles were also able to participate in the electrooxidative [3 + 2] annulation reaction for the synthesis of benzofuro[2,3-b]indolines (**4f**–**4g**).

**Fig. 4:**
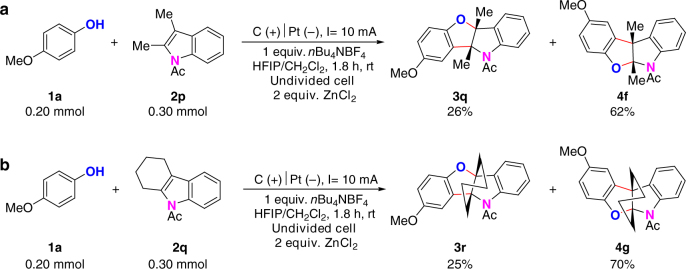
Synthesis of benzofuro[3,2-b]indolines from 2,3-disubstituted *N*-acetylindoles. **a** Reaction with 2,3-dimethyl-*N-*acetylindole by adding ZnCl_2_. **b** Reaction with *N*-acetyl tetrahydrocarbazole by adding ZnCl_2_

It has been noted that coordination of Lewis acid with *N*-acetyl indoles can change the classical polarity of the C2 and C3-positions on indoles^49^. In order to access benzofuro[3,2-b]indolines, we have tried to add Lewis acids into the electrooxidative [3 + 2] annulation reaction with 2-substituted *N*-acetylindoles and 2,3-disubstituted *N*-acetylindoles. By adding 2 equiv. of ZnCl_2_, 26% of benzofuro[3,2-b]indoline **3q** could be obtained from the reaction between 2,3-dimethyl-*N-*acetylindole and *p*-methoxylphenol (Fig. 4a). Similarly, the reaction between 2,3-dimethyl-*N-*acetylindole and *p*-methoxylphenol furnished corresponding benzofuro[3,2-b]indoline in 25% yield by adding 2 equiv. of ZnCl_2_ (Fig. 4b). However, the reaction selectivity with 2-substituted *N*-acetylindoles could not be tuned to benzofuro[3,2-b]indolines even by adding Lewis acids.

**Fig. 5:**
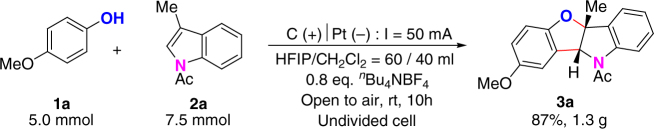
Electrochemical gram scale reaction. Gram scale synthesis of benzofuro[3,2-b]indoline **3a**

The scalability of this electrooxidative [3 + 2] annulation was then evaluated by performing a 5.0 mmol scale reaction. Under atmospheric conditions, the gram scale reaction between **1a** and **2a** afforded the corresponding benzofuro[3,2-b]indoline **3a** in a high reaction efficiency with a 87% yield (Figs. 5 and 3a, 1.3 g). This result demonstrated the great potential of this electrooxidative [3 + 2] annulation in future application.

## Discussion

**Fig. 6:**
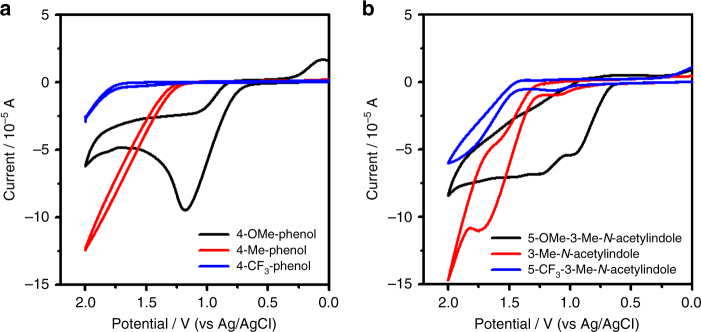
Cyclic voltammograms in HFIP/CH_2_Cl_2_ with 0.20 M *n*Bu_4_NBF_4_. **a** Cyclic voltammograms of different phenols. **b** Cyclic voltammograms of different *N*-acetylindoles

To get some insight into the electron transfer processes, cyclic voltammetry experiments of phenols and *N*-acetylindoles were conducted. As shown in Fig. 6a, an obvious oxidation peak of *p*-methoxylphenol could be observed at 1.16 V while no obvious oxidation peak of *p*-methylphenol and *p*-trifluoromethylphenol could be observed in their cyclic voltammograms. Cyclic voltammograms of *N*-acetylindoles with different electron density were also presented. Oxidation peaks of 3-methyl-5-methoxyl-*N*-acetylindole were observed above 0.98 V while oxidation peaks of 3-methyl-*N*-acetylindole and 3-methyl-5-trifluoromethyl-*N*-acetylindole were observed at 1.10 V and 1.15 V, respectively (Fig. 6b). Interestingly, the oxidation potential of *p*-methoxylphenol was quite close to 3-methyl-*N*-acetylindole and 3-methyl-5-trifluoromethyl-*N*-acetylindole. Therefore, the oxidation of both substrates was possible under the electrolytic conditions.

**Fig. 7:**
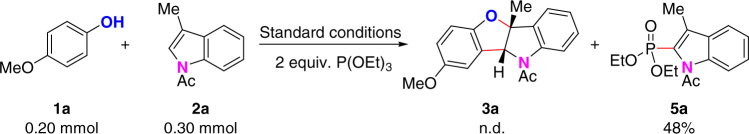
Radical trapping experiment. Radical trapping experiment by P(OEt)_3_

Since both of the substrates were possible to be oxidized by the anode, a radical trapping experiment by triethyl phosphite was conducted to explore the existence of radical intermediates (Fig. 7). No desired benzofuro[3,2-b]indoline could be observed. Instead, an indole phosphorylation product **5a** could be obtained in 48% yield. These results indicated that the reaction might go through a radical mechanism and indole cation radical intermediate was likely to be generated during electrolysis. It has been reported that radical cations of aromatic compounds (ArH^•+^) generated in HFIP are extremely persistent^50–52^. According to the persistent radical effect, the radical coupling between a persistent radical and a transient radical would lead to selective bond formation^53^. The indole cation radical could be considered as a persistent radical while phenoxy radical was a transient radical. Thus, the coupling of the indole cation radical and the phenoxy radical was possible to be involved for this transformation.

**Fig. 8:**
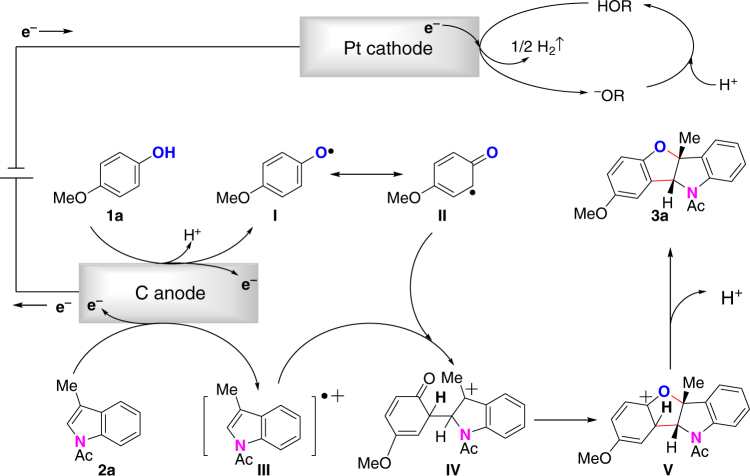
Proposed reaction mechanism. Tentative reaction mechanism involves anode oxidation of phenol to generate phenol radical and *N-*acetylindole to generate indole cation radical, cross-coupling of phenoxy radical with indole cation radical, intramolecular cyclization and deprotonation to furnish the final product

Based on the experimental results and previous reports^10, 48^, a plausible reaction mechanism between **1a** and **2a** is presented in Fig. 8. A single-electron-transfer oxidation of *p*-methoxylphenol by anodic oxidation generates a phenol oxygen radical **I**. The oxygen raidcal **I** can be isomerized to carbon radical **II** ^22^. At the same time, *N*-acetylindole can also be oxidized by the anode to afford cation radical intermediate **III**. Direct cross-coupling of carbon radical **II** with cation radical intermediate **III** will form cation intermediate **IV**. Following intramolecular cyclization and deprotonation of **IV** will generate benzofuro[3,2-b]indoline **3a**. Meanwhile, HFIP is reduced at the Pt cathode to afford hydrogen gas.

In summary, we have developed a green and efficient electrooxidative [3 + 2] annulation between phenols and *N-*acetylindoles. This reaction protocol avoids the use of external chemical oxidants and H_2_ is the only byproduct. Under undivided electrolytic conditions, a series of benzofuro[3,2-b]indolines can be obtained in good to excellent yields. Significantly, this reaction can be conducted in gram scale under atmospheric conditions. Mechanistically, both phenol and *N-*acetylindole are considered to be oxidized by anode to generate radical intermediates during the reaction. In this reaction case, electrochemical external oxidant-free dehydrogenative cross-coupling demonstrated higher reaction efficiency than traditional oxidative cross-coupling protocol, which may inspire people to use electrochemical methods in more oxidative cross-coupling reactions.

## Methods

### Representative procedure for the synthesis of benzofuro[3,2-b]indoline (**3a**)

In an oven-dried undivided three-necked bottle (25 ml) equipped with a stir bar, *p*-methoxylphenol (24.8 mg, 0.20 mmol), 3-methyl-*N*-acetylindole (51.9 mg, 0.30 mmol), *n*Bu_4_NBF_4_ (65.8 mg, 0.20 mmol), and HFIP/CH_2_Cl_2_ (6.0 ml/4.0 ml) were combined and added. The bottle was equipped with graphite rod (*ϕ* 6 mm, about 10 mm immersion depth in solution) as the anode and platinum plate (15 mm × 15 mm × 0.3 mm) as the cathode and then charged with nitrogen. The reaction mixture was stirred and electrolyzed at a constant current of 10 mA (*j* ≈ 16 mA/cm^2^) under room temperature for 1.8 h. When the reaction finished, the reaction mixture was washed with water and extracted with CH_2_Cl_2_ (10 ml × 3). The organic layers were combined, dried over Na_2_SO_4_, and concentrated. The pure product was obtained by flash column chromatography on silica gel (hexane: ethyl acetate = 10:1). Yellow oil was obtained in 99% isolated yield. Since the acetyl group could form intramolecular hydrogen bonds with the hydrogens adjacent to nitrogen atom, the spectra demonstrate a mixture of rotamers (74:26). ^1^H NMR (400 MHz, CDCl_3_) *δ* 8.16 (d, *J* = 7.6 Hz, 0.3H), 7.55–7.48 (d, *J* = 7.6 Hz, 0.7H), 7.44 (d, *J* = 6.8 Hz, 0.3H) 7.30 (t, *J* = 7.6 Hz, 1.7H), 7.12 (t, *J* = 7.3 Hz, 1.7H), 6.93 (s, 0.3H), 6.83–6.57 (m, 2H), 5.96 (s, 0.7H), 5.61 (s, 0.3H), 3.73 (s, 3H), 2.57(s, 0.8H), 2.49 (s, 2.2H), 1.82 (s, 3H). ^13^C NMR (101 MHz, CDCl_3_) *δ* 168.71, 167.98, 154.36, 152.97, 152.84, 141.09, 140.35, 135.01, 133.68, 129.93, 126.94, 126.02, 125.02, 124.85, 124.01, 123.44, 118.08, 116.89, 116.11, 114.47, 112.45, 111.02, 110.19, 92.79, 90.60, 72.38, 71.83, 55.99, 25.04, 24.80, 24.27. For ^1^H NMR, ^13^C NMR, ^19^F NMR and ^31^P NMR (if applicable) spectra of compounds **3a**–**3r**, **4a**–**4g**, **5a**, see Supplementary Figs. 1–54. For the general information of the analytical methods and procedure for cyclic voltammetry please see Supplementary Methods.

### Procedure for gram scale synthesis of **3a**

In an oven-dried conical flask (100 ml) equipped with a stir bar, 4-methoxyphenol (0.62 g, 5.0 mmol), 3-methyl-*N*-acetylindole (1.3 g, 7.5 mmol), *n*Bu_4_NBF_4_ (1.3 g, 4.0 mmol), and HFIP/CH_2_Cl_2_ (60 ml/40 ml) were combined and added. The bottle was equipped with graphite rod (*ϕ* 6 mm, about 10 mm immersion depth in solution) as the anode and platinum plate (15 mm × 15 mm × 0.3 mm) as the cathode. The reaction mixture was stirred and electrolyzed at a constant current of 50 mA (*j*_anode_ ≈ 83 mA/cm^2^) under air atmosphere at room temperature for 10 h (3.7 F). When the reaction finished, the reaction mixture was washed with water and extracted with CH_2_Cl_2_ (100 ml × 3). The organic layers were combined, dried over Na_2_SO_4_, and concentrated. The pure product was obtained by flash column chromatography on silica gel (hexane: ethyl acetate = 10:1). Yellow oil was obtained in 87% isolated yield (1.3 g). For the experimental setup diagram for the gram scale reaction see Supplementary Fig. 55.

### Data availability

The authors declare that the data supporting the findings of this study are available within the article and its Supplementary Information files.

## Electronic supplementary material

Supplementary Information
