# Supplementary material for: External oxidant-free electrooxidative [3 + 2] annulation between phenol and indole derivatives
Source: Nat Commun. 2017 Oct 3;8:775. doi: 10.1038/s41467-017-00873-1 (PMC5626759; doi:10.1038/s41467-017-00873-1)
Supplement: Supplementary file 1 — Supplementary Information [file 41467_2017_873_MOESM1_ESM.pdf]

Supplementary Figures

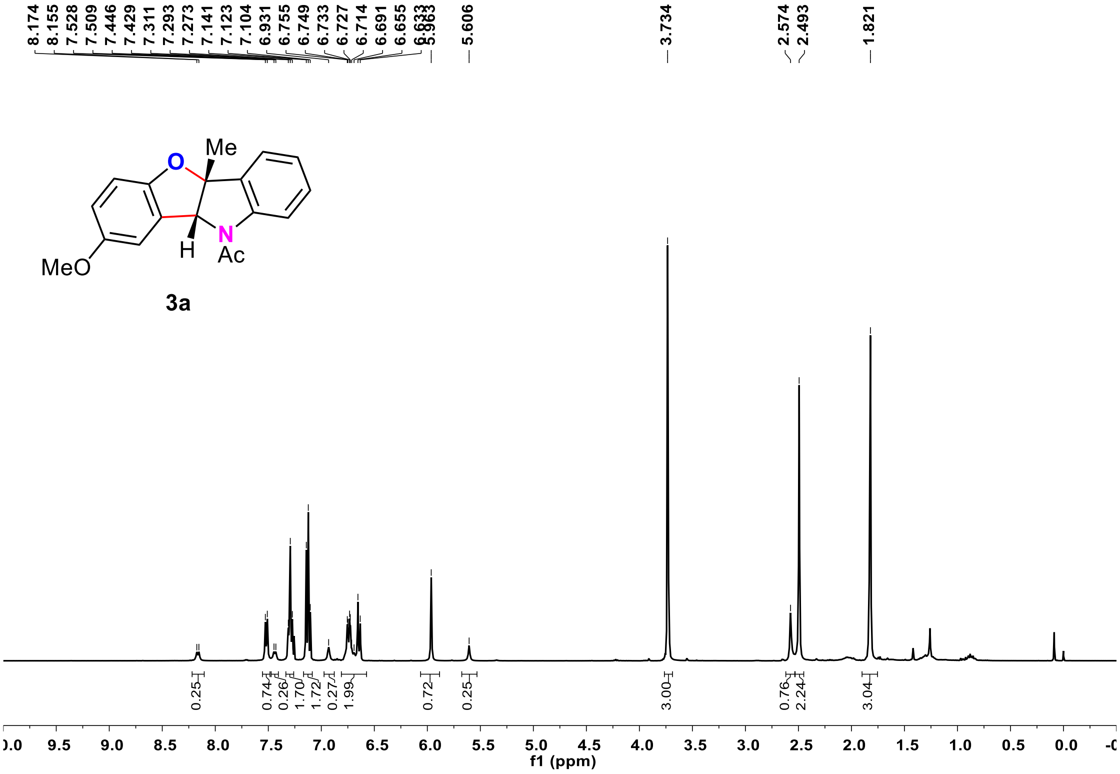

Supplementary Figure 1. ^{1}H NMR (400 MHz, CDCl$_3$) spectrum of 3a

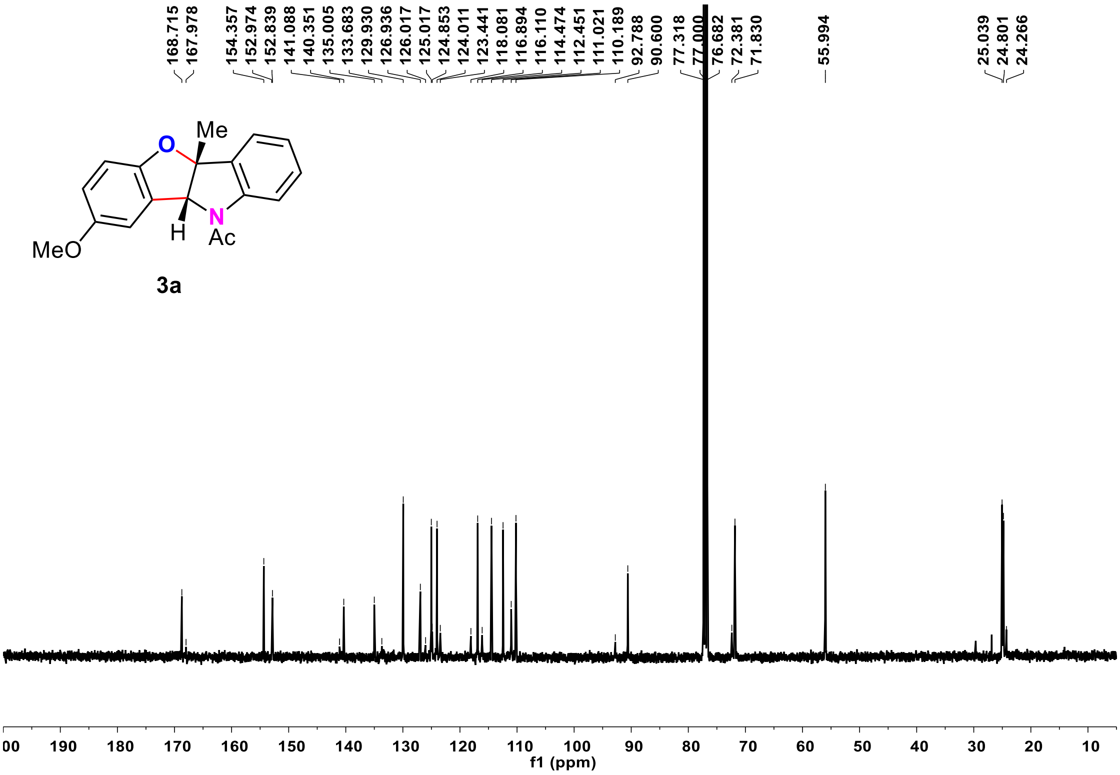

Supplementary Figure 2. ^{13}C NMR (101 MHz, CDCl$_3$) spectrum of 3a

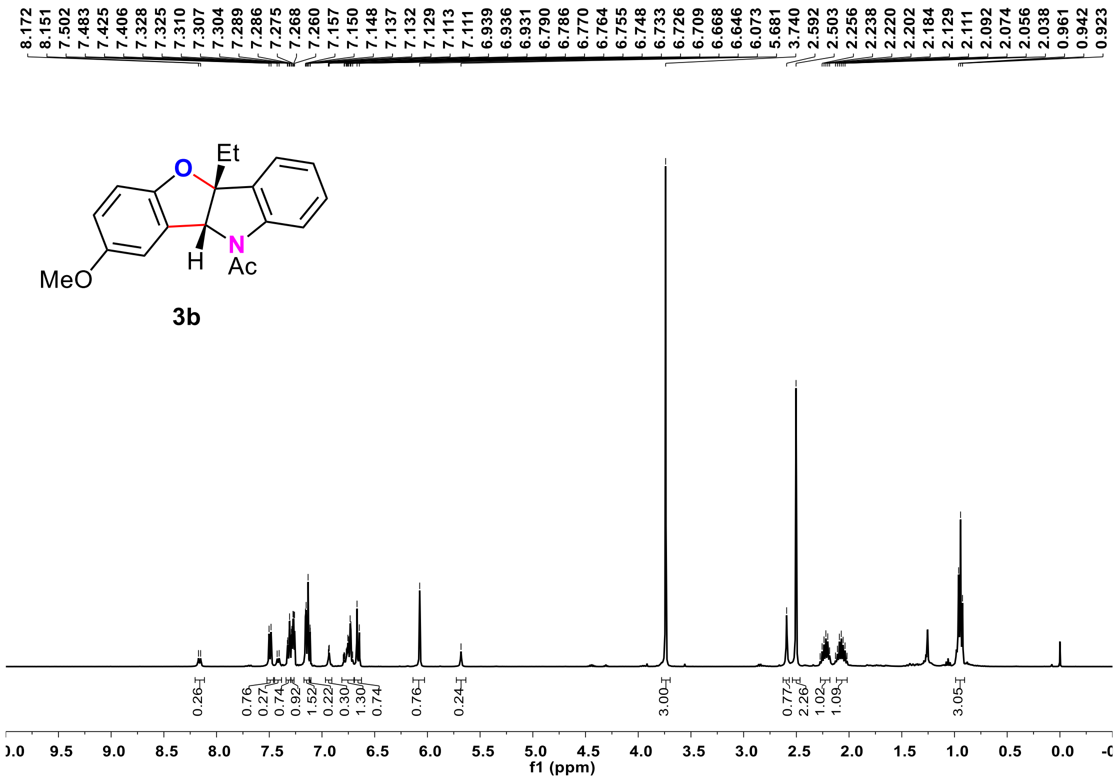

Supplementary Figure 3. ^{1}H NMR (400 MHz, $CDCl_3$) spectrum of 3b

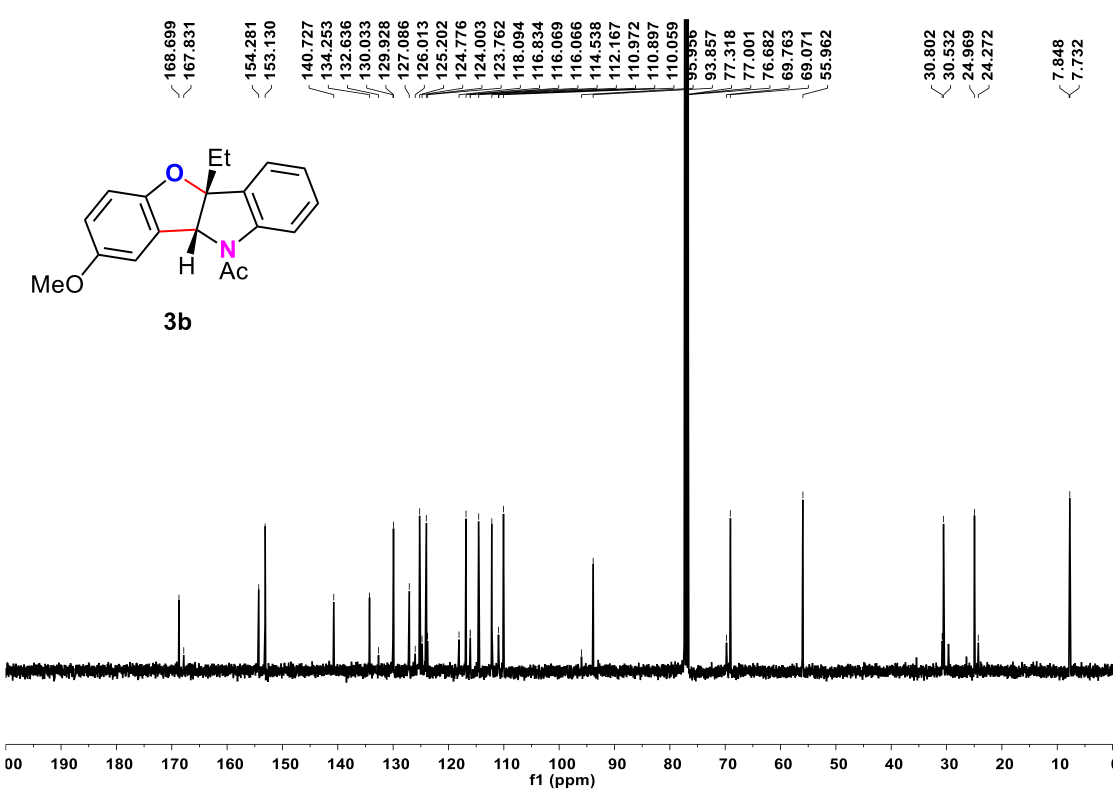

Supplementary Figure 4. ^{13}C NMR (101 MHz, $CDCl_3$) spectrum of 3b

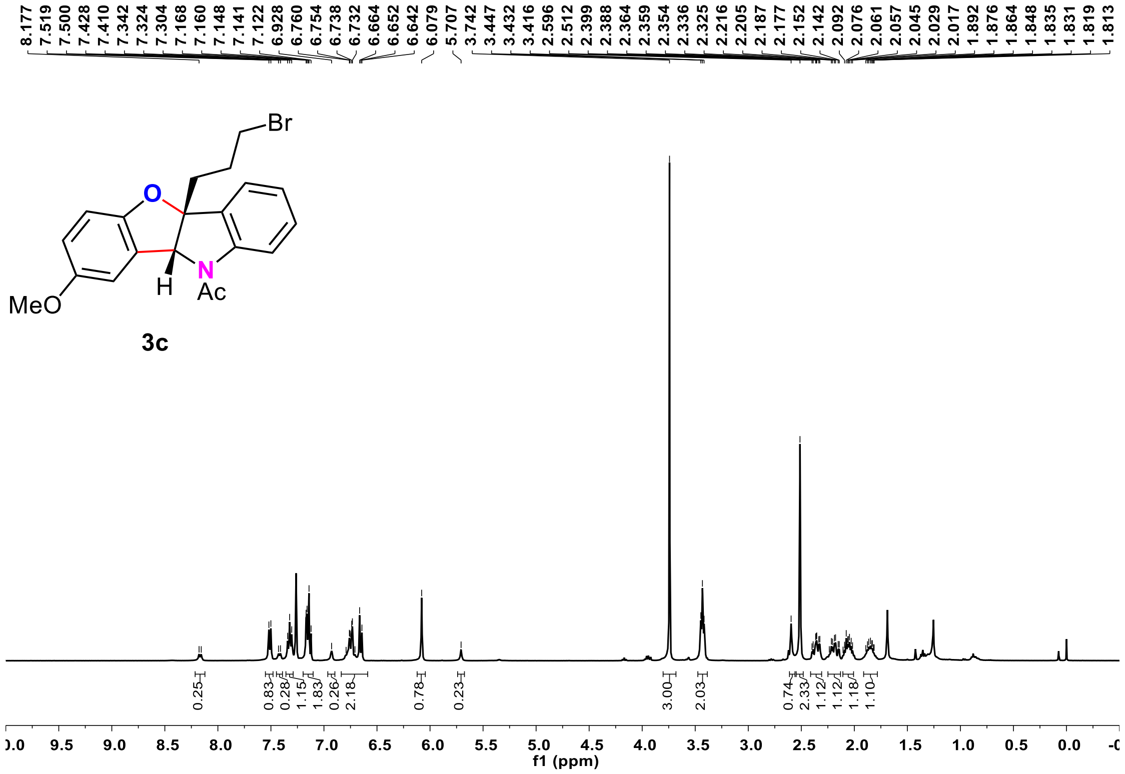

Supplementary Figure 5. **^{1}H NMR (400 MHz, $CDCl_3$) spectrum of 3c**

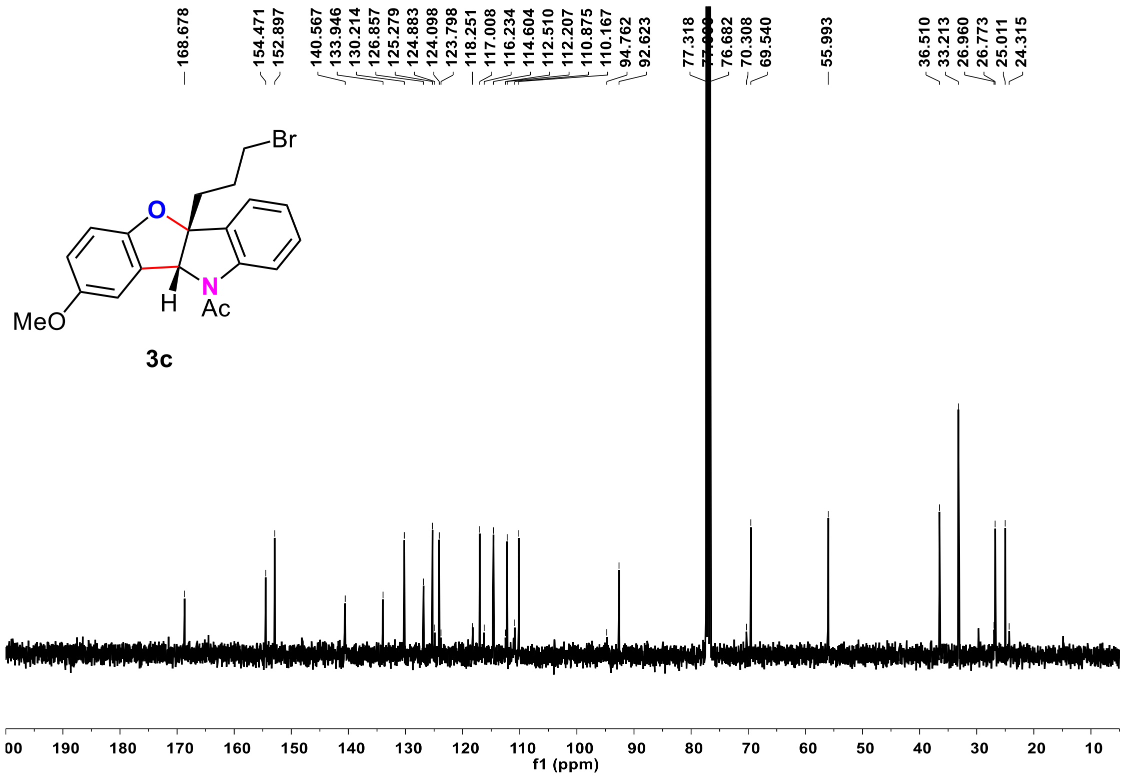

Supplementary Figure 6. **^{13}C NMR (101 MHz, $CDCl_3$) spectrum of 3c**

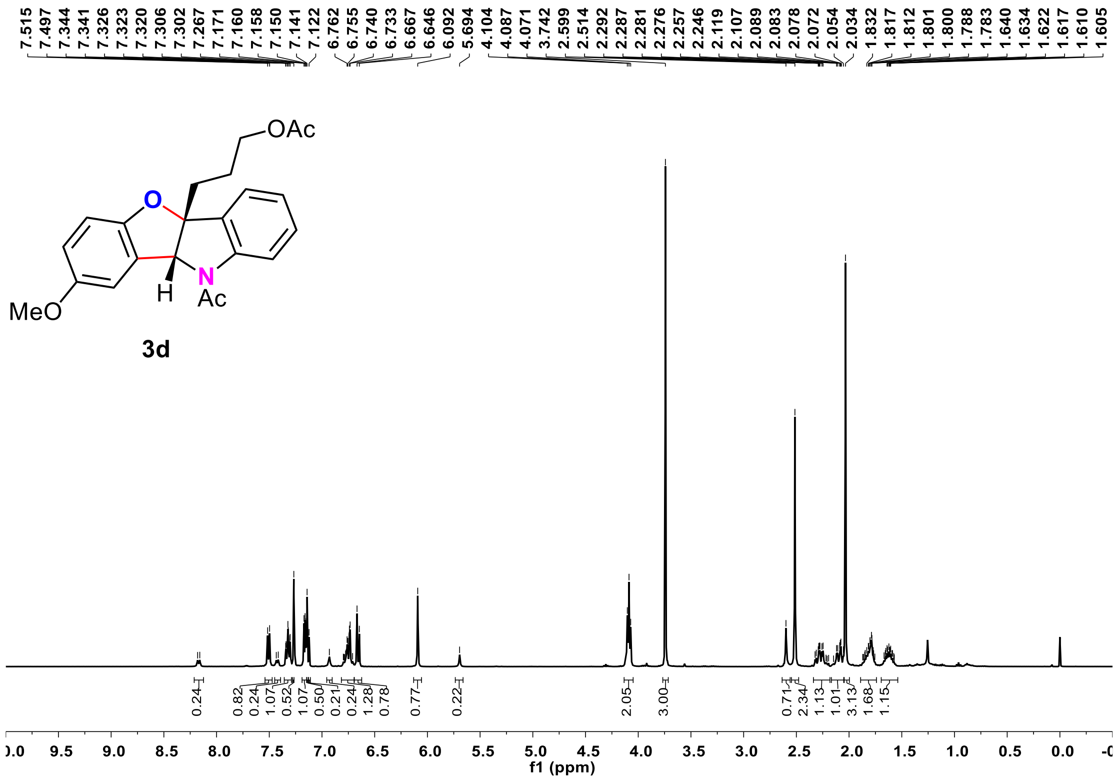

Supplementary Figure 7. ^{1}H NMR (400 MHz, $CDCl_3$) spectrum of 3d

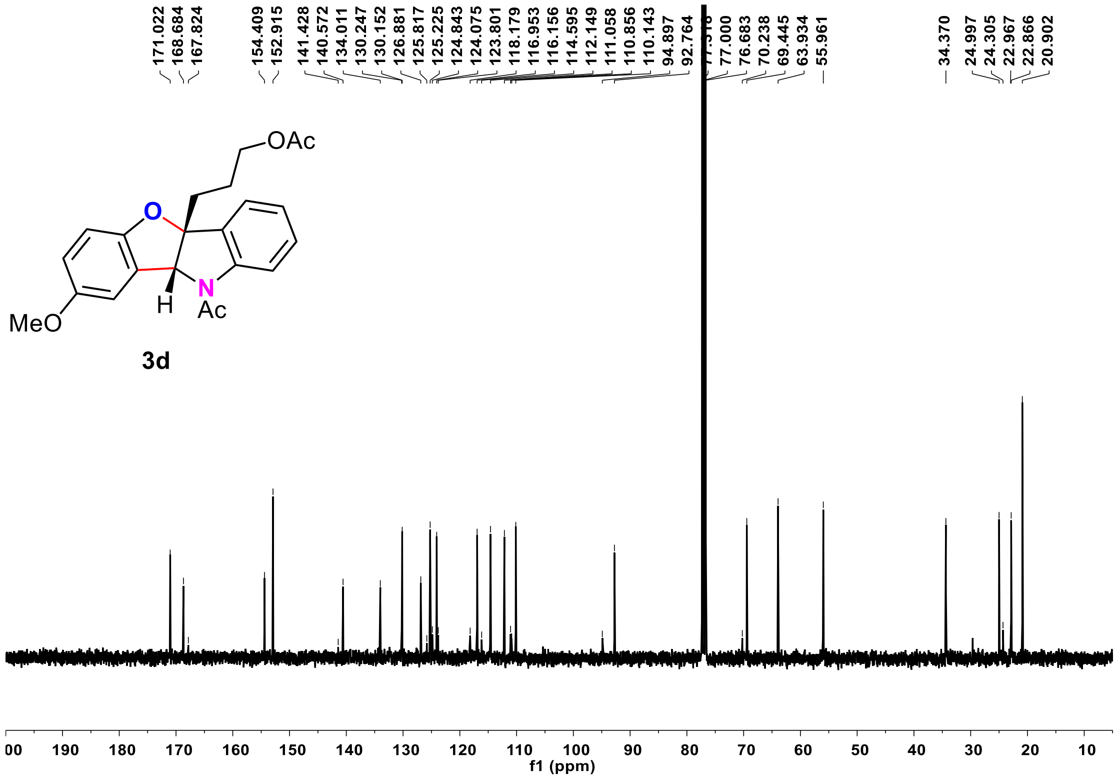

Supplementary Figure 8. ^{13}C NMR (101 MHz, $CDCl_3$) spectrum of 3d

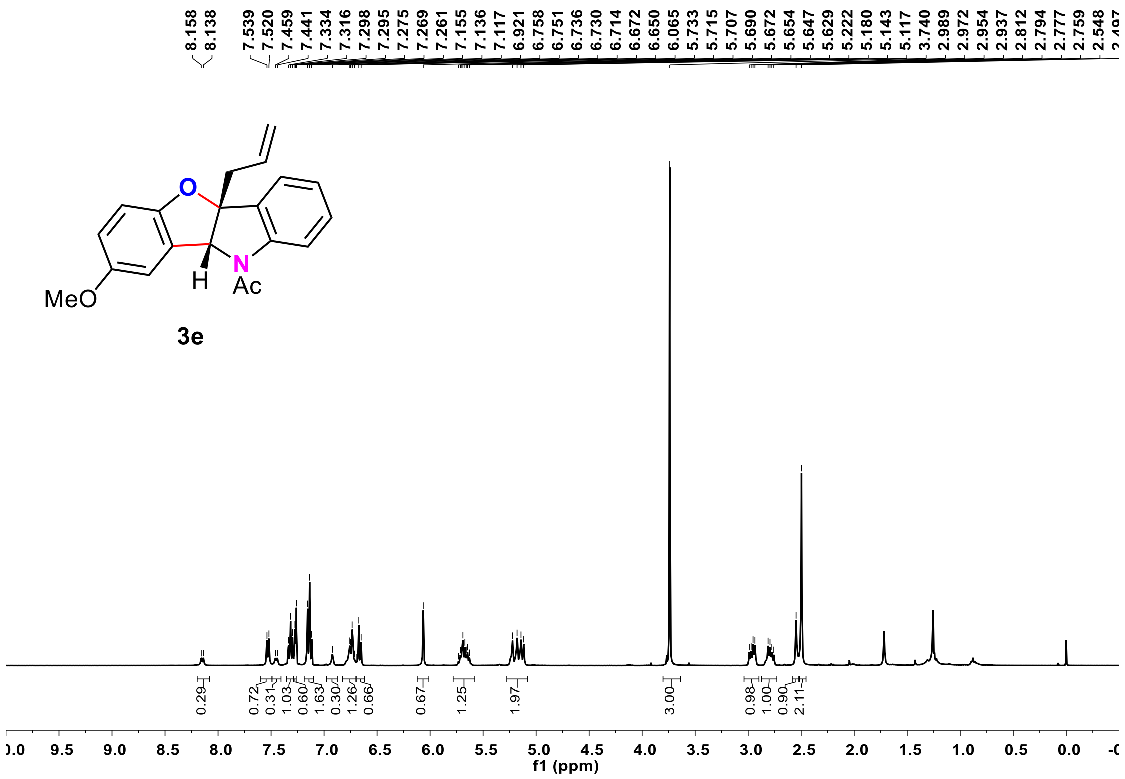

Supplementary Figure 9. ^{1}H **NMR (400 MHz,** $CDCl_3$**) spectrum of 3e**

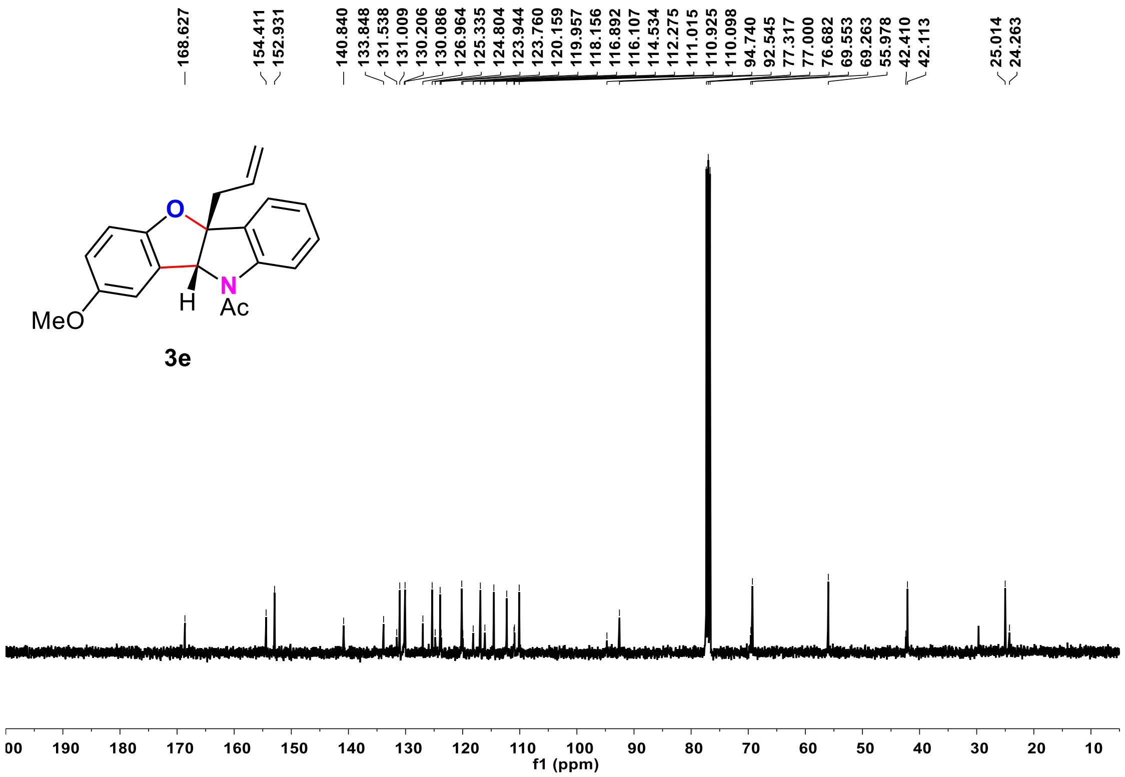

Supplementary Figure 10. ^{13}C **NMR (101 MHz,** $CDCl_3$**) spectrum of 3e**

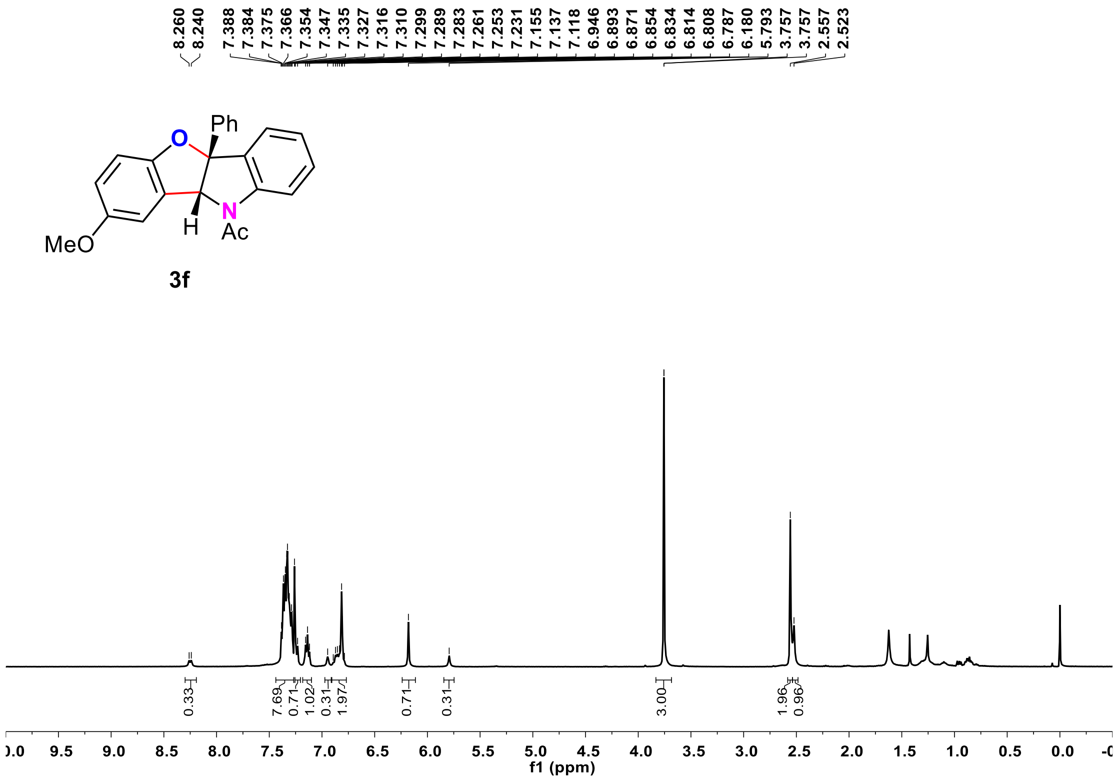

Supplementary Figure 11. ^{1}H NMR (400 MHz, $CDCl_3$) **spectrum of 3f**

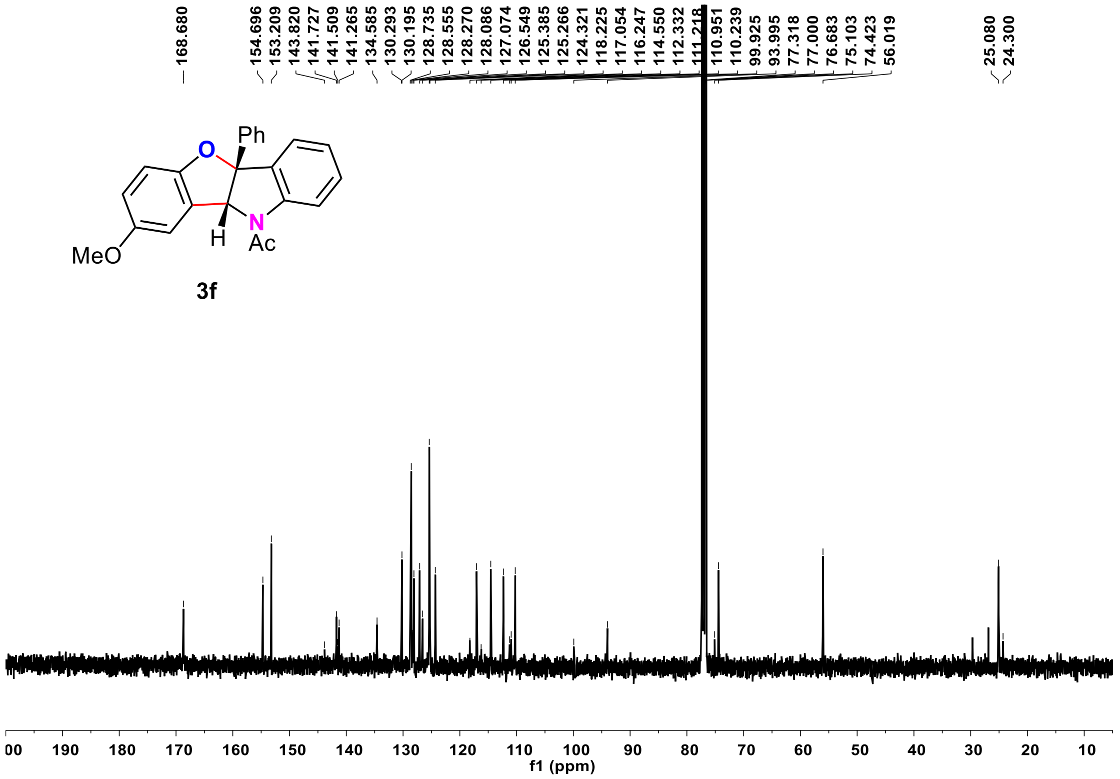

Supplementary Figure 12. ^{13}C NMR (101 MHz, $CDCl_3$) **spectrum of 3f**

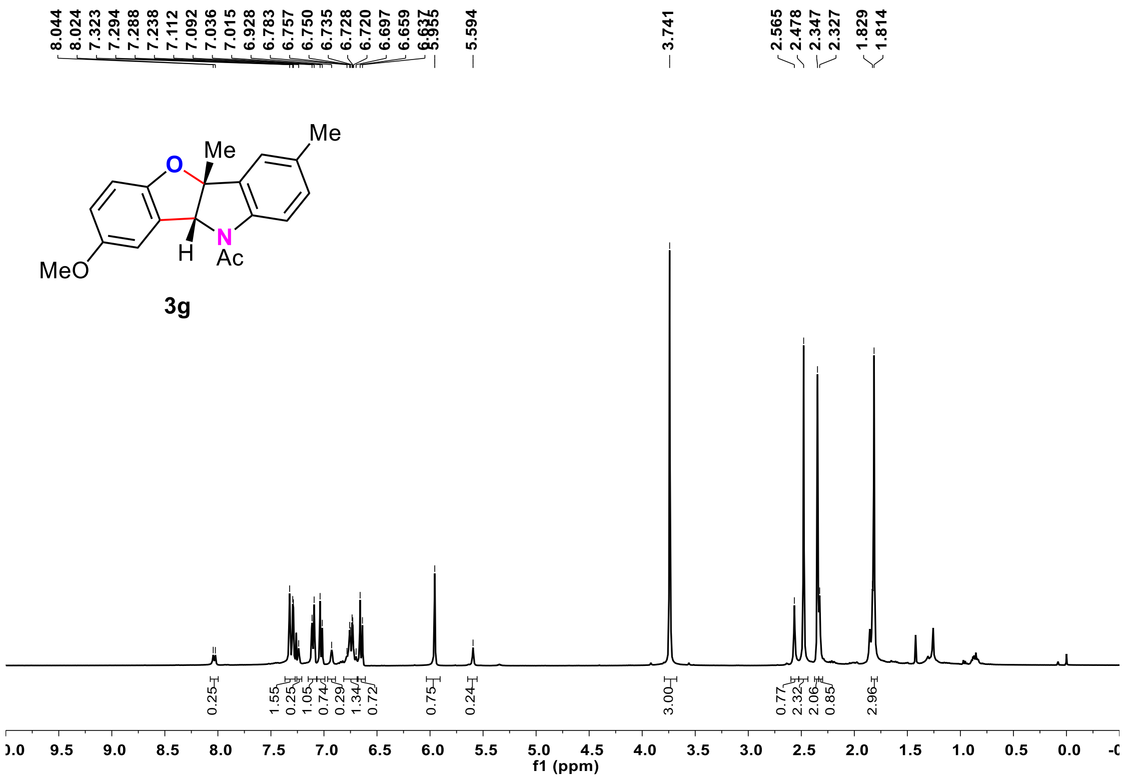

Supplementary Figure 13. ^{1}**H NMR (400 MHz, CDCl$_3$) spectrum of 3g**

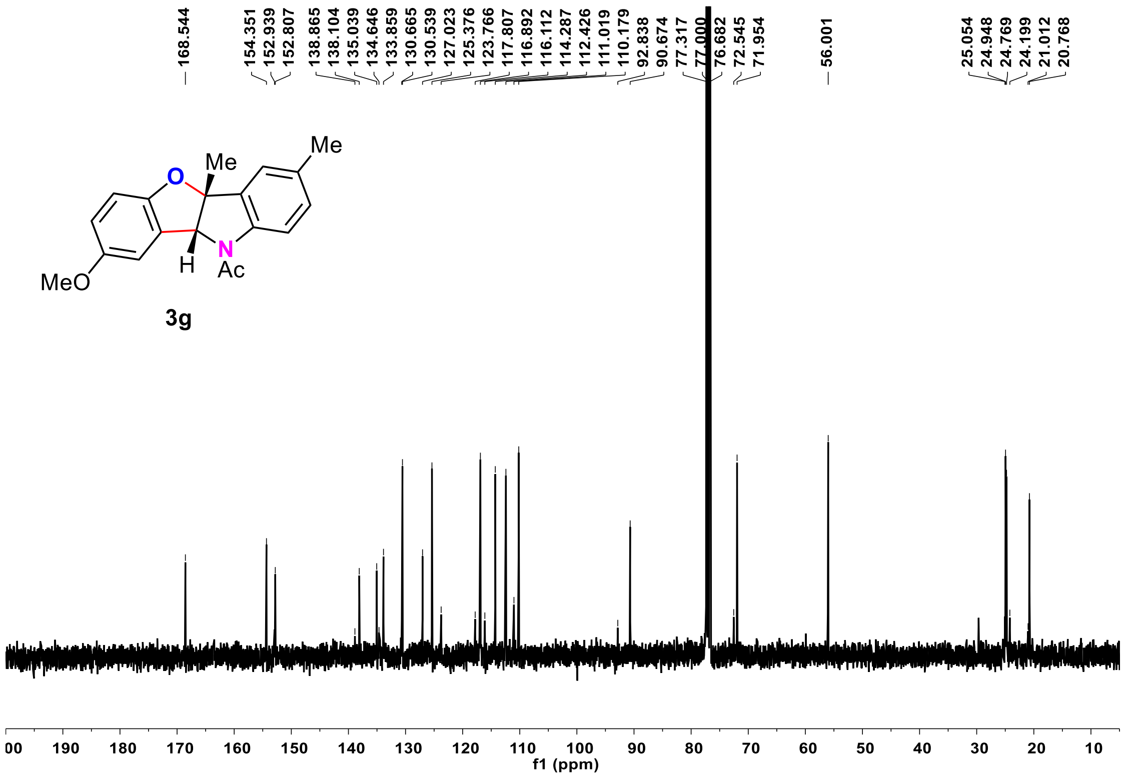

Supplementary Figure 14. ^{13}**C NMR (101 MHz, CDCl$_3$) spectrum of 3g**

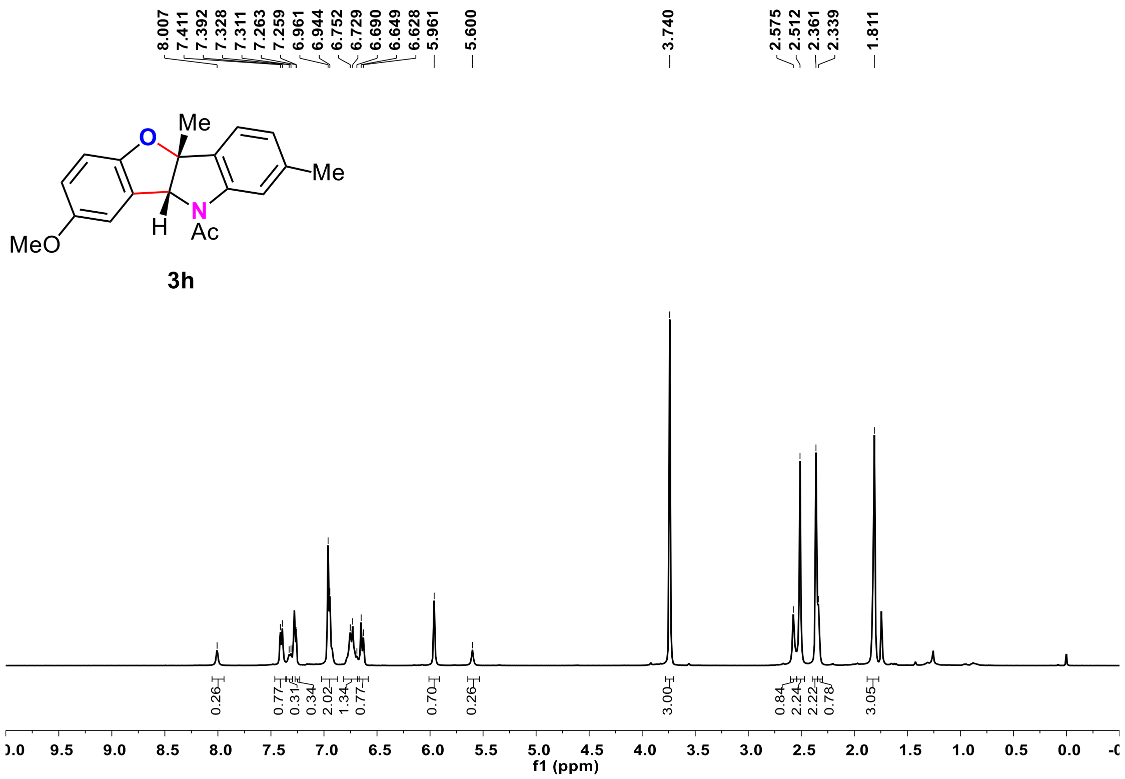

Supplementary Figure 15. ^{1}H NMR (400 MHz, $CDCl_3$) spectrum of 3h

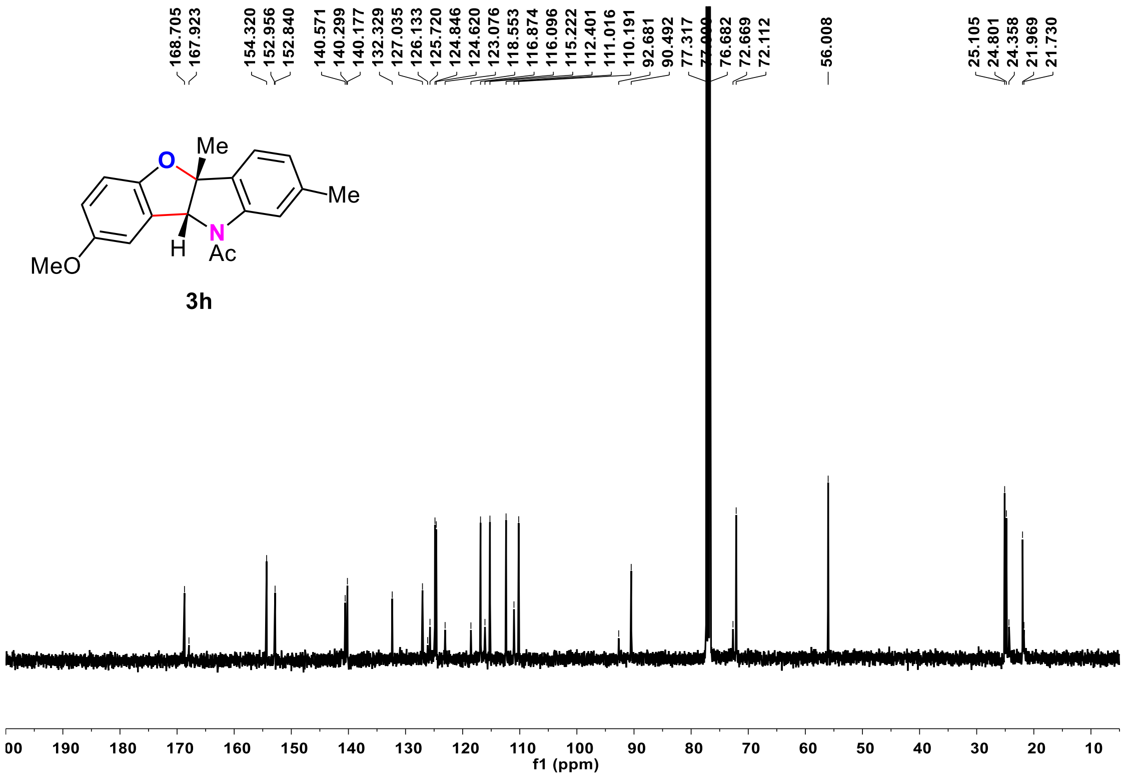

Supplementary Figure 16. ^{13}C NMR (101 MHz, $CDCl_3$) spectrum of 3h

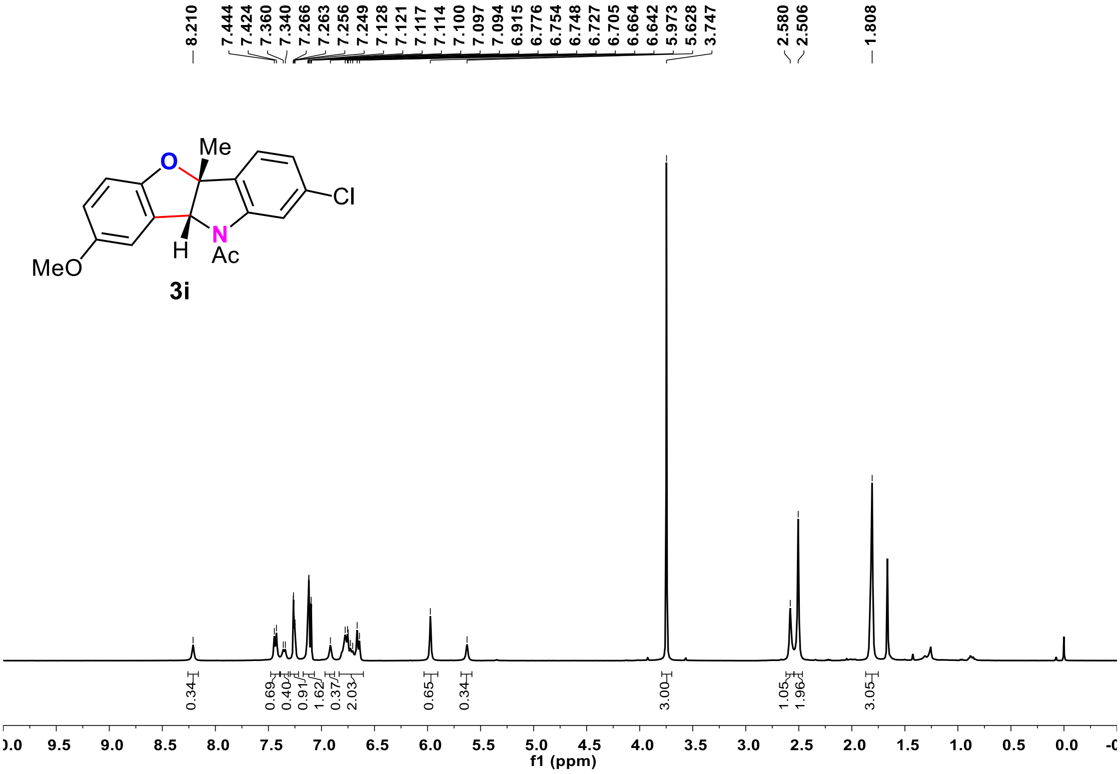

Supplementary Figure 17. ^{1}H NMR (400 MHz, $CDCl_3$) spectrum of 3i

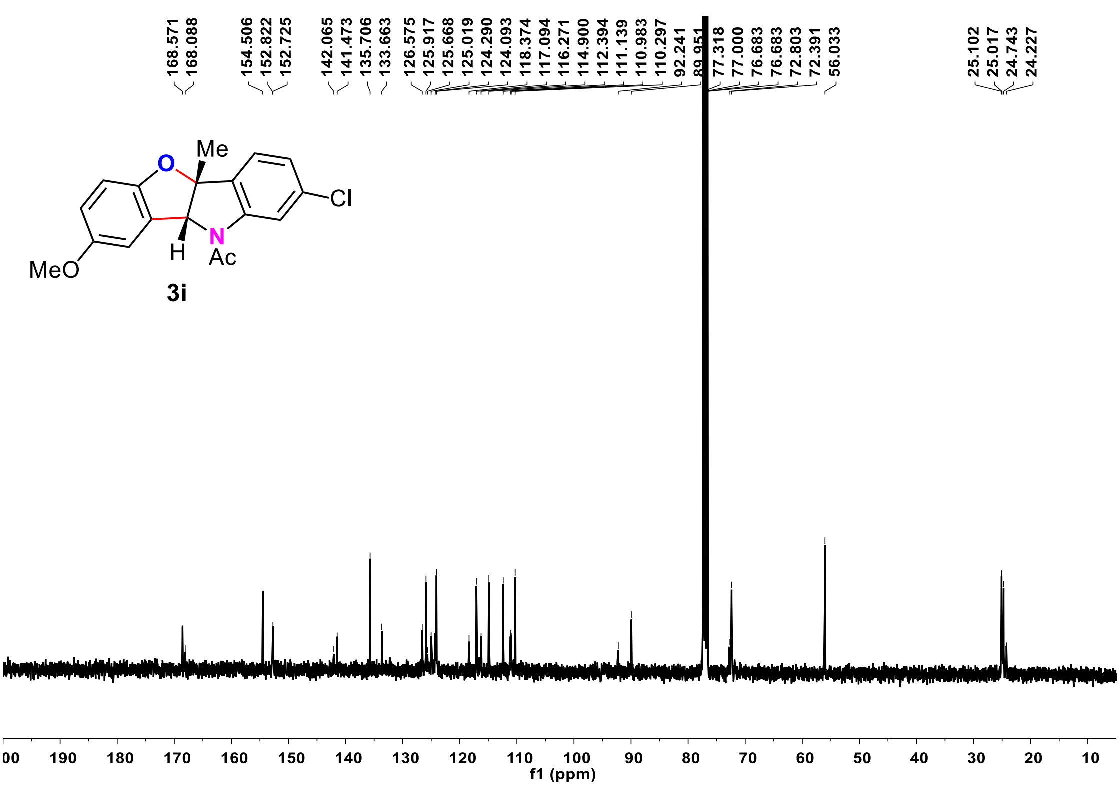

Supplementary Figure 18. ^{13}C NMR (101 MHz, $CDCl_3$) spectrum of 3i

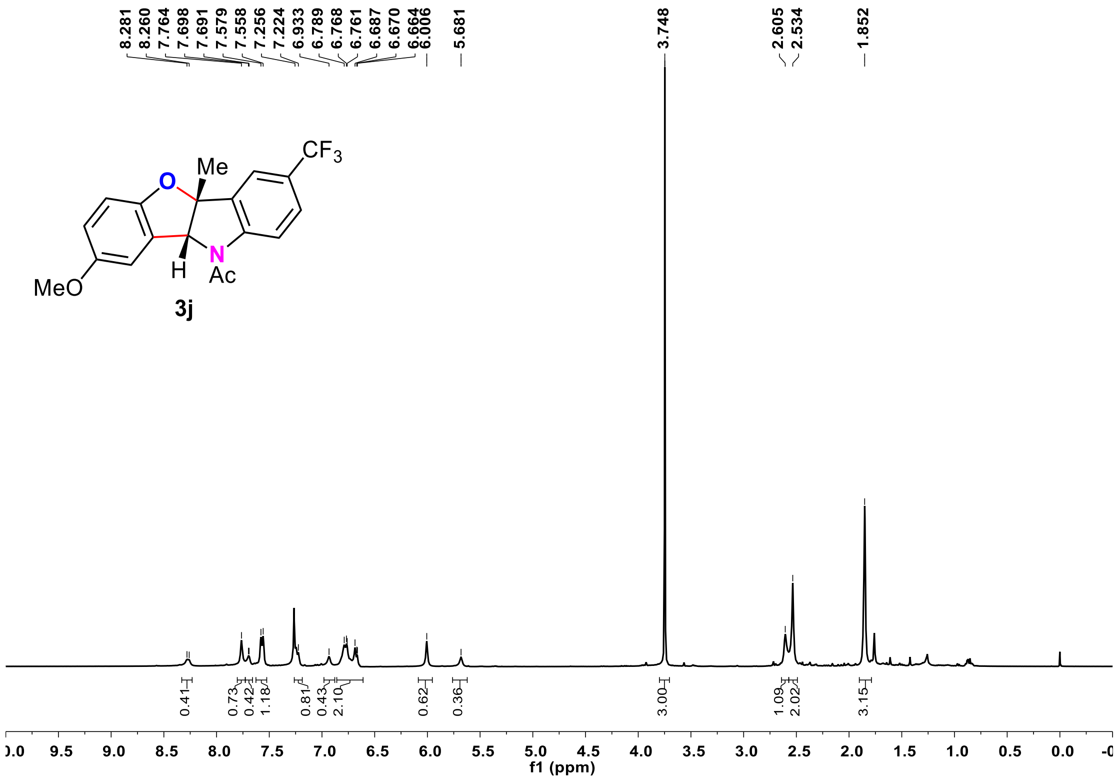

Supplementary Figure 19. ^{1}H NMR (400 MHz, $CDCl_3$) spectrum of 3j

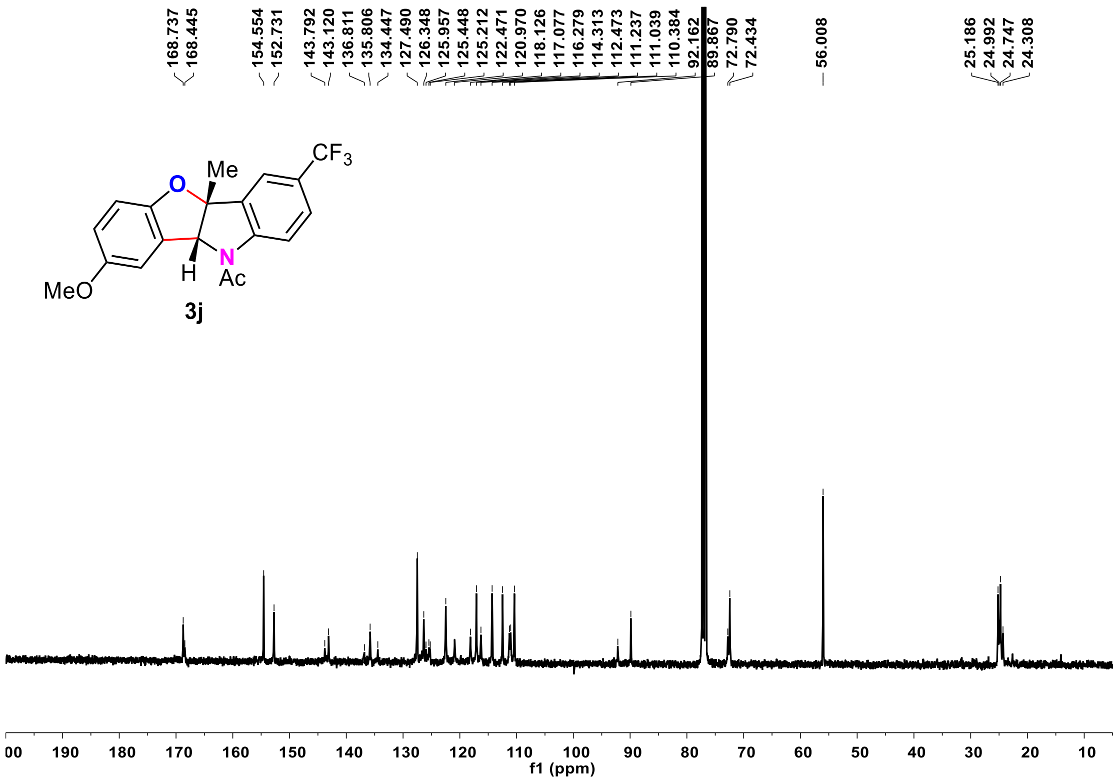

Supplementary Figure 20. ^{13}C NMR (101 MHz, $CDCl_3$) spectrum of 3j

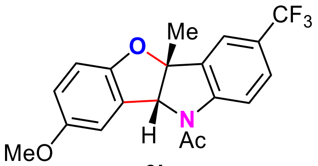

3j

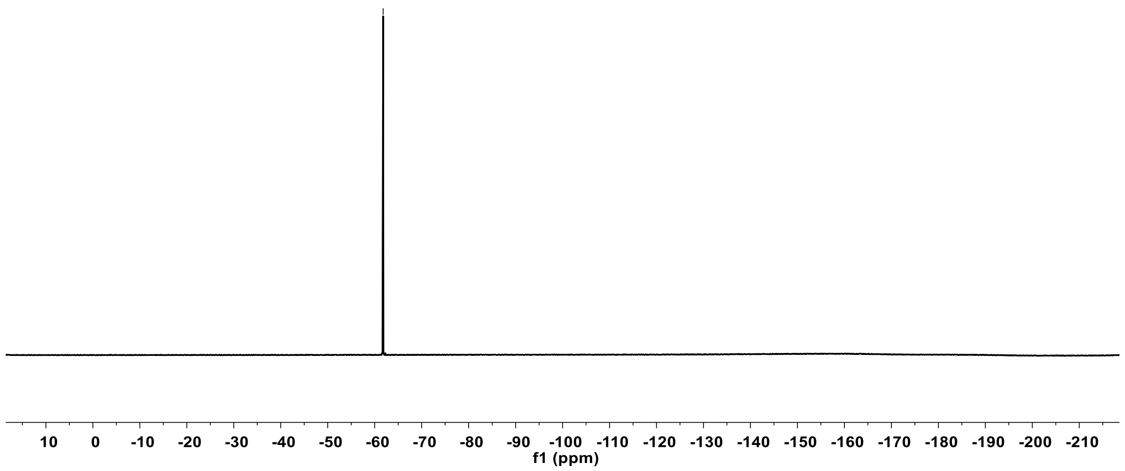

Supplementary Figure 21. ^{19}F NMR (377 MHz, $CDCl_3$) spectrum of 3j

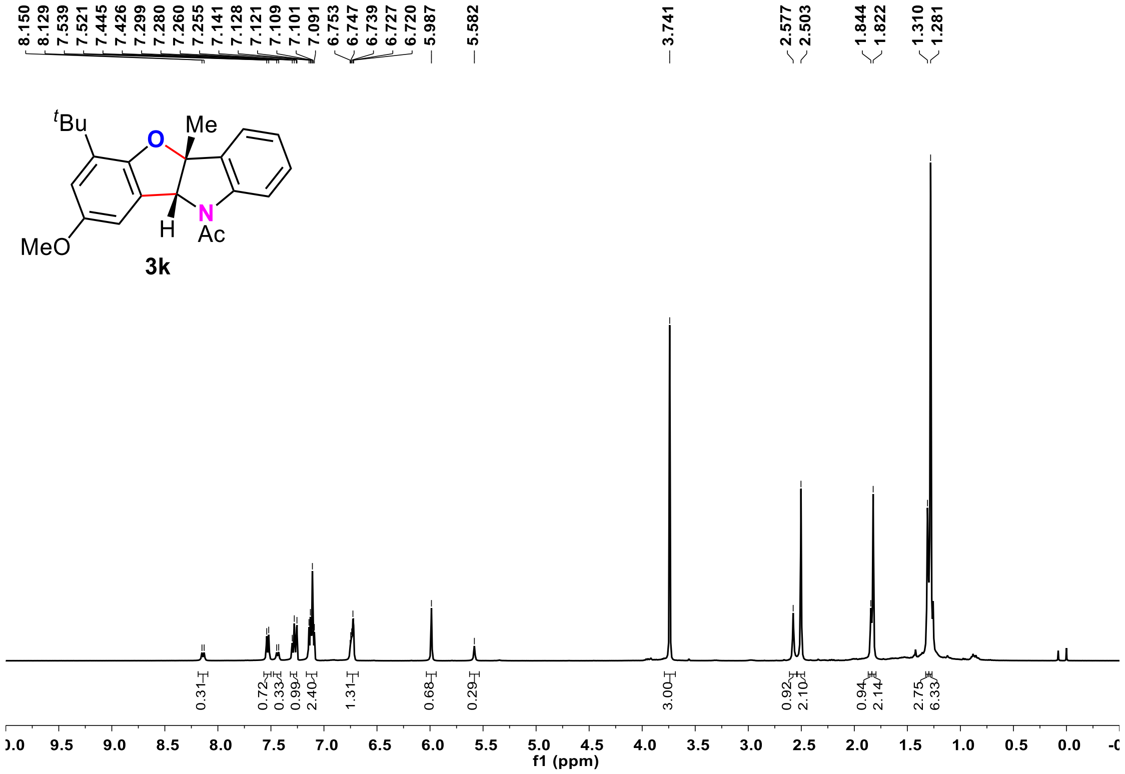

Supplementary Figure 22. ^{1}H NMR (400 MHz, $CDCl_3$) spectrum of 3k

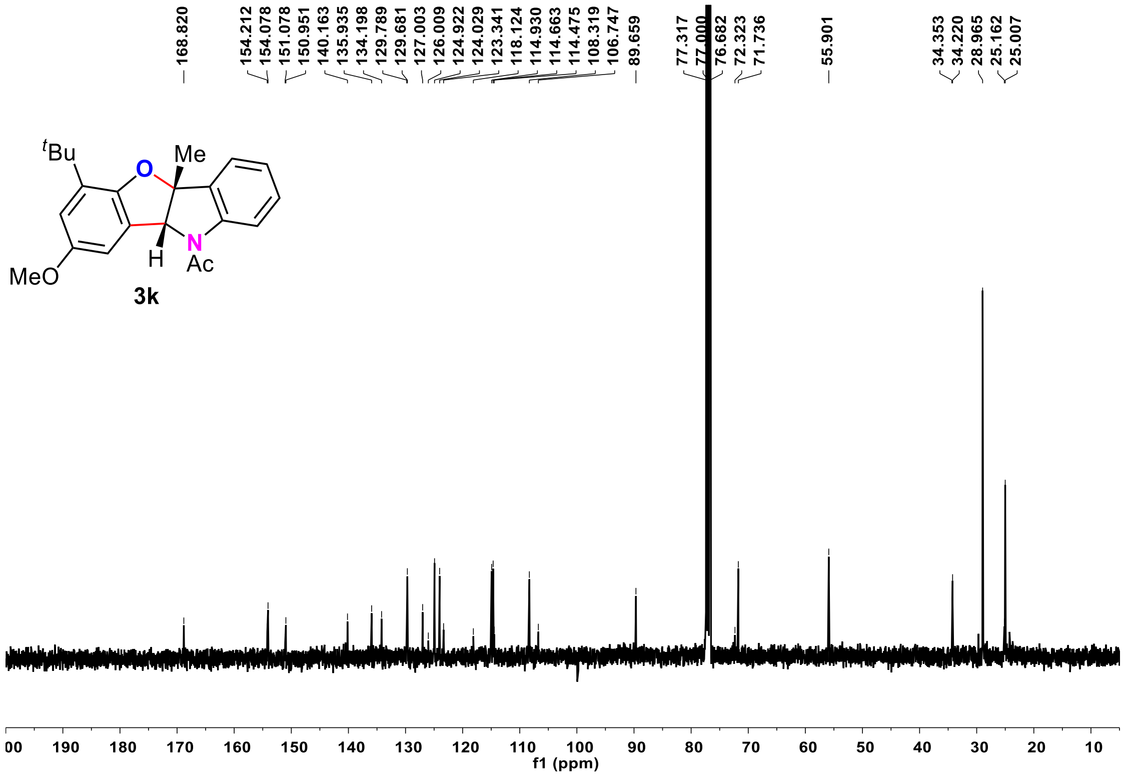

Supplementary Figure 23. ^{13}C NMR (101 MHz, $CDCl_3$) spectrum of 3k

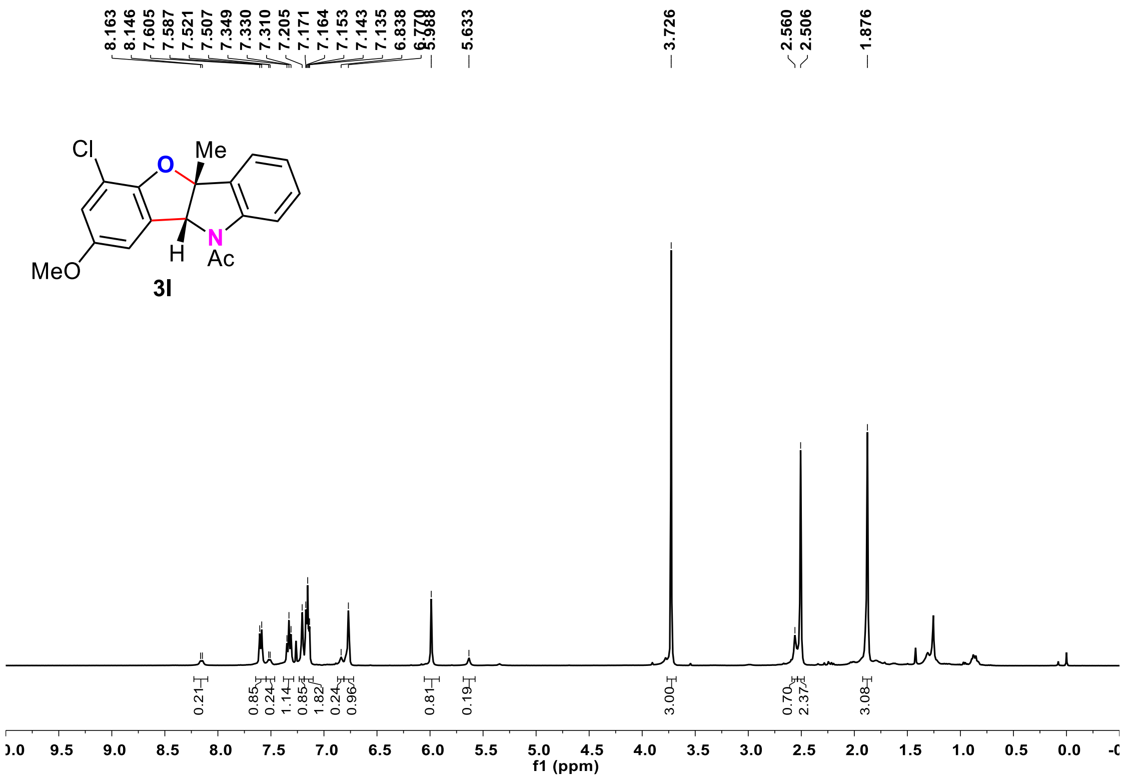

Supplementary Figure 24. ^{1}H NMR (400 MHz, $CDCl_3$) spectrum of 3l

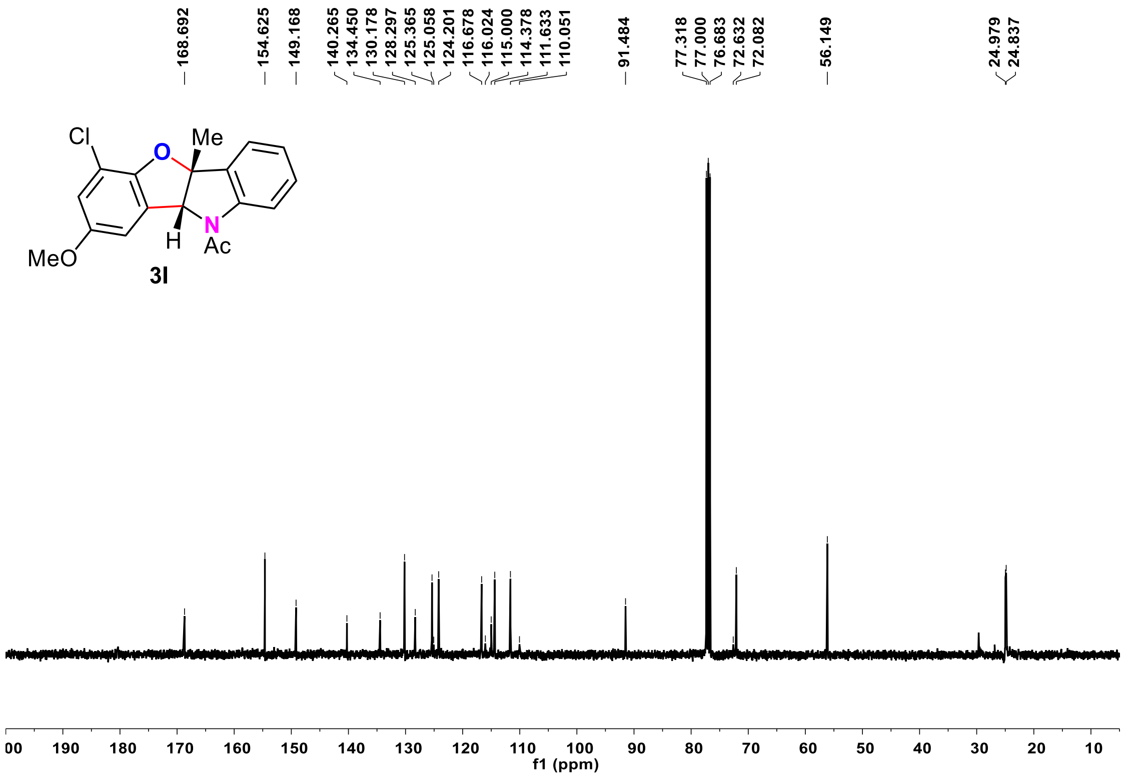

Supplementary Figure 25. ^{13}C NMR (101 MHz, $CDCl_3$) spectrum of 3l

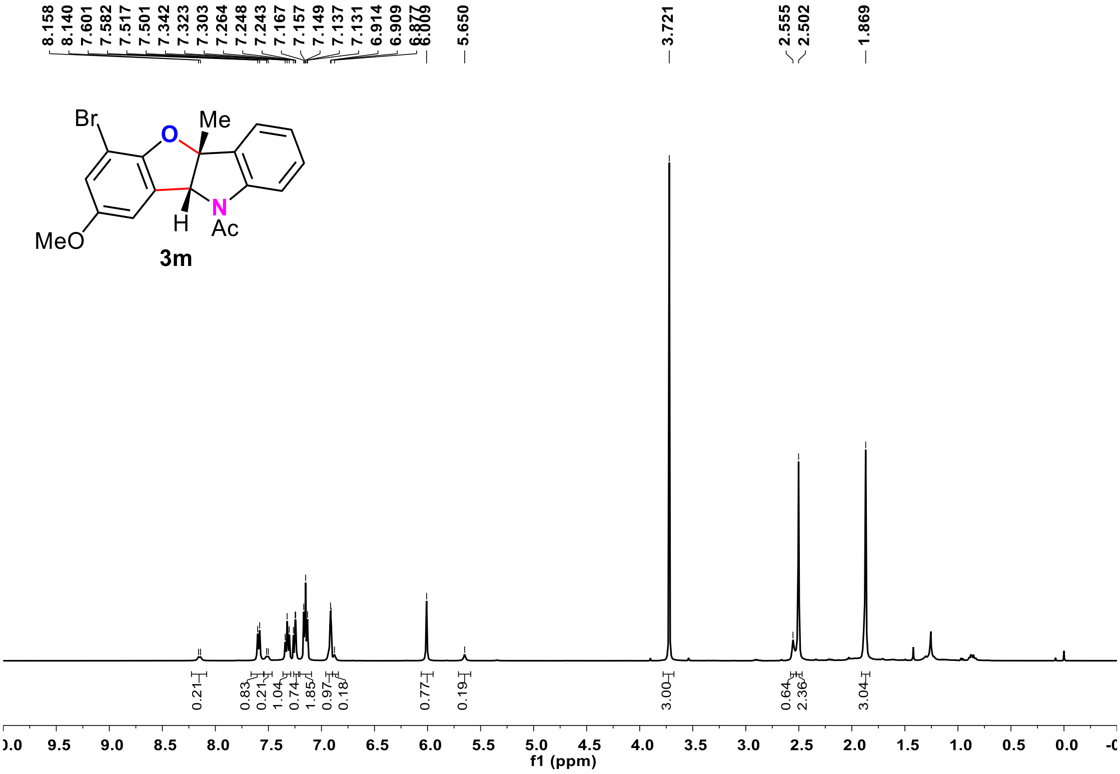

Supplementary Figure 26. ^{1}H NMR (400 MHz, $CDCl_3$) spectrum of 3m

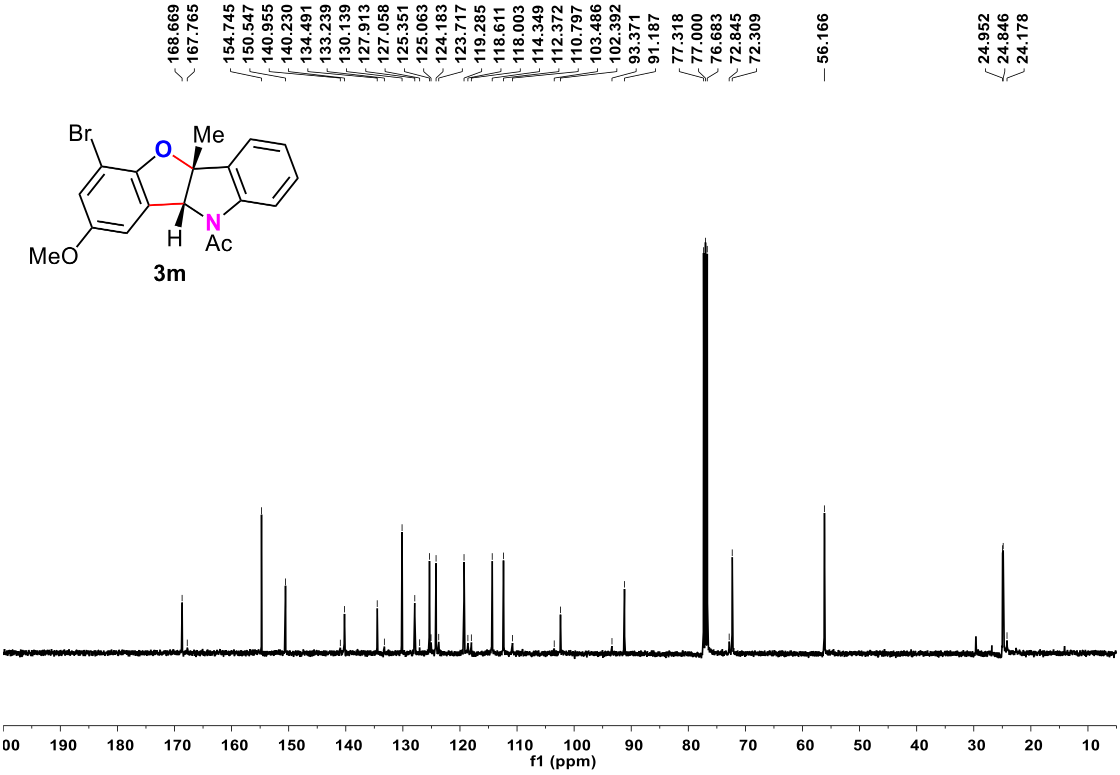

Supplementary Figure 27. ^{13}C NMR (101 MHz, $CDCl_3$) spectrum of 3m

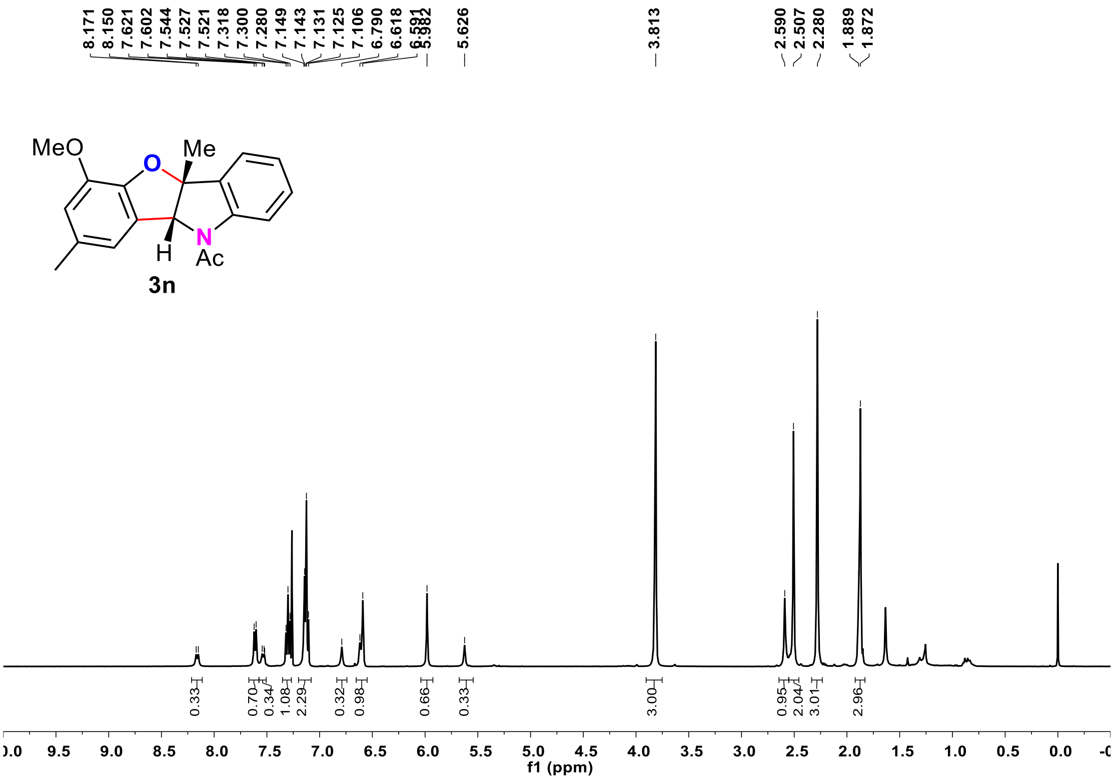

Supplementary Figure 28. ^{1}H **NMR (400 MHz,** $CDCl_3$**) spectrum of 3n**

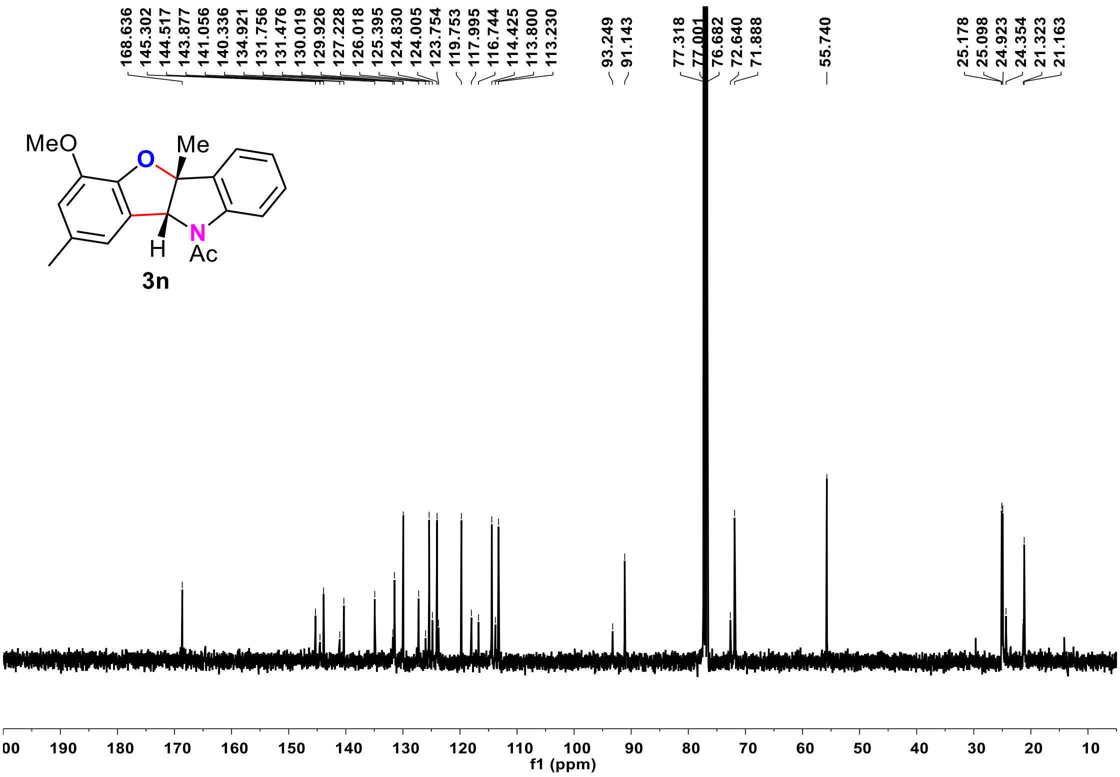

Supplementary Figure 29. ^{13}C **NMR (101 MHz,** $CDCl_3$**) spectrum of 3n**

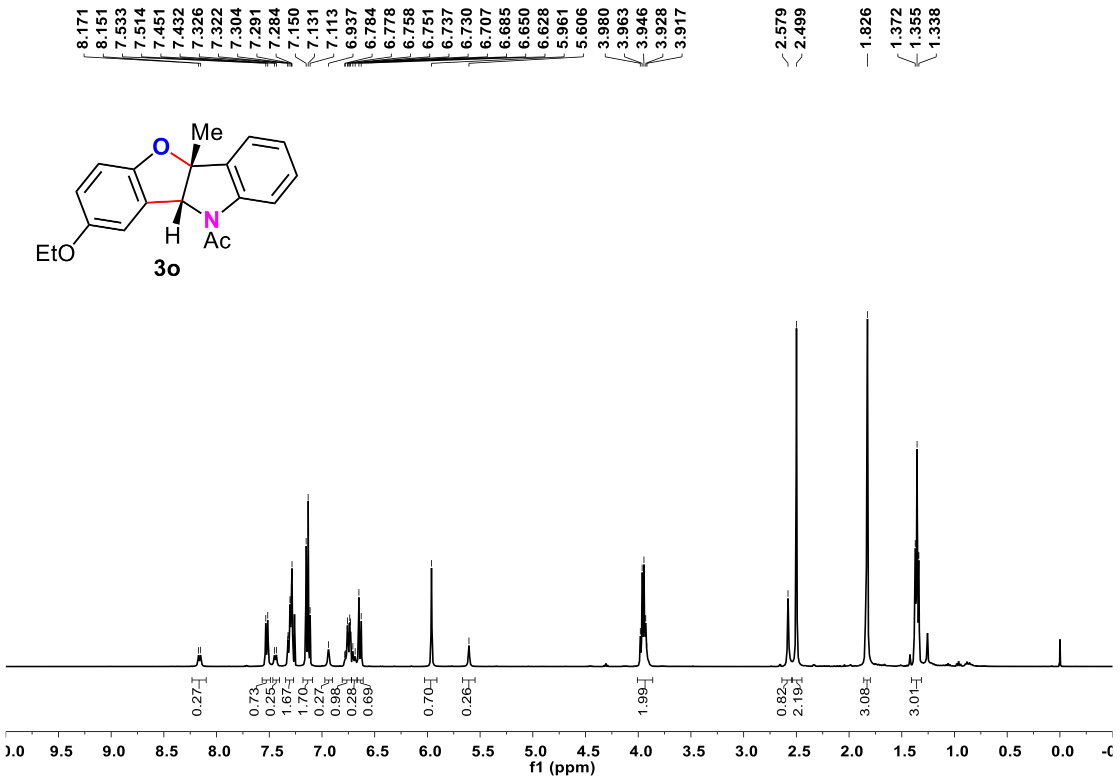

Supplementary Figure 30. ^{1}H NMR (400 MHz, $CDCl_3$) spectrum of 3o

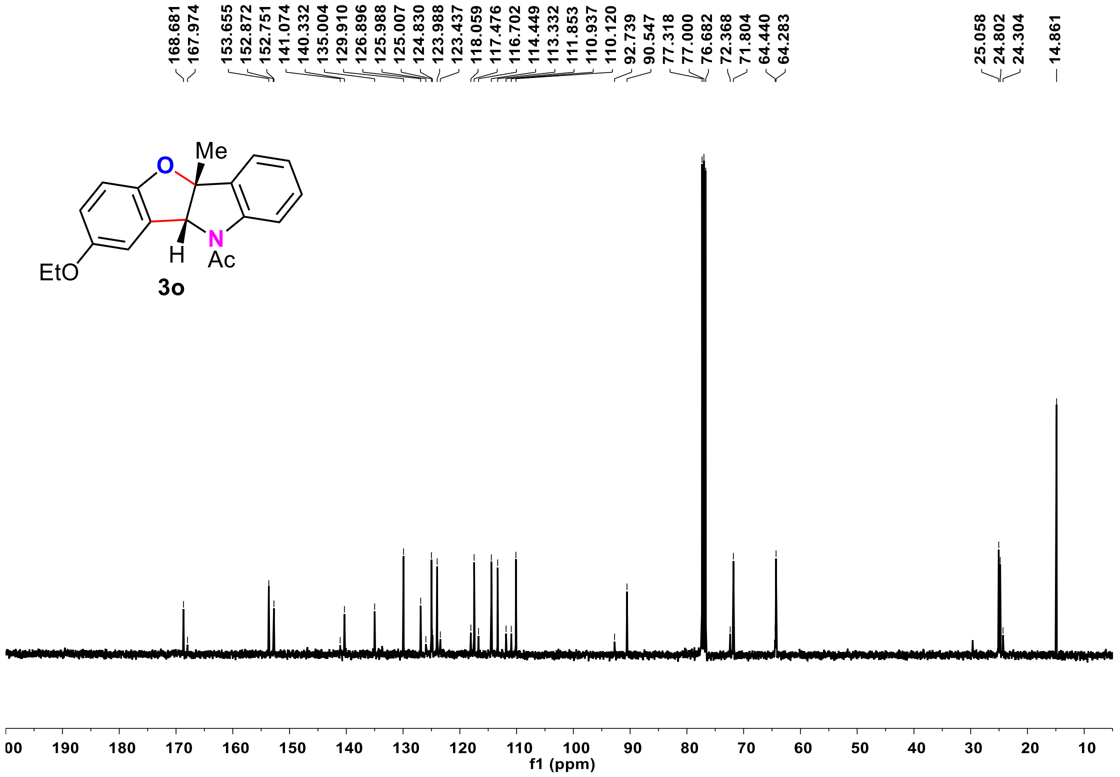

Supplementary Figure 31. ^{13}C NMR (101 MHz, $CDCl_3$) spectrum of 3o

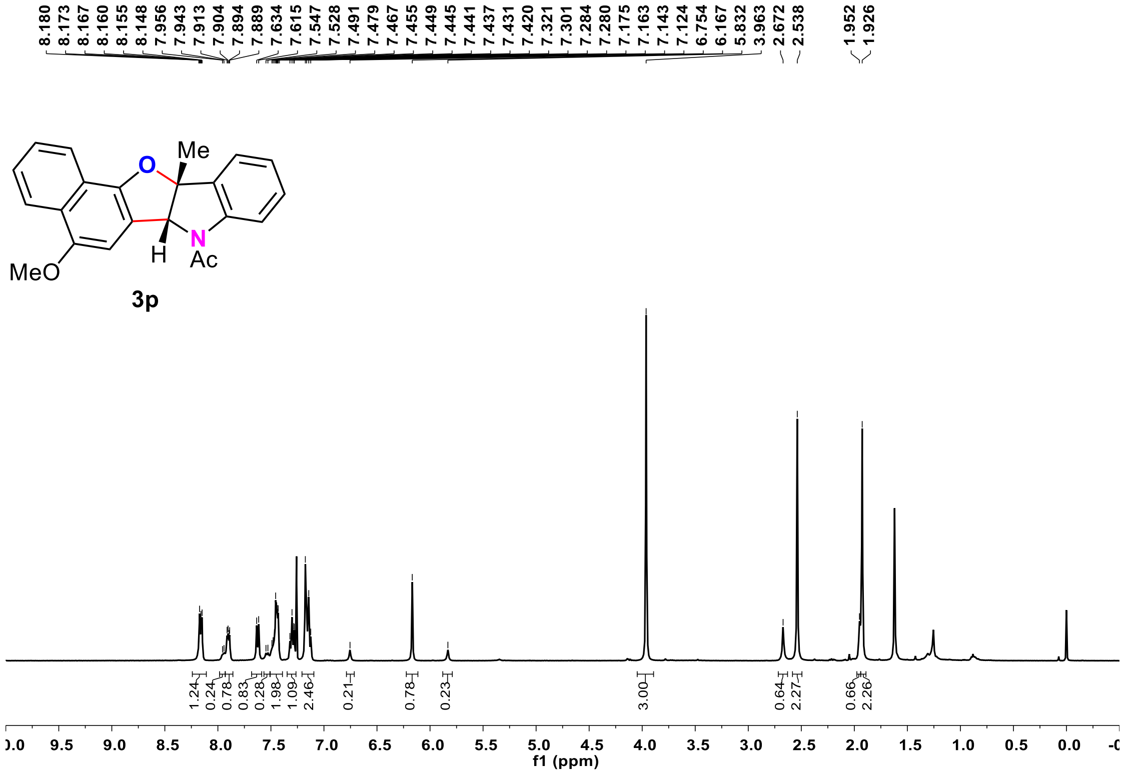

Supplementary Figure 32. ^{1}H **NMR (400 MHz,** $CDCl_3$**) spectrum of 3p**

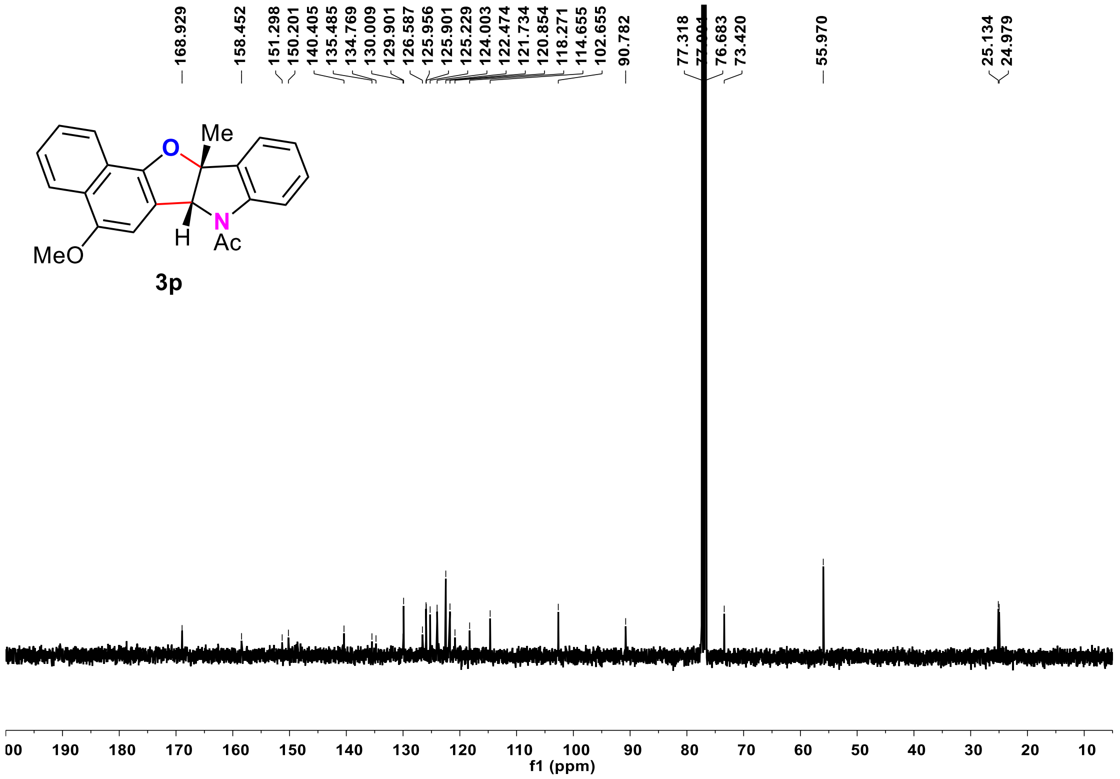

Supplementary Figure 33. ^{13}C **NMR (101 MHz,** $CDCl_3$**) spectrum of 3p**

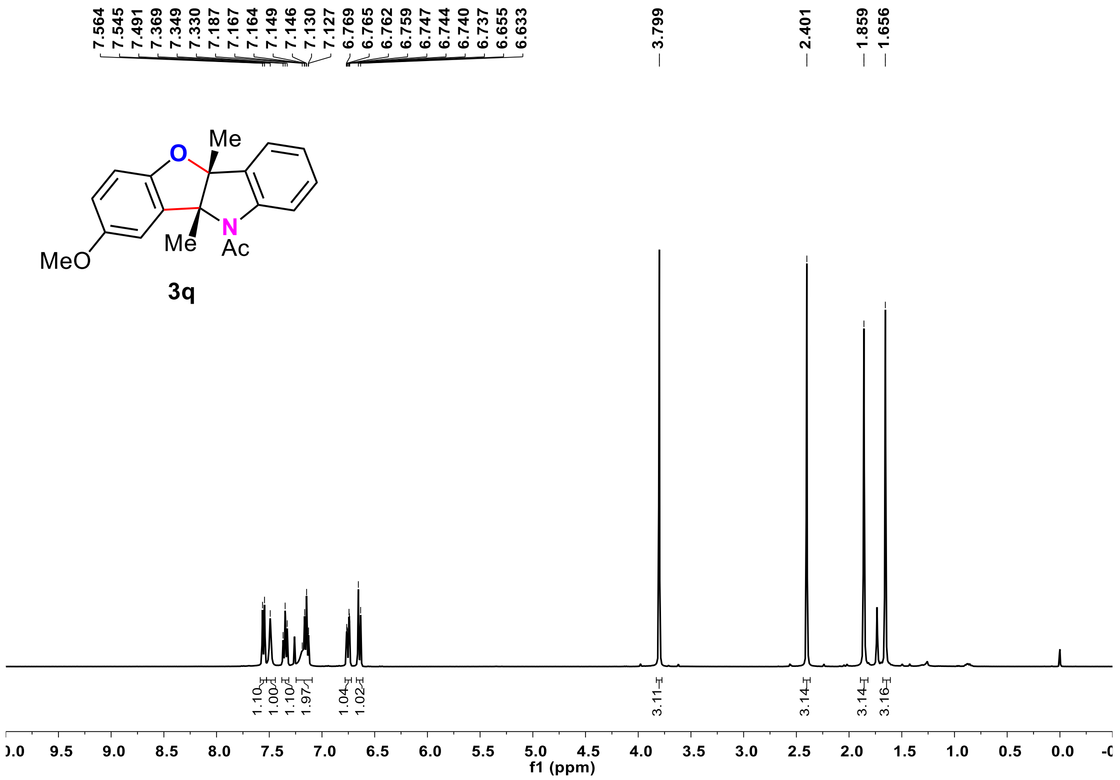

Supplementary Figure 34. ^{1}H **NMR (400 MHz,** $CDCl_3$**) spectrum of 3q**

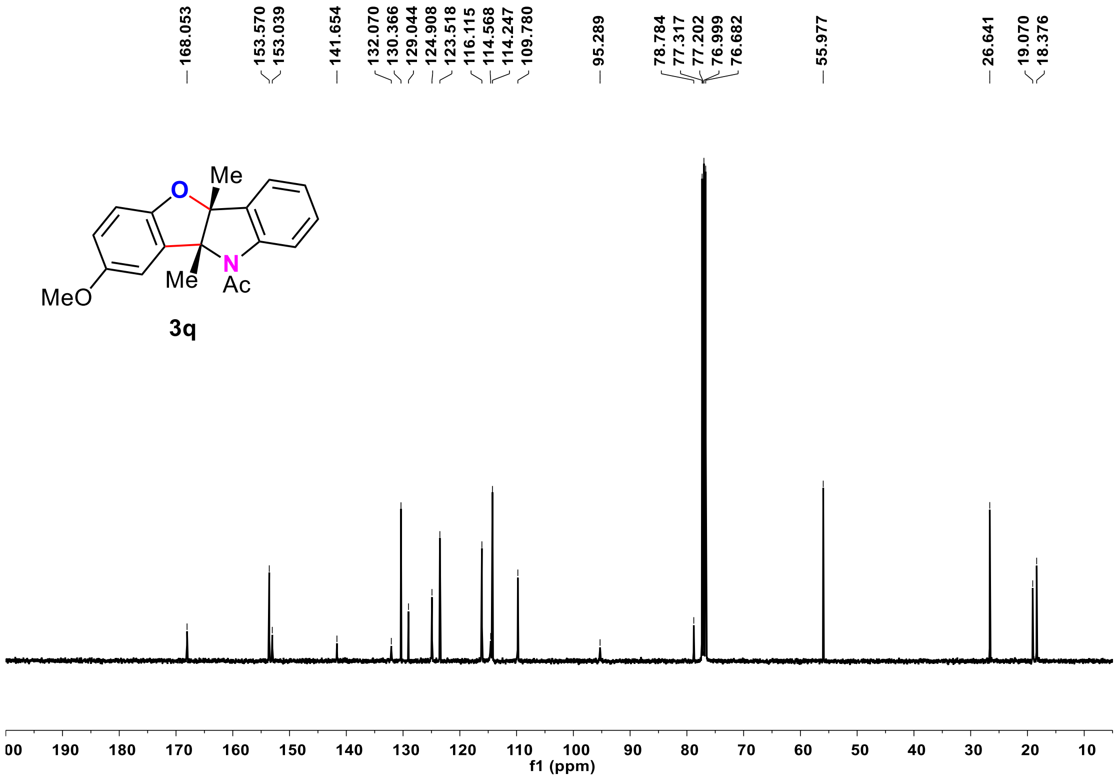

Supplementary Figure 35. ^{13}C **NMR (101 MHz,** $CDCl_3$**) spectrum of 3q**

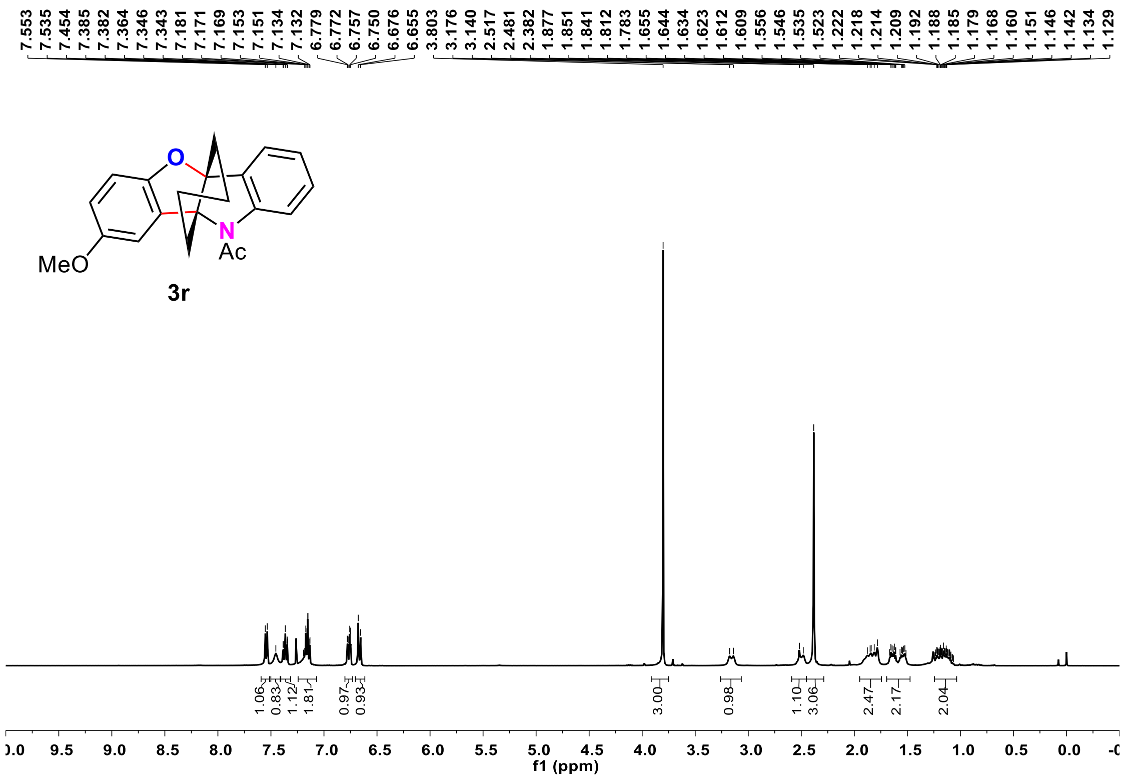

Supplementary Figure 36. ^{1}H **NMR (400 MHz,** $CDCl_3$**) spectrum of 3r**

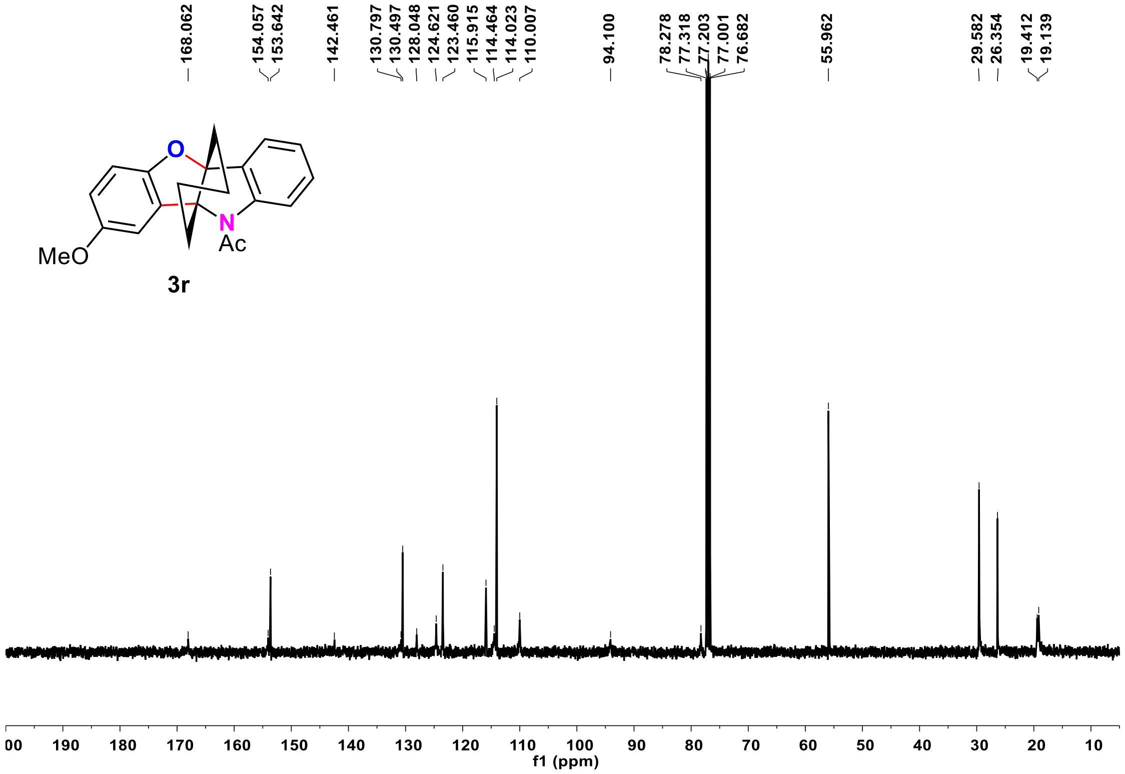

Supplementary Figure 37. ^{13}C **NMR (101 MHz,** $CDCl_3$**) spectrum of 3r**

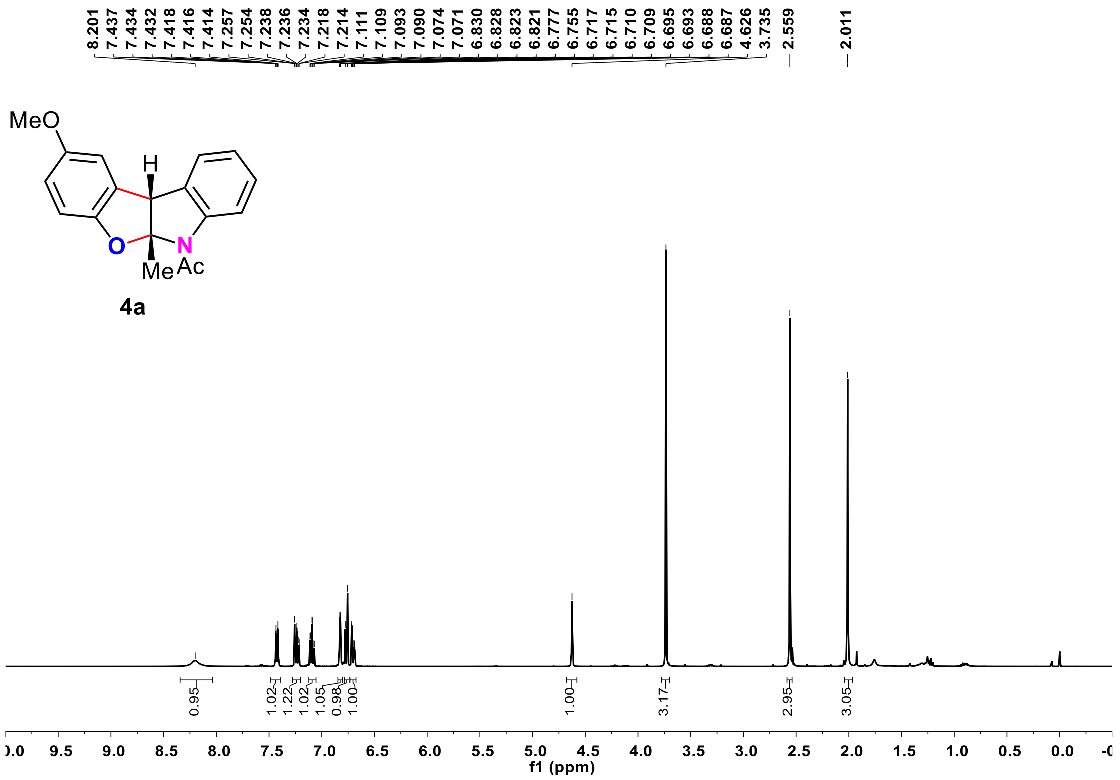

Supplementary Figure 38. ^{1}H NMR (400 MHz, $CDCl_3$) spectrum of 4a

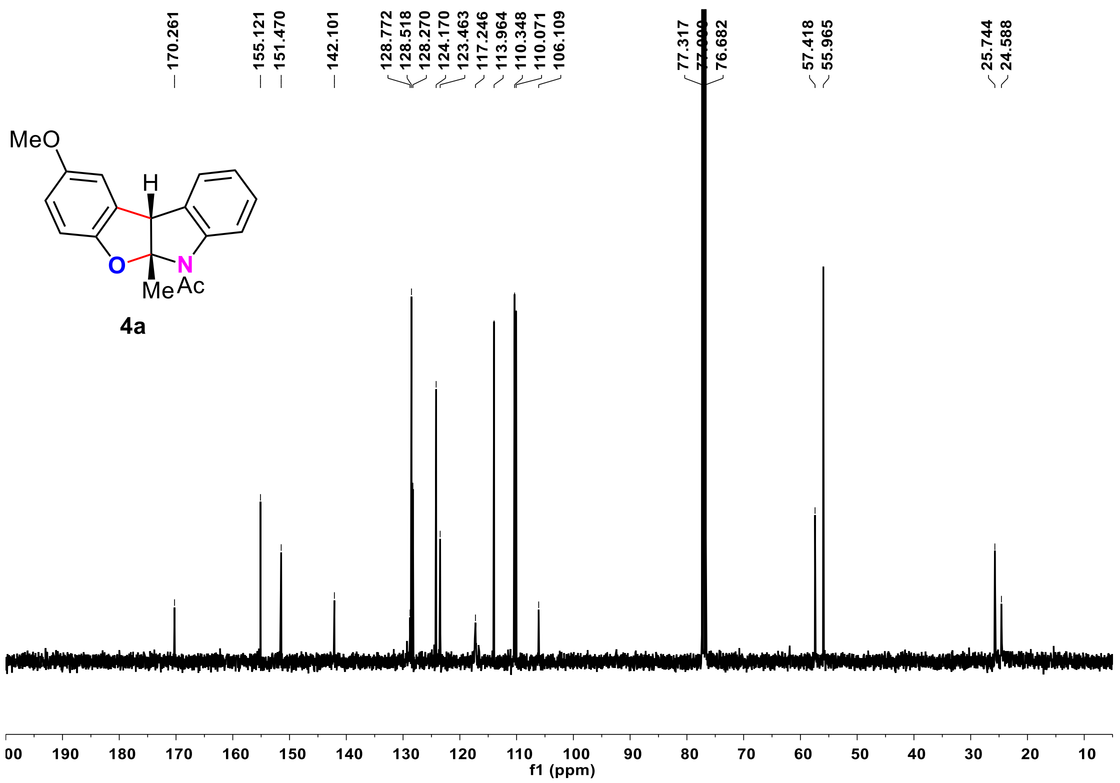

Supplementary Figure 39. ^{13}C NMR (101 MHz, $CDCl_3$) spectrum of 4a

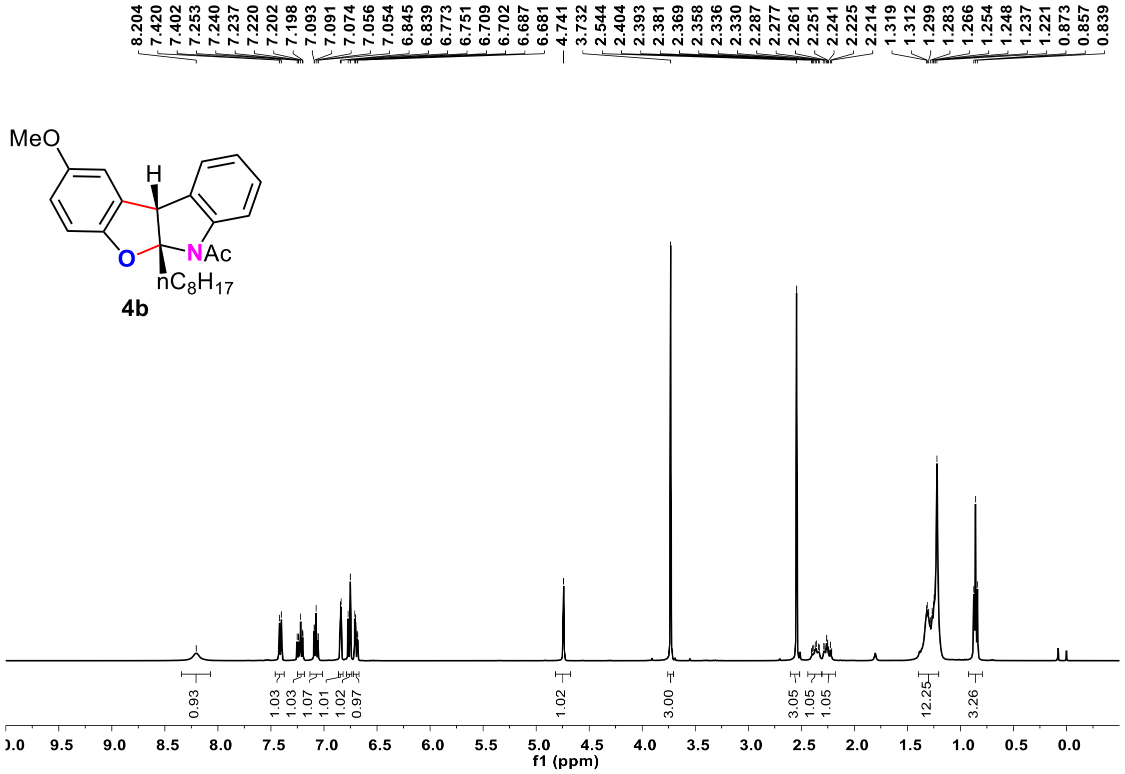

Supplementary Figure 40. ^{1}H NMR (400 MHz, $CDCl_3$) spectrum of 4b

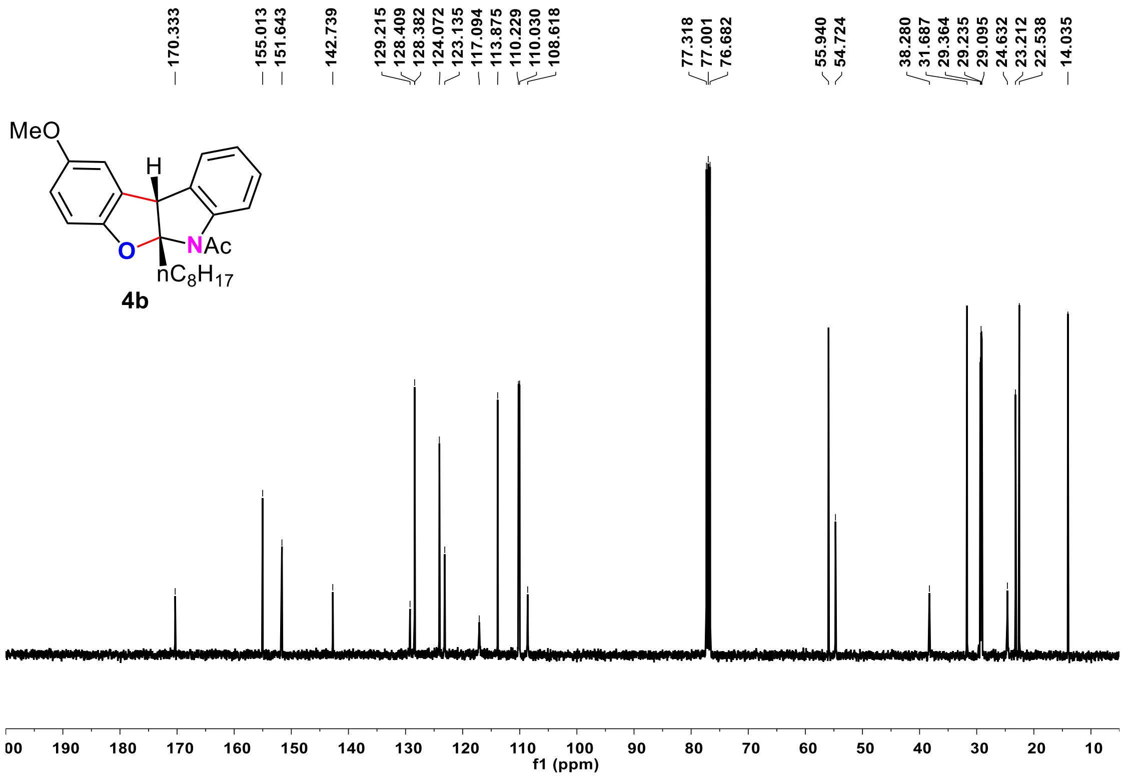

Supplementary Figure 41. ^{13}C NMR (101 MHz, $CDCl_3$) spectrum of 4b

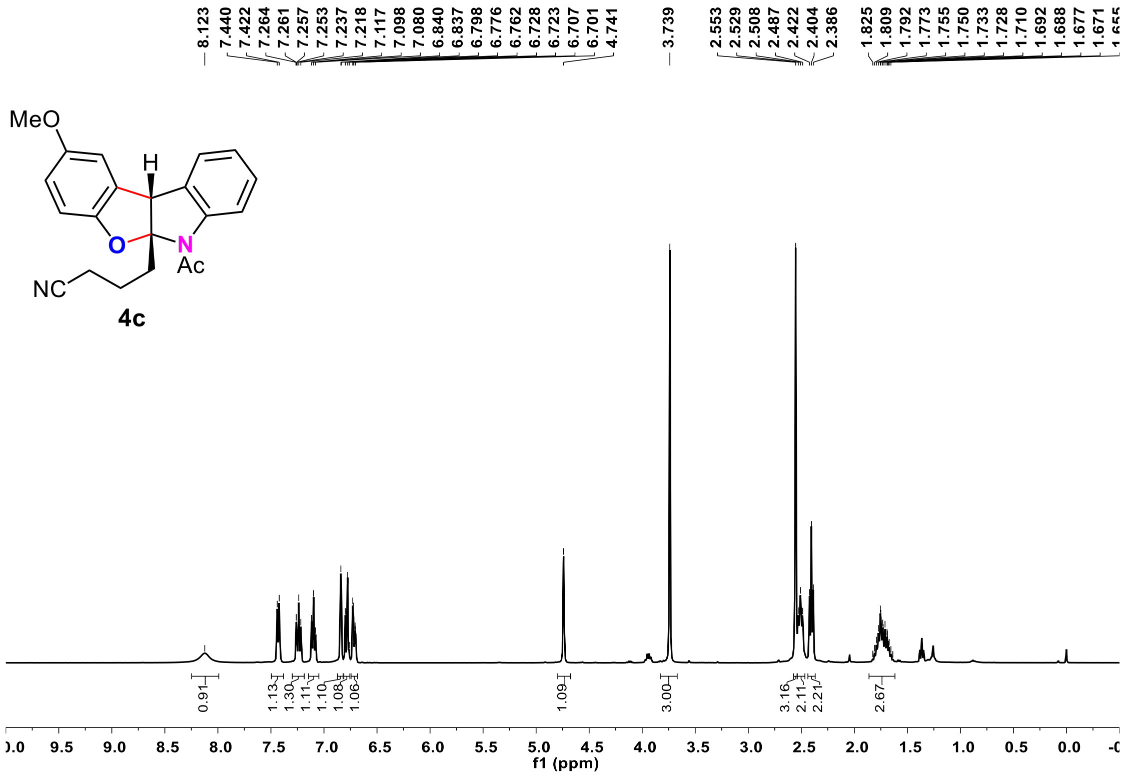

Supplementary Figure 42. ^{1}H NMR (400 MHz, $CDCl_3$) spectrum of 4c

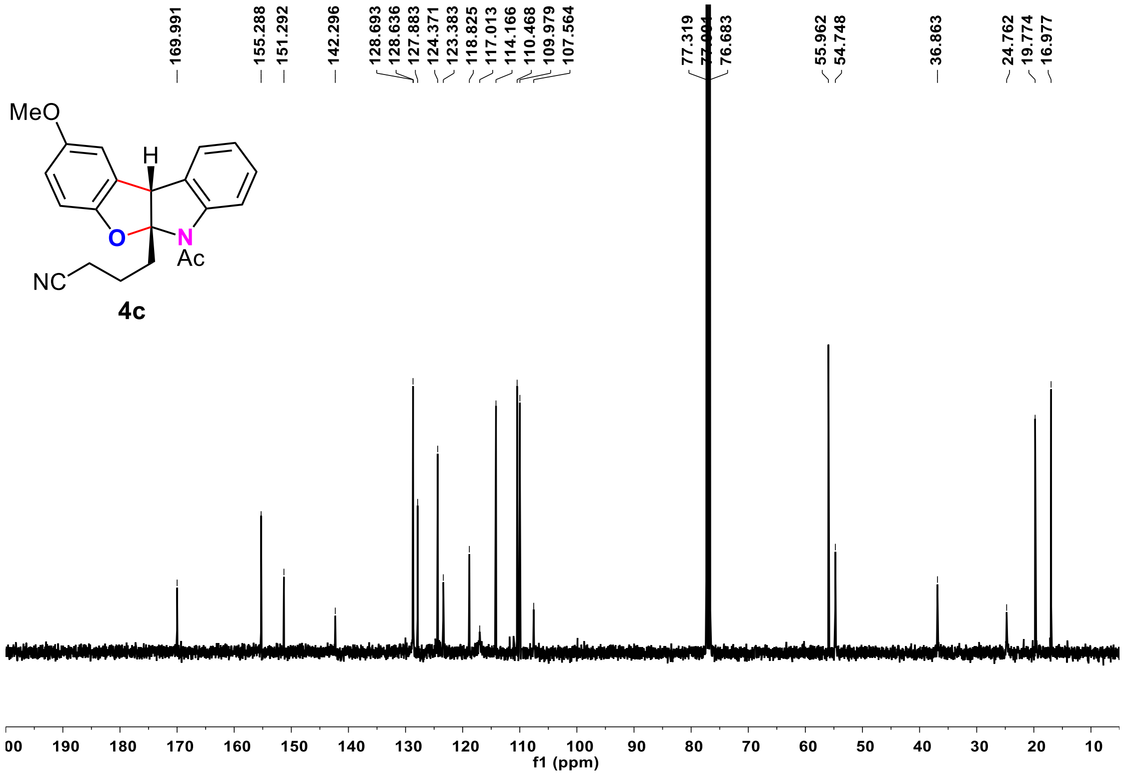

Supplementary Figure 43. ^{13}C NMR (101 MHz, $CDCl_3$) spectrum of 4c

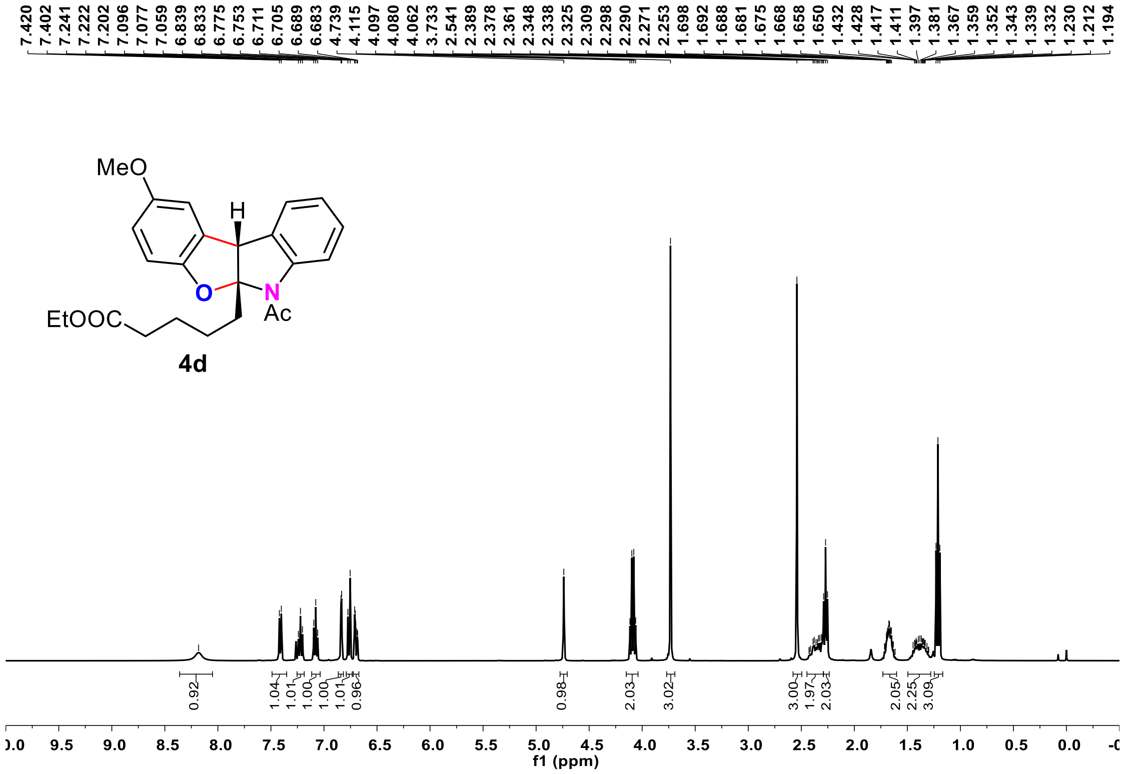

Supplementary Figure 44. **^{1}H NMR (400 MHz, $CDCl_3$) spectrum of 4d**

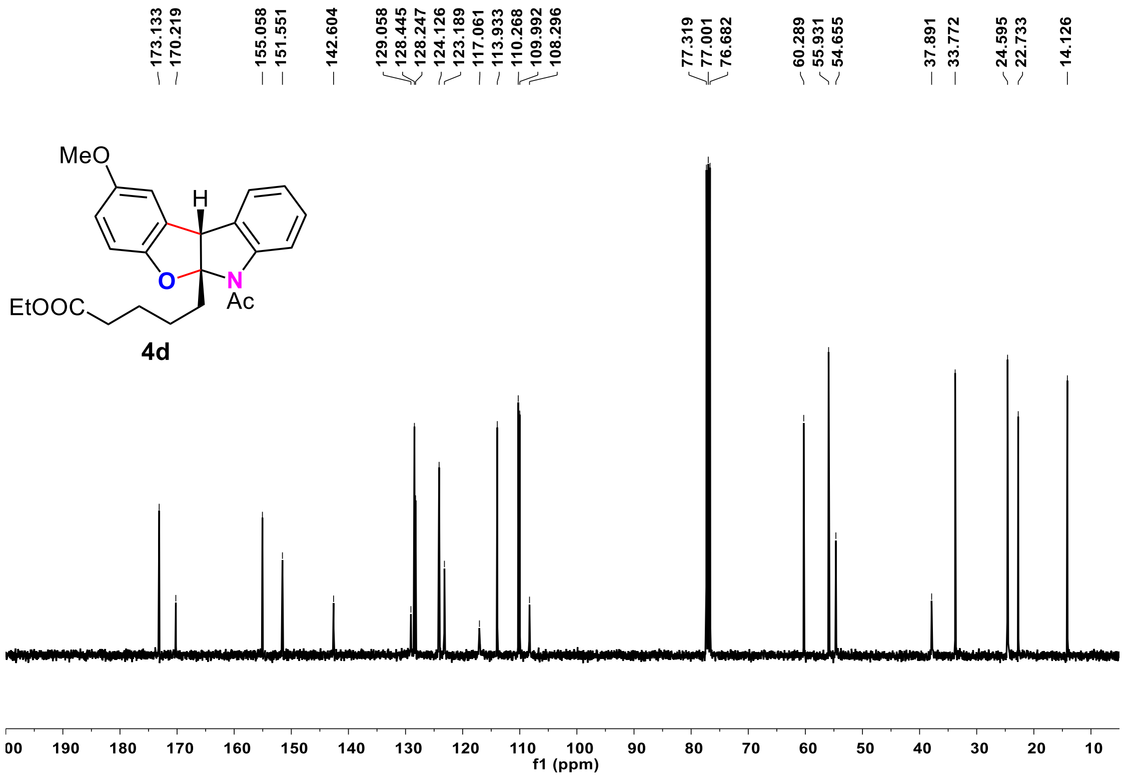

Supplementary Figure 45. **^{13}C NMR (101 MHz, $CDCl_3$) spectrum of 4d**

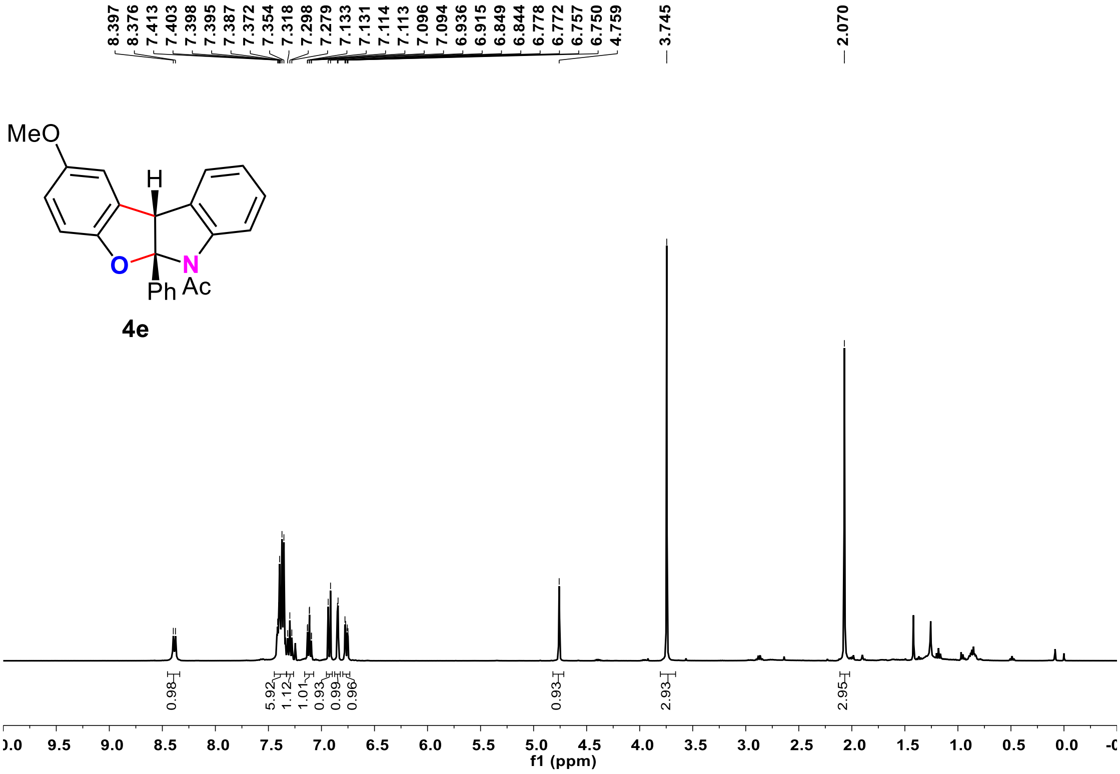

Supplementary Figure 46. ^{1}H NMR (400 MHz, $CDCl_3$) spectrum of 4e

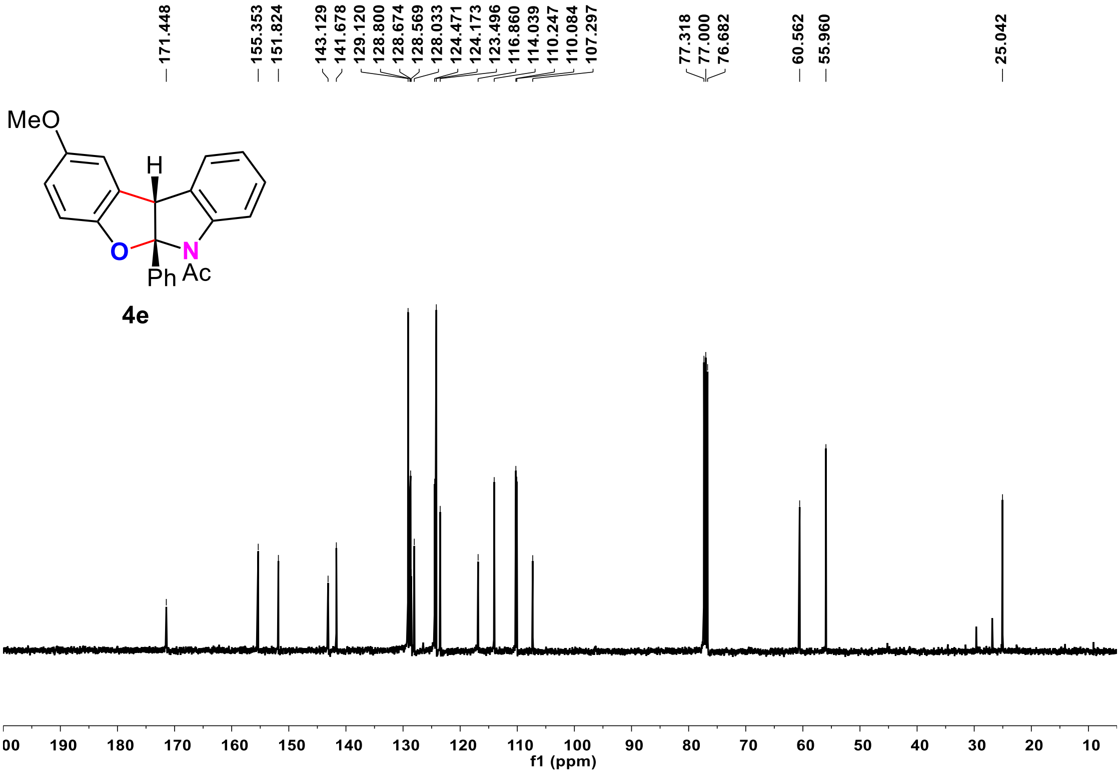

Supplementary Figure 47. ^{13}C NMR (101 MHz, $CDCl_3$) spectrum of 4e

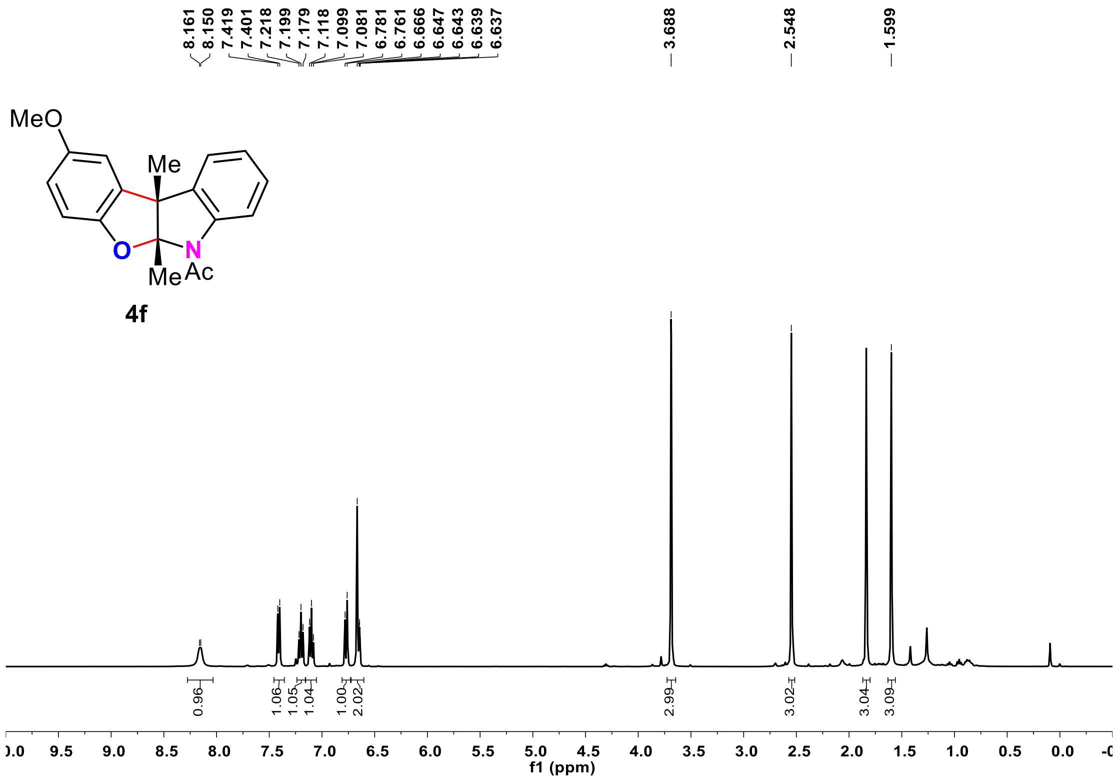

Supplementary Figure 48. ^{1}H NMR (400 MHz, $CDCl_3$) spectrum of 4f

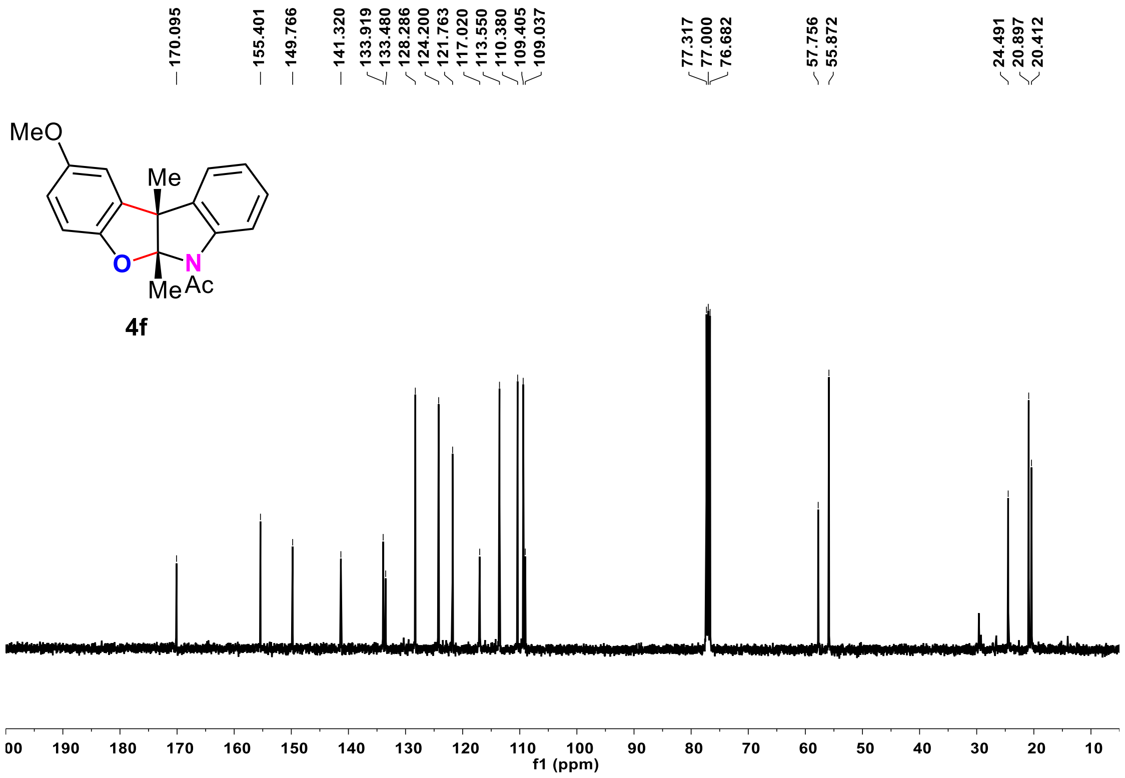

Supplementary Figure 49. ^{13}C NMR (101 MHz, $CDCl_3$) spectrum of 4f

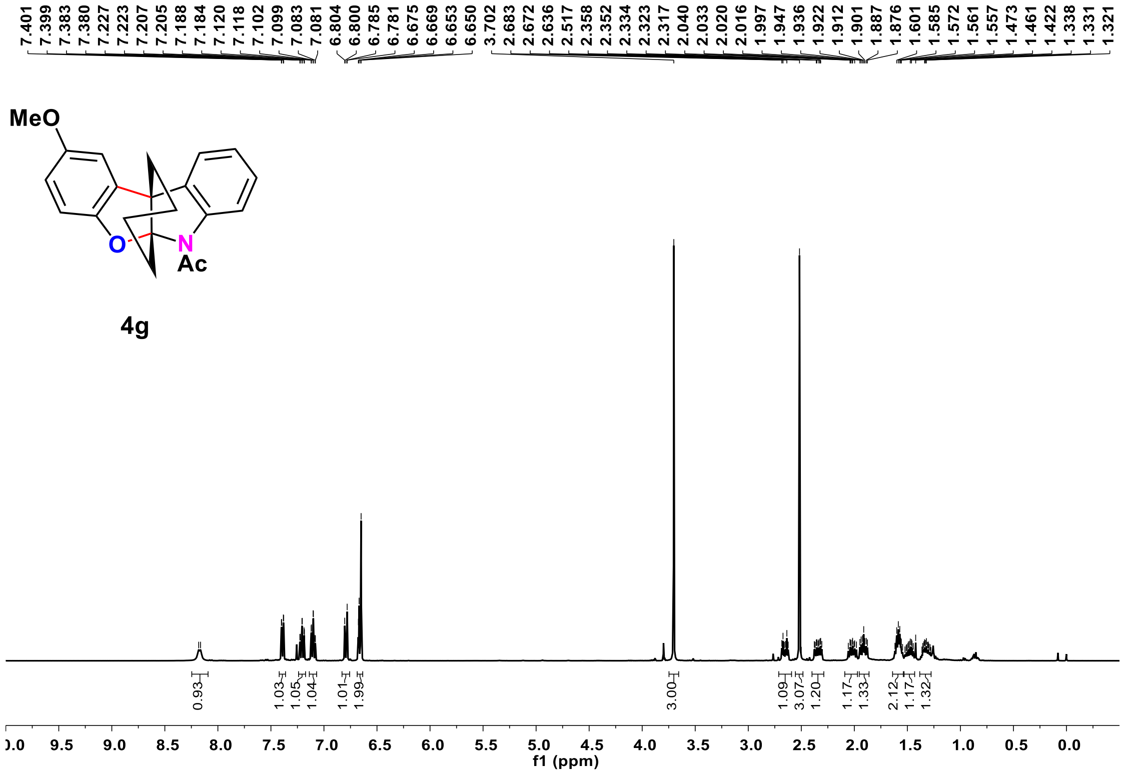

Supplementary Figure 50. ^{1}H NMR (400 MHz, $CDCl_3$) spectrum of 4g

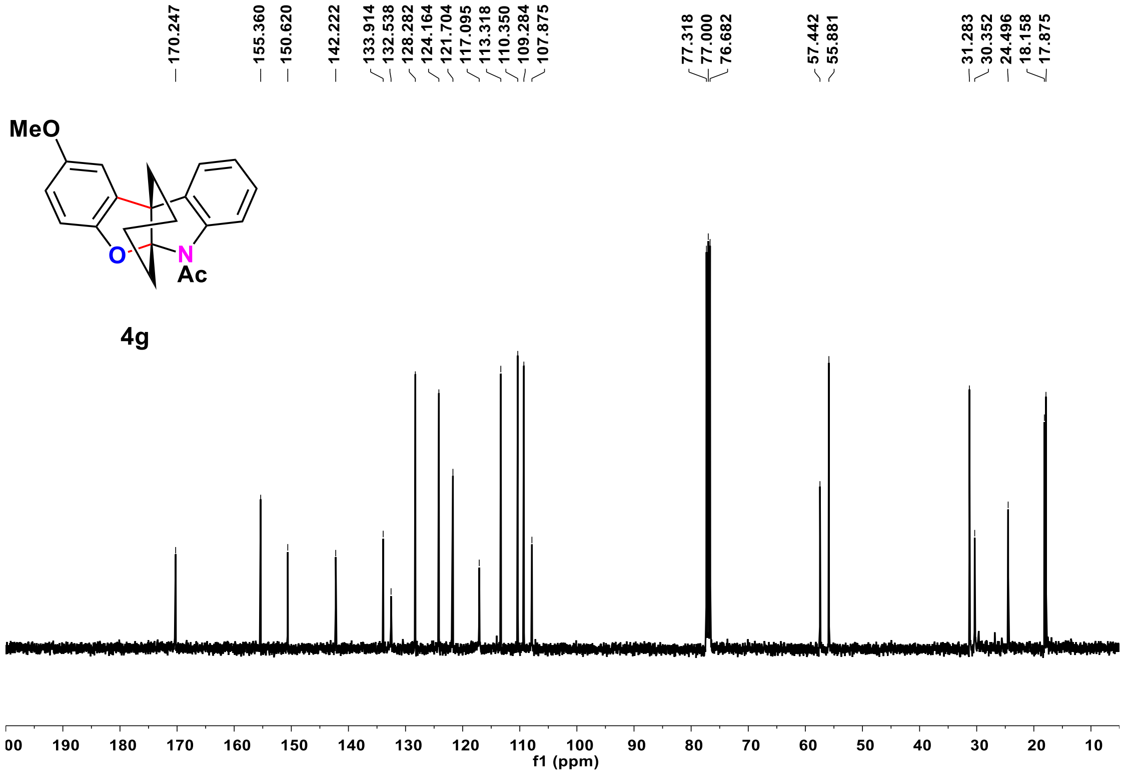

Supplementary Figure 51. ^{13}C NMR (101 MHz, $CDCl_3$) spectrum of 4g

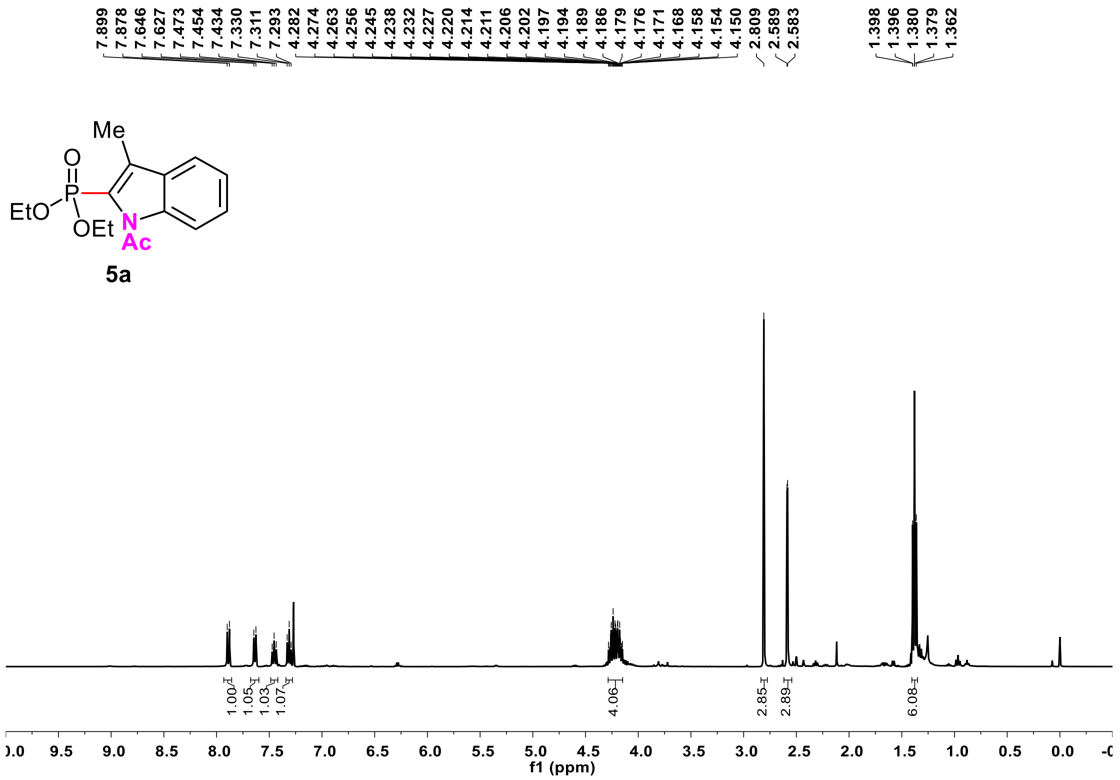

Supplementary Figure 52. ^{1}H **NMR (400 MHz,** $CDCl_3$**) spectrum of 5a**

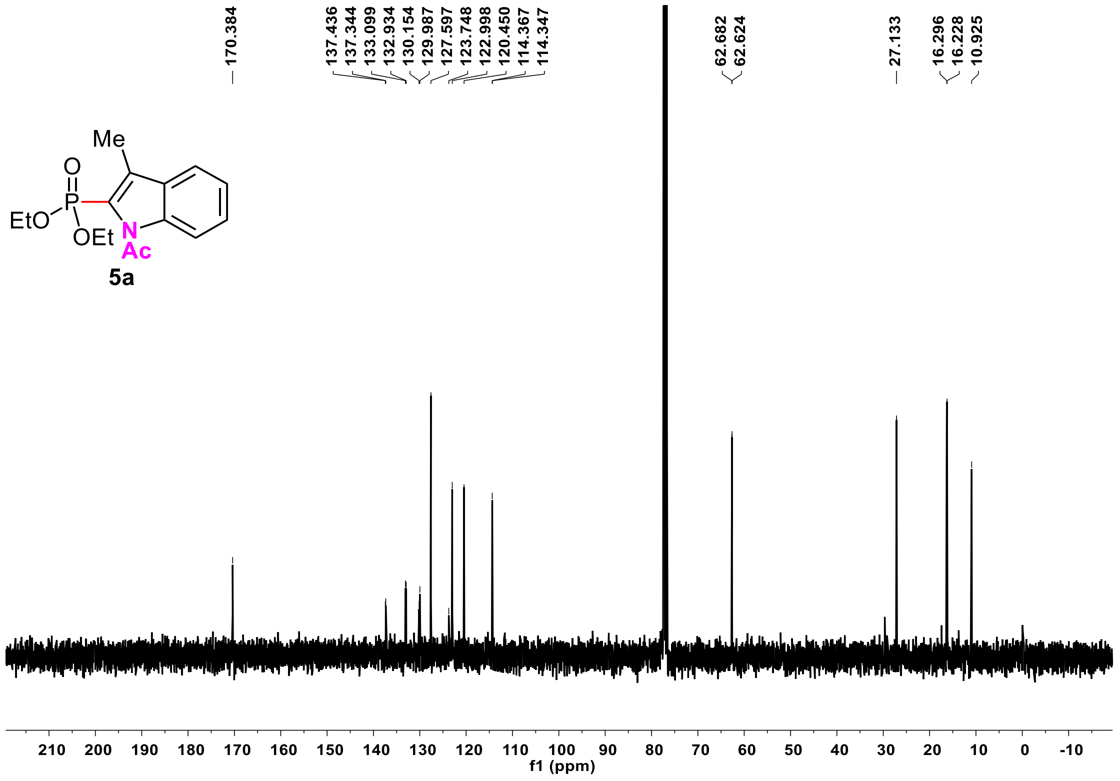

Supplementary Figure 53. ^{13}C **NMR (101 MHz,** $CDCl_3$**) spectrum of 5a**

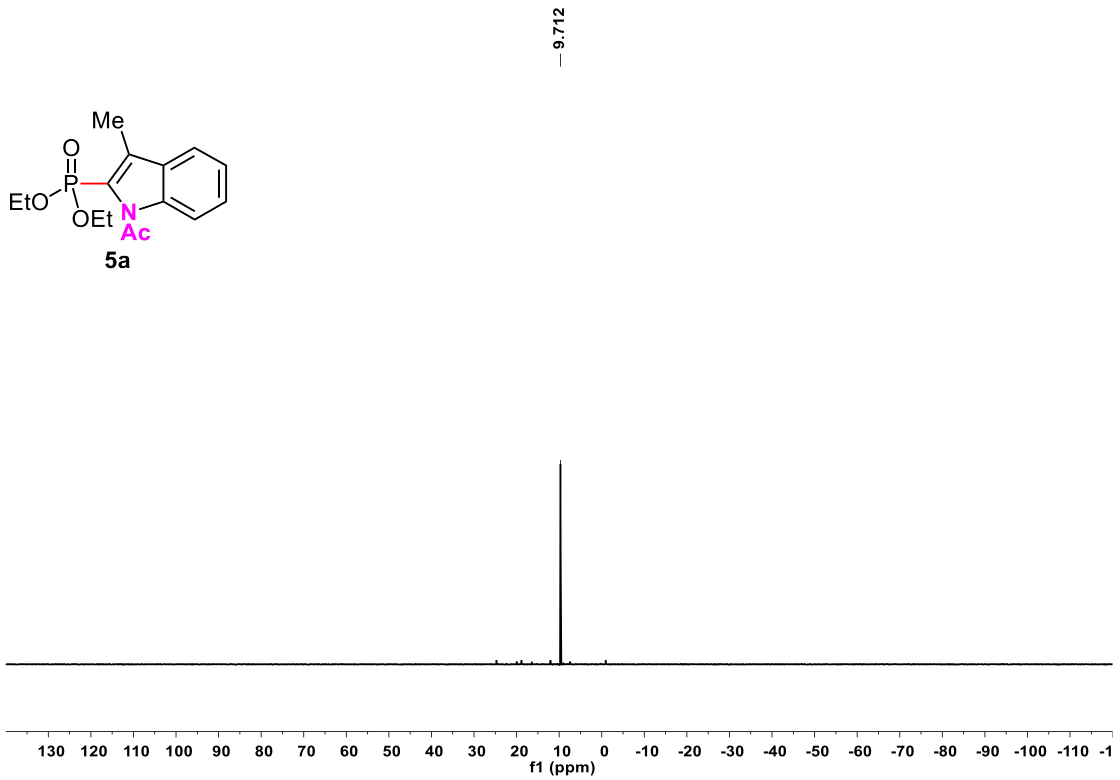

Supplementary Figure 54. ^{31}P NMR (162 MHz, $CDCl_3$) spectrum of 5a

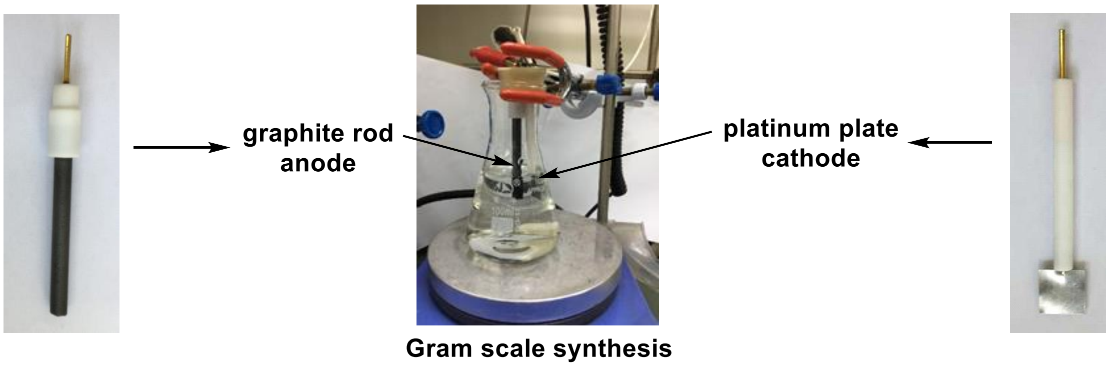

Supplementary Figure 55. Experimental setup diagram for the gram scale reaction.

Supplementary Methods

General information: All glassware was oven dried at 110 °C for hours and cooled down under vacuum. *N*-Acetyl indoles were prepared according to reported procedures.[1, 2] Unless otherwise noted, materials were obtained from commercial suppliers and used without further purification. The instrument for electrolysis was dual display potentiostat (DJS-292B) (made in China). Cyclic voltammograms were obtained on a CHI 605E potentiostat. The anodic electrode was graphite rod (ϕ 6 mm) and cathodic electrode was platinum plate (15 mm×15 mm×0.3 mm). Thin layer chromatography (TLC) employed glass 0.25 mm silica gel plates. Flash chromatography columns were packed with 200-300 mesh silica gel in petroleum (bp. 60-90 °C). Gas chromatographic analyses were performed on SHIMADZU GC-2014 gas chromatography instrument with a FID detector and biphenyl was added as internal standard. GC-MS spectra were recorded on Varian GC MS 3900-2100T or SHIMADZU GC MS-2010. High resolution mass spectra (HRMS) were measured with a Waters Micromass GCT instrument and accurate masses were reported for the molecular ion + Hydrogen $[M+H]^+$. ^{1}H and ^{13}C NMR data were recorded with Bruker Advance III (400 MHz) spectrometers with tetramethylsilane as an internal standard. All chemical shifts (δ) were reported in ppm and coupling constants (*J*) in Hz. All chemical shifts were reported relative to tetramethylsilane and d-solvent peaks (77.00 ppm, chloroform), respectively.

Reaction conditions for Substrate Scope: In an oven-dried undivided three-necked bottle (25 mL) equipped with a stir bar, phenol (0.20 mmol), *N*-acetyl indoles (0.30 mmol), nBu_4NBF_4 (65.8 mg, 0.20 mmol) and HFIP/CH_2Cl_2 (6 mL/4 mL) were combined and added. The bottle was equipped graphite rod (ϕ 6 mm, about 10 mm immersion depth in solution) as the anode and platinum plate (15 mm×15 mm×0.3 mm) as the cathode and then charged with nitrogen. The reaction mixture was stirred and electrolyzed at constant current of 10 mA ($j_{anode} \approx 16.7$ mA/cm^2) under room temperature for 1.8 h (3.4 F). When the reaction finished, the reaction mixture was washed with water and extracted with CH_2Cl_2 (10 mL x 3). The organic layers were combined, dried over Na_2SO_4, and concentrated. The pure product was obtained by flash column chromatography on silica gel (hexane: ethyl acetate = 10:1). With regard to the 2,3-disubstituted N-acetylindoles, addition of 0.4 mmol $ZnCl_2$ was needed for the synthesis of benzofuro[3, 2-b]indoline.

Procedure for gram scale synthesis of benzofuro[3, 2-b]indoline 3a: In an oven-dried conical flask (100 mL) equipped with a stir bar, 4-methoxyphenol (0.62 g, 5.0 mmol), 3-methyl-*N*-acetylindole (1.3 g, 7.5 mmol), nBu_4NBF_4 (1.3 g, 4.0 mmol) and HFIP/CH_2Cl_2 (60 mL/40 mL) were combined and added. The bottle was equipped with graphite rod (ϕ 6 mm, about 10 mm immersion depth in solution) as the anode and platinum plate (15 mm×15 mm×0.3 mm) as the cathode. The reaction mixture was stirred and electrolyzed at a constant current of 50 mA (j_{anode} ≈ 83 mA/cm^2) under air atmosphere at room temperature for 10 h (3.7 F). When the reaction finished, the reaction mixture was washed with water and extracted with CH_2Cl_2 (100 mL x 3). The organic layers were combined, dried over Na_2SO_4, and concentrated. The pure product was obtained by flash column chromatography on silica gel (hexane: ethyl acetate = 10:1). Yellow oil was obtained in 87% isolated yield (1.3 g).

General procedure for cyclic voltammetry (CV): Cyclic voltammetry was performed in a three-electrode cell connected to a schlenk line under nitrogen at room temperature. The working electrode was a steady glassy carbon disk electrode, the counter electrode a platinum wire. The reference was an Ag/AgCl electrode submerged in saturated aqueous KCl solution, and separated from reaction by a salt bridge. 6 mL of HFIP and 4 mL of CH_2Cl_2 containing 0. 2 M nBu_4NBF_4 were poured into the electrochemical cell in all cyclic voltammetry experiments. The scan rate was 0.1 V/s, ranging from 0 V to 2.0 V.

Analytical Data of Compounds

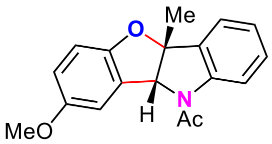

1-(8-Methoxy-4b-methyl-4b*H*-benzofuro[3,2-*b*]indol-10(9b*H*)-yl)ethanone (3a):[1] yellow oil was obtained in 99% isolated yield. Since the acetyl group could form intramolecular hydrogen bonds with the hydrogens adjacent to nitrogen atom, the spectra demonstrate a mixture of rotamers (74:26). ^{1}H NMR (400 MHz, $CDCl_3$) δ 8.16 (d, *J* = 7.6 Hz, 0.3H), 7.55-7.48 (d, *J* = 7.6 Hz, 0.7H), 7.44 (d, *J* = 6.8 Hz, 0.3H) 7.30 (t, *J* = 7.6 Hz, 1.7H), 7.12 (t, *J* = 7.3 Hz, 1.7H), 6.93 (s, 0.3H), 6.83 – 6.57 (m, 2H), 5.96 (s, 0.7H), 5.61 (s, 0.3H), 3.73 (s, 3H), 2.57(s, 0.8H), 2.49 (s, 2.2H), 1.82 (s, 3H). ^{13}C NMR (101 MHz, $CDCl_3$) δ 168.71, 167.98, 154.36, 152.97, 152.84, 141.09, 140.35, 135.01,

133.68, 129.93, 126.94, 126.02, 125.02, 124.85, 124.01, 123.44, 118.08, 116.89, 116.11, 114.47, 112.45, 111.02, 110.19, 92.79, 90.60, 72.38, 71.83, 55.99, 25.04, 24.80, 24.27.

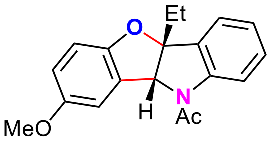

1-(4b-Ethyl-8-methoxy-4b*H*-benzofuro[3,2-*b*]indol-10(9b*H*)-yl)ethanone (3b):[1] yellow oil was obtained in 83% isolated yield. Since the acetyl group could form intramolecular hydrogen bonds with the hydrogens adjacent to nitrogen atom, the spectra demonstrate a mixture of rotamers (76:24). ^{1}H NMR (400 MHz, $CDCl_3$) δ 8.16 (d, *J* = 8.4 Hz, 0.3H), 7.49 (d, *J* = 7.6, 0.8H), 7.42 (d, *J* = 7.6 Hz, 0.3H), 7.34-7.29 (m, 0.7H), 7.29-7.26 (m, 0.9H), 7.17-7.13 (m, 1.5H), 7.11 (d, *J* = 0.8 Hz, 0.2H), 6.96 – 6.89 (m, 0.3H), 6.82 – 6.70 (m, 1.3H), 6.66 (d, *J* = 8.8 Hz, 0.7H), 6.07 (s, 0.8H), 5.68 (s, 0.2H). 3.74 (s, 3H), 2.59 (s, 0.8H), 2.50 (s, 2.3H), 2.28-2.17 (m, 1H), 2.13-2.01 (m, 1H), 0.94 (t, *J* = 7.5 Hz, 3H). ^{13}C NMR (101 MHz, $CDCl_3$) δ 168.70, 167.83, 154.28, 153.13, 140.73, 134.25, 132.64, 130.03, 129.93, 127.09, 126.01, 125.20, 124.78, 124.00, 123.76, 118.09, 116.83, 116.07, 116.07, 114.54, 112.17, 110.97, 110.90, 110.06, 95.96, 93.86, 69.76, 69.07, 55.96, 30.80, 30.53, 24.97, 24.27, 7.85, 7.73.

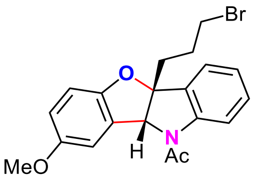

1-(4b-(3-Bromopropyl)-8-methoxy-4b*H*-benzofuro[3,2-*b*]indol-10(9b*H*)-yl)ethanone (3c):[1] brown oil was obtained in 73% isolated yield. Since the acetyl group could form intramolecular hydrogen bonds with the hydrogens adjacent to nitrogen atom, the spectra demonstrate a mixture of rotamers (77:23). ^{1}H NMR (400 MHz, $CDCl_3$) δ 8.17 (d, *J* = 8.0 Hz, 0.3H), 7.51 (d, *J* = 7.6 Hz, 0.8H) ,7.43(d, *J* = 7.2 Hz, 0.3H) 7.32 (t, *J* = 7.2 Hz, 1.2H), 7.22 – 7.09 (m, 1.8H), 6.93 (s, 0.3H), 6.89 – 6.61 (m, 2.2H), 6.08 (s, 0.8H), 5.71 (s, 0.2H), 3.74 (s, 3H), 3.43 (t, *J* = 6.1 Hz, 2H), 2.60(s, 0.7H), 2.5(s, 2.3H), 2.44 –2.30 (m, 1H), 2.30-2.13 (m, 1H), 2.12-1.98 (m, 1H), 1.94 – 1.75 (m, 1H). ^{13}C NMR (101 MHz, $CDCl_3$) δ 168.68, 154.47, 152.90, 140.57, 133.95, 130.21, 126.86, 125.28, 124.88, 124.10, 123.80, 118.25, 117.01, 116.23, 114.60, 112.51, 112.21, 110.88, 110.17, 94.76, 92.62, 70.31, 69.54, 55.99, 36.51, 33.21, 26.96, 26.77, 25.01, 24.32.

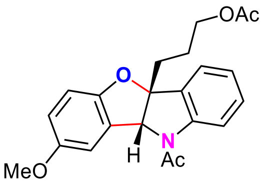

3-(10-Acetyl-8-methoxy-9b,10-dihydro-4b*H*-benzofuro[3,2-*b*]indol-4b-yl)propyl acetate (3d):[1] yellow oil was obtained in 78% isolated yield. Since the acetyl group could form intramolecular hydrogen bonds with the hydrogens adjacent to nitrogen atom, the spectra demonstrate a mixture of rotamers (78:22). ^{1}H NMR (400 MHz, $CDCl_3$) δ 8.17 (d, *J* = 8.4 Hz, 0.2H), 7.51 (d, *J* = 7.2 Hz, 0.8H), 7.42 (d, *J* = 7.6 Hz, 0.2H), 7.37-7.29 (m, 1.1H), 7.27 (s, 0.6H), 7.19 – 7.15 (m, 1.1H), 7.14 (s, 0.5H), 7.12(s, 0.2H), 6.93 (s, 0.2H), 6.82 – 6.69 (m, 1.3H), 6.66 (d, *J* = 8.4 Hz, 0.8H), 6.09 (s, 0.8H), 5.69 (s, 0.2H), 4.09 (t, *J* = 6.8 Hz, 2H), 3.74 (s, 3H), 2.60 (s, 0.7H), 2.51 (s, 2.3H), 2.34-2.18 (m, 1H), 2.17 – 2.06 (m, 1H), 2.03 (s, 3H), 1.90 – 1.75 (m,1H), 1.70-1.55 (m, 1H). ^{13}C NMR (101 MHz, $CDCl_3$) δ 171.02, 168.68, 167.82, 154.41, 152.92, 141.43, 140.57, 134.01, 130.25, 130.15, 126.88, 125.82, 125.23, 124.84, 124.08, 123.80, 118.18, 116.95, 116.16, 114.59, 112.15, 111.06, 110.86, 110.14, 94.90, 92.76, 70.24, 69.45, 63.93, 55.96, 34.37, 25.00, 24.31, 22.97, 22.87, 20.90.

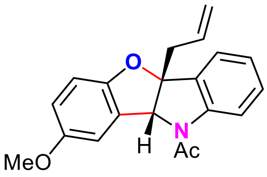

1-(4b-Allyl-8-methoxy-4b*H*-benzofuro[3,2-*b*]indol-10(9b*H*)-yl)ethanone (3e):[1] yellow oil was obtained in 72% isolated yield. Since the acetyl group could form intramolecular hydrogen bonds with the hydrogens adjacent to nitrogen atom, the spectra demonstrate a mixture of rotamers (71:29). ^{1}H NMR (400 MHz, $CDCl_3$) δ 8.15 (d, *J* = 8.0 Hz, 0.3H), 7.53 (d, *J* = 7.6 Hz, 0.7H), 7.45 (d, *J* = 7.2 Hz, 0.3H), 7.36 – 7.29 (m, 1H), 7.27 (d, *J* = 2.4 Hz, 0.6H), 7.14 (t, *J* = 7.6 Hz, 1.6H), 6.92 (s, 0.3H), 6.82-6.70 (m, 1.3H), 6.66 (d, *J* = 8.7 Hz, 0.7H), 6.06 (s, 0.7H), 5.89 – 5.51 (m, 1.3H), 5.37 – 4.92 (m, 2H), 3.74 (s, 3H), 2.96 (dd, *J* = 14.0, 6.8 Hz, 1H), 2.79 (dd, *J* = 14.0, 7.2 Hz, 1H), 2.55(s, 0.9H), 2.50(s, 2.1H). ^{13}C NMR (101 MHz, $CDCl_3$) δ 168.63, 154.41, 152.93, 140.84, 133.85, 131.54, 131.01, 130.21, 130.09, 126.96, 125.34, 124.80, 123.94, 123.76, 120.16, 119.96, 118.16, 116.89, 116.11, 114.53, 112.28, 111.01, 110.93, 110.10, 103.45, 94.74, 92.55, 69.55, 69.26, 55.98, 42.41, 42.11, 25.01, 24.26.

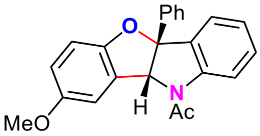

1-(8-Methoxy-4b-phenyl-4b*H*-benzofuro[3,2-*b*]indol-10(9b*H*)-yl)ethanone (3f):[1] brown oil was obtained in 95% isolated yield. Since the acetyl group could form intramolecular hydrogen bonds with the hydrogens adjacent to nitrogen atom, the spectra demonstrate a mixture of rotamers (70:30). ^{1}H NMR (400 MHz, $CDCl_3$) δ 8.25 (d, *J* = 8.0 Hz, 0.3H), 7.40-7.27 (m, 7.7H), 7.24 (d, *J* = 8.4 Hz, 0.7H), 7.14 (t, *J* = 7.2 Hz, 1H), 6.95 (s, 0.3H), 6.92 – 6.77 (m, 2H), 6.18 (s, 0.7H), 5.79 (s, 0.3H), 3.76 (s, 3H), 2.56 (s, 2H), 2.52 (s, 1H). ^{13}C NMR (101 MHz, $CDCl_3$) δ 168.68, 154.70, 153.21, 143.82, 141.73, 141.51, 141.27, 134.58, 130.29, 130.20, 128.74, 128.56, 128.27, 128.09, 127.07, 126.55, 125.38, 125.27, 124.32, 118.22, 117.05, 116.25, 114.55, 112.33, 111.22, 110.95, 110.24, 99.92, 94.00, 75.10, 74.42, 56.02, 25.08, 24.30.

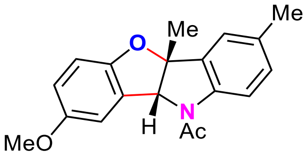

1-(8-Methoxy-3,4b-dimethyl-4b*H*-benzofuro[3,2-*b*]indol-10(9b*H*)-yl)ethanone (3g):[1] yellow oil was obtained in 70% isolated yield. Since the acetyl group could form intramolecular hydrogen bonds with the hydrogens adjacent to nitrogen atom, the spectra demonstrate a mixture of rotamers (76:24). ^{1}H NMR (400 MHz, $CDCl_3$) δ 8.03 (d, *J* = 8.0 Hz, 0.3H), 7.42 – 7.27 (m, 1.6H), 7.24 (s, 0.3H), 7.10 (d, *J* = 8.0 Hz, 1.1H), 7.03 (d, *J* = 8.4 Hz, 0.7H), 6.93 (s, 0.3H), 6.82 – 6.69 (m, 1.3H), 6.65 (d, *J* = 8.8 Hz, 0.7H), 5.96 (s, 0.8H), 5.59 (s, 0.2H), 3.74 (s, 3H), 2.57(s, 0.8H), 2.48(s, 2.3H), 2.35(s, 2.1H), 2.33(s, 0.9H), 1.84-1.78 (m, 3H). ^{13}C NMR (101 MHz, $CDCl_3$) δ 168.54, 154.35, 152.94, 152.81, 138.87, 138.10, 135.04, 134.65, 133.86, 130.67, 130.54, 127.02, 125.38, 123.77, 117.81, 116.89, 116.11, 114.29, 112.43, 111.02, 110.18, 92.84, 90.67, 72.55, 71.95, 56.00, 25.05, 24.95, 24.77, 24.20, 21.01, 20.77.

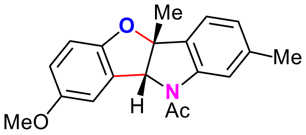

1-(8-Methoxy-2,4b-dimethyl-4b*H*-benzofuro[3,2-*b*]indol-10(9b*H*)-yl)ethanone (3h): yellow oil was obtained in 80% isolated yield. Since the acetyl group could form intramolecular hydrogen bonds with the hydrogens adjacent to nitrogen atom, the spectra demonstrate a mixture of rotamers

(73:27). ^{1}H NMR (400 MHz, $CDCl_3$) δ 8.01 (s, 0.3H), 7.40 (d, J = 7.6 Hz, 0.8H), 7.32 (d, J = 6.8 Hz, 0.3H), 7.26 (d, J = 0.8 Hz, 0.3 H),7.02-6.87 (m, 2H), 6.82-6.68 (m, 1.3H), 6.64 (d, J = 8.4 Hz, 0.8H), 5.96 (s, 0.7H), 5.60 (s, 0.3H), 3.74 (s, 3H), 2.58 (s, 0.8H), 2.51 (s, 2.2H), 2.36 (s, 2.2H), 2.34 (s, 0.8H), 1.81 (s, 3H). ^{13}C NMR (101 MHz, $CDCl_3$) δ 168.70, 167.92, 154.32, 152.96, 152.84, 140.57, 140.30, 140.18, 132.33, 127.03, 126.13, 125.72, 124.85, 124.62, 123.08, 118.55, 116.87, 116.10, 115.22, 112.40, 111.02, 110.19, 92.68, 90.49, 72.67, 72.11, 56.01, 25.11, 24.80, 24.36, 21.97, 21.73. HRMS (ESI) calculated for $C_{19}H_{19}NO_3$ $[M+H]^+$: 310.1438; found: 310.1433.

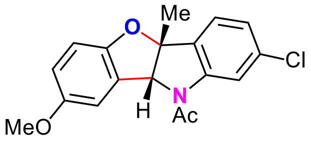

1-(2-Chloro-8-methoxy-4b-methyl-4b*H*-benzofuro[3,2-*b*]indol-10(9b*H*)-yl)ethanone (3i):[1] brown oil was obtained in 73% isolated yield. Since the acetyl group could form intramolecular hydrogen bonds with the hydrogens adjacent to nitrogen atom, the spectra demonstrate a mixture of rotamers (66:34). ^{1}H NMR (400 MHz, $CDCl_3$) δ 8.21 (s, 0.3H), 7.43 (d, J = 8.0 Hz, 0.7H), 7.35 (d, J = 8.0 Hz, 0.4H), 7.31-7.21 (m, 0.9H), 7.17 – 7.02 (m, 1.6H), 6.92 (s, 0.4H), 6.83 – 6.59 (m, 2.0H), 5.97 (s, 0.7H), 5.63 (s, 0.3H), 3.75 (s, 3H), 2.58(s, 1H), 2.51(s, 2H), 1.81 (s, 3H). ^{13}C NMR (101 MHz, $CDCl_3$) δ 168.57, 168.09, 154.51, 152.82, 152.72, 142.06, 141.47, 135.71, 133.66, 126.57, 125.92, 125.87, 125.02, 124.29, 124.09, 118.37, 117.09, 116.27, 114.90, 112.39, 111.14, 110.98, 110.30, 92.24, 89.95, 72.80, 72.39, 56.03, 25.10, 25.02, 24.74, 24.23.

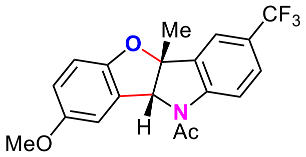

1-(8-Methoxy-4b-methyl-3-(trifluoromethyl)-4b*H*-benzofuro[3,2-*b*]indol-10(9b*H*)-yl)ethanone (3j): white solid was obtained in 47% isolated yield. Since the acetyl group could form intramolecular hydrogen bonds with the hydrogens adjacent to nitrogen atom, the spectra demonstrate a mixture of rotamers (66:34). ^{1}H NMR (400 MHz, $CDCl_3$) δ 8.27 (d, J = 8.5 Hz, 0.4H), 7.76 (s, 0.7H), 7.73 – 7.66 (m, 0.4H), 7.57 (d, J = 8.5 Hz, 1.2H), 7.26 – 7.19 (m, 0.8H), 6.93 (s, 0.4H), 6.86 – 6.61 (m, 2.1H), 6.01 (s, 0.6H), 5.68 (s, 0.4H), 3.75 (s, 3H), 2.61 (s, 1H), 2.53 (s, 2H), 1.85 (s, 3H). ^{13}C NMR (101 MHz, $CDCl_3$) δ 168.74, 168.44, 154.55, 152.73, 143.79, 143.12, 136.81, 135.81, 134.45, 127.49, 126.35, 125.96, 125.33 (q, $J_{C\text{-}F}$ = 23.8 Hz), 122.47, 120.97, 118.13, 117.08,

116.28, 114.31, 112.47, 111.14 (q, J_{C-F} = 20.0 Hz), 110.38, 92.16, 89.87, 72.79, 72.43, 56.01, 25.19, 24.99, 24.75, 24.31. ^{19}F NMR (377 MHz, $CDCl_3$) δ -61.76, -61.80. HRMS (ESI) calculated for $C_{19}H_{16}F_3NO_3$ $[M+H]^+$: 364.1155; found: 364.1155.

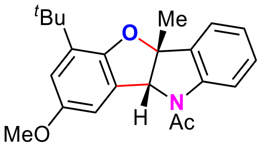

1-(6-(*tert*-Butyl)-8-methoxy-4b-methyl-4b*H*-benzofuro[3,2-*b*]indol-10(9b*H*)-yl)ethanone (3k): yellow oil was obtained in 85% isolated yield. Since the acetyl group could form intramolecular hydrogen bonds with the hydrogens adjacent to nitrogen atom, the spectra demonstrate a mixture of rotamers (69:31). ^{1}H NMR (400 MHz, $CDCl_3$) δ 8.14 (d, *J* = 8.2 Hz, 0.3H), 7.53 (d, *J* = 7.2z, 0.7H), 7.44 (d, *J* = 7.6 Hz, 0.3H), 7.28 (t, *J* = 7.6 Hz, 1H), 7.20-7.06 (m, 2.4H), 6.80-6.67 (m, 1.3H), 5.99 (s, 0.7H), 5.58 (s, 0.3H), 3.74 (s, 3H), 2.58 (s, 0.9H), 2.50 (s, 2.1H), 1.84 (s, 0.9H), 1.82 (s, 2.1H), 1.31 (s, 2.8H), 1.28 (s, 6.3H). ^{13}C NMR (101 MHz, $CDCl_3$) δ 168.82, 154.21, 154.08, 151.08, 150.95, 140.16, 135.93, 134.20, 129.79, 129.68, 127.00, 126.01, 124.92, 124.03, 123.34, 118.12, 114.93, 114.66, 114.48, 108.32, 106.75, 89.66, 72.32, 71.74, 55.90, 34.35, 34.22, 28.96, 25.16, 25.01. HRMS (ESI) calculated for $C_{22}H_{25}NO_3$ $[M+H]^+$: 352.1907; found: 352.1901.

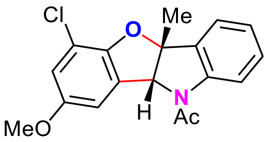

1-(6-Chloro-8-methoxy-4b-methyl-4b*H*-benzofuro[3,2-*b*]indol-10(9b*H*)-yl)ethanone (3l): yellow oil was obtained in 92% isolated yield. Since the acetyl group could form intramolecular hydrogen bonds with the hydrogens adjacent to nitrogen atom, the spectra demonstrate a mixture of rotamers (81:19). ^{1}H NMR (400 MHz, $CDCl_3$) δ 8.15 (d, *J* = 6.8 Hz, 0.2H), 7.60 (d, *J* = 7.2 Hz, 0.9H), 7.51(d, *J* = 5.6 Hz, 0.2H), 7.33 (t, *J* = 7.7 Hz, 1.1H), 7.21(s, 0.9H), 7.19-7.10(m, 1.8H), 6.84(s, 0.2H), 6.77(s, 1H), 5.99 (s, 0.8H), 5.63 (s, 0.2H), 3.73 (s, 3H), 2.56(s, 0.7H), 2.51(s, 2.4H), 1.88 (s, 3H). ^{13}C NMR (101 MHz, $CDCl_3$) δ 168.69, 154.63, 149.17, 140.26, 134.45, 130.18, 128.30, 125.36, 125.06, 124.20, 116.68, 116.02, 115.00, 114.38, 111.63, 110.05, 91.48, 72.63, 72.08, 56.15, 24.98, 24.84. HRMS (ESI) calculated for $C_{18}H_{16}ClNO_3$ $[M+H]^+$: 330.0891; found: 330.0887.

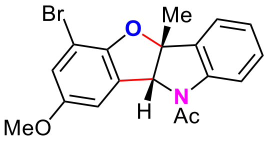

1-(6-Bromo-8-methoxy-4b-methyl-4b*H*-benzofuro[3,2-*b*]indol-10(9b*H*)-yl)ethanone (3m): yellow oil was obtained in 95% isolated yield. Since the acetyl group could form intramolecular hydrogen bonds with the hydrogens adjacent to nitrogen atom, the spectra demonstrate a mixture of rotamers (80:20). ^{1}H NMR (400 MHz, $CDCl_3$) δ 8.15 (d, *J* = 7.2 Hz, 0.2H), 7.59 (d, *J* = 7.6 Hz, 0.8H), 7.51(d, *J* = 6.4 Hz, 0.2H), 7.32 (t, *J* = 7.7 Hz, 1H), 7.25 (d, *J* = 2.0 Hz, 0.7H), 7.19 – 7.08 (m, 1.9H), 6.96-6.90 (m, 1H), 6.88 (s, 0.2H), 6.01 (s, 0.8H), 5.65 (s, 0.2H), 3.72 (s, 3H), 2.56 (s, 0.6H), 2.50 (s, 2.4H), 1.87 (s, 3H). ^{13}C NMR (101 MHz, $CDCl_3$) δ 168.67, 167.77, 154.75, 150.55, 140.95, 140.23, 134.49, 133.24, 130.14, 127.91, 127.06, 125.35, 125.06, 124.18, 123.72, 119.28, 118.61, 118.00, 114.35, 112.37, 110.80, 103.49, 102.39, 93.37, 91.19, 72.85, 72.31, 56.17, 24.95, 24.85, 24.18. HRMS (ESI) calculated for $C_{18}H_{16}BrNO_3$ $[M+H]^+$: 374.0386; found: 374.0385.

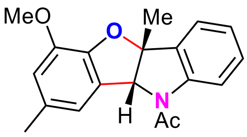

1-(6-Methoxy-4b,8-dimethyl-4b*H*-benzofuro[3,2-*b*]indol-10(9b*H*)-yl)ethanone (3n): yellow oil was obtained in 68% isolated yield. Since the acetyl group could form intramolecular hydrogen bonds with the hydrogens adjacent to nitrogen atom, the spectra demonstrate a mixture of rotamers (67:33). ^{1}H NMR (400 MHz, $CDCl_3$) δ 8.16 (d, *J* = 8.2 Hz, 0.3H), 7.61 (d, *J* = 7.5 Hz, 0.7H), 7.58 – 7.48 (m, 0.3H), 7.30 (t, *J* = 7.7 Hz, 1H), 7.20 – 7.08 (m, 2.3H), 6.79 (s, 0.3H), 6.65 – 6.55 (m, 1H), 5.98 (s, 0.7H), 5.63 (s, 0.3H), 3.81 (s, 3H), 2.59 (s, 1H), 2.51 (s, 2H), 2.28 (s, 3H), 1.88 (d, *J* = 6.7 Hz, 3H). ^{13}C NMR (101 MHz, $CDCl_3$) δ 168.64, 145.30, 144.52, 143.88, 141.06, 140.34, 134.92, 131.76, 131.48, 130.02, 129.93, 127.23, 126.02, 125.39, 124.83, 124.01, 123.75, 119.75, 117.99, 116.74, 114.43, 113.80, 113.23, 93.25, 91.14, 72.64, 71.89, 55.74, 25.18, 25.10, 24.92, 24.35, 21.32, 21.16. HRMS (ESI) calculated for $C_{19}H_{19}NO_3$ $[M+H]^+$: 310.1438; found: 310.1433.

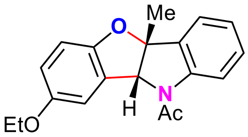

1-(8-Ethoxy-4b-methyl-4b*H*-benzofuro[3,2-*b*]indol-10(9b*H*)-yl)ethanone (3o):[1] brown oil was obtained in 80% isolated yield. Since the acetyl group could form intramolecular hydrogen bonds with the hydrogens adjacent to nitrogen atom, the spectra demonstrate a mixture of rotamers (73:27). ^{1}H NMR (400 MHz, $CDCl_3$) δ 8.16 (d, *J* = 8.0 Hz, 0.3H), 7.52 (d, *J* = 7.6 Hz, 0.7H), 7.44 (d, *J* = 7.6 Hz,

0.3H), 7.34 – 7.27 (m, 1.7H), 7.13 (t, J = 7.6 Hz, 1.7H), 6.94 (s, 0.3H), 6.74 (dd, J = 8.4, 2.8 Hz, 1H), 6.70 (d, J = 8.8 Hz, 0.3H), 6.64 (d, J = 8.8 Hz, 0.7H), 5.96 (s, 0.7H), 5.61 (s, 0.3H), 3.95 (q, J = 6.9 Hz, 2H), 2.58 (s, 0.8H), 2.50 (s, 2.2H), 1.83 (s, 3H), 1.35 (t, J = 6.9 Hz, 3H). ^{13}C NMR (101 MHz, $CDCl_3$) δ 168.68, 167.97, 153.65, 152.87, 152.75, 141.07, 140.33, 135.00, 129.91, 126.90, 125.99, 125.01, 124.83, 123.99, 123.44, 118.06, 117.48, 116.70, 114.45, 113.33, 111.85, 110.94, 110.12, 92.74, 90.55, 72.37, 71.80, 64.44, 64.28, 25.06, 24.80, 24.30, 14.86.

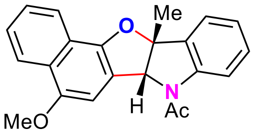

1-(5-Methoxy-11b-methyl-6b*H*-naphtho[2',1':4,5]furo[3,2-*b*]indol-7(11b*H*)-yl)ethanone (3p):[1] black solid was obtained in 35% isolated yield. Since the acetyl group could form intramolecular hydrogen bonds with the hydrogens adjacent to nitrogen atom, the spectra demonstrate a mixture of rotamers (77:23). ^{1}H NMR (400 MHz, $CDCl_3$) δ 8.25-8.10 (m, 1.2H), 8.00 – 7.93 (d, J = 5.2 Hz, 0.2H), 7.93-7.86 (m, 0.8H), 7.62 (d, J = 7.6 Hz, 0.8H), 7.53 (d, J = 7.6 Hz, 0.3H), 7.52-7.39 (m, 2H), 7.30 (t, J = 8.0 Hz, 1.1H), 7.21-7.09 (m, 2.5H), 6.75 (s, 0.2H), 6.17 (s, 0.8H), 5.83 (s, 0.2H), 3.96 (s, 3H), 2.67 (s, 0.6H), 2.54 (s, 2.3H), 1.95 (s, 0.7H), 1.93 (s, 2.3H). ^{13}C NMR (101 MHz, $CDCl_3$) δ 168.93, 158.45, 151.30, 150.20, 140.40, 135.49, 134.77, 130.01, 129.90, 126.59, 125.96, 125.90, 125.23, 124.00, 122.47, 121.73, 120.85, 118.27, 114.65, 102.66, 90.78, 73.42, 55.97, 25.13, 24.98.

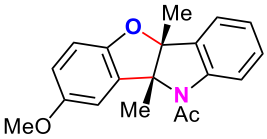

1-(8-Methoxy-4b,9b-dimethyl-4b*H*-benzofuro[3,2-*b*]indol-10(9b*H*)-yl)ethanone (3q): white solid was obtained in 26% isolated yield (additive: 2 equiv of $ZnCl_2$). ^{1}H NMR (400 MHz, $CDCl_3$) δ 7.55 (d, J = 7.5 Hz, 1H), 7.49 (s, 1H), 7.35 (t, J = 8.0 Hz, 1H), 7.21 – 7.11 (m, 2H), 6.78-6.72 (m, 1H), 6.64 (d, J = 8.8 Hz, 1H), 3.80 (s, 3H), 2.40 (s, 3H), 1.86 (s, 3H), 1.66 (s, 3H). ^{13}C NMR (101 MHz, $CDCl_3$) δ 168.08, 153.59, 153.06, 141.67, 132.10, 130.38, 129.06, 124.93, 123.54, 116.14, 114.60, 114.27, 109.80, 95.32, 78.81, 56.00, 26.66, 19.09, 18.40. HRMS (ESI) calculated for $C_{19}H_{19}NO_3$ $[M+H]^+$: 310.1438; found: 310.1428.

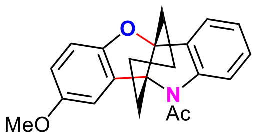

1-(8-Methoxy-10H-4b,9b-butanobenzofuro[3,2-*b*]indol-10-yl)ethanone (3r): brown oil was obtained in 25% isolated yield (additive: 2 equiv of $ZnCl_2$). ^{1}H NMR (400 MHz, $CDCl_3$) δ 7.54 (d, *J* = 7.2 Hz, 1H), 7.45 (s, 1H), 7.41-7.32 (m, 1H), 7.24-7.08 (m, 2H), 6.76 (dd, *J* = 8.7, 2.8 Hz, 1H), 6.67 (d, *J* = 8.4 Hz, 1H), 3.80 (s, 3H), 3.16 (d, *J* = 14.4 Hz, 1H), 2.67 – 2.44 (m, 1H), 2.38 (s, 3H), 1.96 – 1.75 (m, 2H), 1.70 - 1.47 (m, 2H), 1.25 – 1.04 (m, 2H). ^{13}C NMR (101 MHz, $CDCl_3$) δ 168.06, 154.06, 153.64, 142.46, 130.80, 130.50, 128.05, 124.62, 123.46, 115.91, 114.46, 114.02, 110.01, 94.10, 78.28, 77.20, 55.96, 29.58, 26.35, 19.41, 19.14. HRMS (ESI) calculated for $C_{21}H_{21}NO_3$ $[M+H]^+$: 336.1594; found: 336.1582.

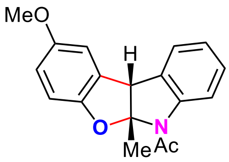

1-(2-Methoxy-5a-methyl-5a,10b-dihydro-6H-benzofuro[2,3-b]indol-6-yl)ethan-1-one (4a): colorless oil was obtained in 84% isolated yield. ^{1}H NMR (400 MHz, $CDCl_3$) δ 8.20 (s, 1H), 7.43 (d, *J* = 7.2 Hz, 1H), 7.32 – 7.18 (m, 1H), 7.09 (td, *J* = 7.5, 1.1 Hz, 1H), 6.83 (d, *J* = 2.0 Hz, 1H), 6.77 (d, *J* = 8.8 Hz, 1H), 6.70 (dd, *J* = 8.8, 2.4 Hz, 1H), 4.63 (s, 1H), 3.73 (s, 3H), 2.56 (s, 3H), 2.01 (s, 3H). ^{13}C NMR (101 MHz, $CDCl_3$) δ 170.26, 155.12, 151.47, 142.10, 128.77, 128.52, 128.27, 124.17, 123.46, 117.25, 113.96, 110.35, 110.07, 106.11, 57.42, 55.96, 25.74, 24.59. HRMS (ESI) calculated for $C_{18}H_{17}NO_3$ $[M+H]^+$: 296.1281; found: 296.1272.

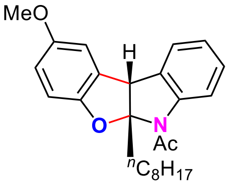

1-(2-Methoxy-5a-octyl-5aH-benzofuro[2,3-b]indol-6(10bH)-yl)ethanone (4b): colorless oil was obtained in 86% isolated yield. ^{1}H NMR (400 MHz, $CDCl_3$) δ 8.20 (s, 1H), 7.41 (d, *J* = 7.2 Hz, 1H), 7.25-7.19 (m, 1H), 7.13-7.01 (m, 1H), 6.84 (d, *J* = 2.4 Hz, 1H), 6.76 (d, *J* = 8.4 Hz, 1H), 6.70 (dd, *J* = 8.8, 2.8 Hz, 1H), 4.74 (s, 1H), 3.73 (s, 3H), 2.54 (s, 3H), 2.43-2.31 (m, 1H), 2.30 – 2.19 (m, 1H), 1.45 – 1.10 (m, 12H), 0.86 (t, *J* = 6.4 Hz, 3H). ^{13}C NMR (101 MHz, $CDCl_3$) δ 170.33, 155.01, 151.64, 142.74, 129.21, 128.41, 128.38, 124.07, 123.14, 117.09, 113.88, 110.23, 110.03, 108.62,

55.94, 54.72, 38.28, 31.69, 29.36, 29.24, 29.10, 24.63, 23.21, 22.54, 14.04. HRMS (ESI) calculated for $C_{25}H_{31}NO_3$ $[M+H]^+$: 394.2377; found: 394.2370.

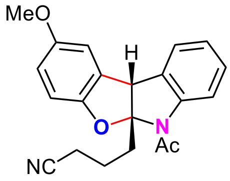

4-(6-Acetyl-2-methoxy-6,10b-dihydro-5aH-benzofuro[2,3-b]indol-5a-yl)butanenitrile (4c): colorless oil was obtained in 79% isolated yield. ^1H NMR (400 MHz, $CDCl_3$) δ 8.12 (s, 1H), 7.43 (d, J = 7.2 Hz, 1H), 7.30-7.19 (m, 1H), 7.10 (t, J = 7.5 Hz, 1H), 6.84 (d, J = 1.2 Hz, 1H), 6.82-6.75 (m, 1H), 6.72 (dd, J = 8.4, 2.0 Hz, 1H), 4.74 (s, 1H), 3.74 (s, 3H), 2.55 (s, 3H), 2.51 (t, J = 8.4 Hz, 2H), 2.40 (t, J = 7.2 Hz, 2H), 1.88 – 1.57 (m, 2H). ^{13}C NMR (101 MHz, $CDCl_3$) δ 169.99, 155.29, 151.29, 142.30, 128.69, 128.64, 127.88, 124.37, 123.38, 118.82, 117.01, 114.17, 110.47, 109.98, 107.56, 55.96, 54.75, 36.86, 24.76, 19.77, 16.98. HRMS (ESI) calculated for $C_{21}H_{20}N_2O_3$ $[M+H]^+$: 349.1547; found: 349.1541.

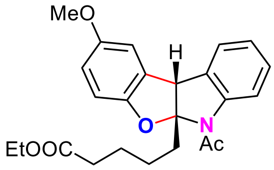

Ethyl 5-(6-acetyl-2-methoxy-6,10b-dihydro-5aH-benzofuro[2,3-b]indol-5a-yl)pentanoate (4d): colorless oil was obtained in 83% isolated yield. ^1H NMR (400 MHz, $CDCl_3$) δ 8.18 (s, 1H), 7.41 (d, J = 7.2 Hz, 1H), 7.22 (t, J = 7.6 Hz, 1H), 7.08 (t, J = 7.6 Hz, 1H), 6.84 (d, J = 2.4 Hz, 1H), 6.76 (d, J = 8.8 Hz, 1H), 6.70 (dd, J = 8.8, 2.4 Hz, 1H), 4.74 (s, 1H), 4.09 (q, J = 7.2 Hz, 2H), 3.73 (s, 3H), 2.54 (s, 3H), 2.47 – 2.29 (m, 2H), 2.27 (t, J = 7.6 Hz, 2H) 1.73-1.60 (m, 2H), 1.50 – 1.28 (m, 2H), 1.21 (t, J = 7.2 Hz, 3H). ^{13}C NMR (101 MHz, $CDCl_3$) δ 173.13, 170.22, 155.06, 151.55, 142.60, 129.06, 128.44, 128.25, 124.13, 123.19, 117.06, 113.93, 110.27, 109.99, 108.30, 60.29, 55.93, 54.65, 37.89, 33.77, 24.60, 22.73, 14.13. HRMS (ESI) calculated for $C_{24}H_{27}NO_5$ $[M+H]^+$: 410.1962; found: 410.1961.

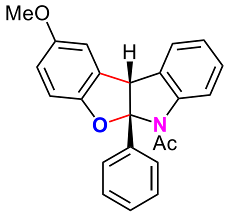

1-(2-Methoxy-5a-phenyl-5a,10b-dihydro-6H-benzofuro[2,3-b]indol-6-yl)ethan-1-one (4e): white solid was obtained in 87% isolated yield. ^{1}H NMR (400 MHz, $CDCl_3$) δ 8.39 (d, *J* = 8.4 Hz, 1H), 7.44 – 7.33 (m, 6H), 7.30 (t, *J* = 7.9 Hz, 1H), 7.11 (td, *J* = 7.6, 0.8 Hz, 1H), 6.93 (d, *J* = 8.4 Hz, 1H), 6.85 (d, *J* = 2.0 Hz, 1H), 6.76 (dd, *J* = 8.4, 2.4 Hz, 1H), 4.76 (s, 1H), 3.74 (s, 3H), 2.07 (s, 3H). ^{13}C NMR (101 MHz, $CDCl_3$) δ 171.45, 155.35, 151.82, 143.13, 141.68, 129.12, 128.80, 128.67, 128.57, 128.03, 124.47, 124.17, 123.50, 116.86, 114.04, 110.25, 110.08, 107.30, 60.56, 55.96, 25.04. HRMS (ESI) calculated for $C_{23}H_{19}NO_3$ $[M+H]^+$: 358.1438; found: 358.1439.

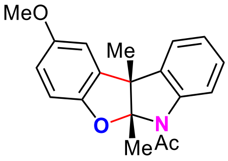

1-(2-Methoxy-5a,10b-dimethyl-5a,10b-dihydro-6H-benzofuro[2,3-b]indol-6-yl)ethan-1-one (4f): colorless oil was obtained in 77% isolated yield. ^{1}H NMR (400 MHz, $CDCl_3$) δ 8.15 (d, *J* = 4.4 Hz, 1H), 7.41 (d, *J* = 7.2 Hz, 1H), 7.20 (t, *J* = 7.6 Hz, 1H), 7.10 (t, *J* = 7.6 Hz, 1H), 6.77 (d, *J* = 8.0 Hz, 1H), 6.72-6.60 (m, 2H), 3.69 (s, 3H), 2.55 (s, 3H), 1.84 (s, 3H), 1.60 (s, 3H). ^{13}C NMR (101 MHz, $CDCl_3$) δ 170.10, 155.40, 149.77, 141.32, 133.92, 133.48, 128.29, 124.20, 121.76, 117.02, 113.55, 110.38, 109.40, 109.04, 57.76, 55.87, 24.49, 20.90, 20.41. HRMS (ESI) calculated for $C_{19}H_{19}NO_3$ $[M+H]^+$: 310.1438; found: 310.1437.

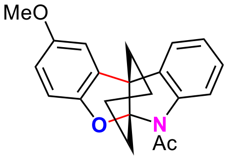

1-(2-Methoxy-6H-5a,10b-butanobenzofuro[2,3-b]indol-6-yl)ethan-1-one (4g):[1] brown oil was obtained in 83% isolated yield. ^{1}H NMR (400 MHz, $CDCl_3$) δ 8.17 (d, *J* = 6.8 Hz, 1H), 7.39 (dd, *J* = 7.2, 1.0 Hz, 1H), 7.21 (td, *J* = 8.0, 1.6 Hz, 1H), 7.10(td, *J* = 7.2, 1.2 Hz, 1H), 6.83 – 6.76 (m, 1H), 6.69 – 6.63 (m, 2H), 3.70 (s, 3H), 2.66 (td, *J* = 14, 4.4 Hz, 1H), 2.52 (s, 3H), 2.40 – 2.29 (m, 1H), 2.09 – 1.97 (m, 1H), 1.95 – 1.86 (m, 1H), 1.64 – 1.54 (m, 2H), 1.53 – 1.43 (m, 1H), 1.38 – 1.28 (m, 1H). ^{13}C NMR (101 MHz, $CDCl_3$) δ 170.25, 155.36, 150.62, 142.22, 133.91, 132.54, 128.28, 124.16, 121.70, 117.10, 113.32, 110.35, 109.28, 107.87, 57.44, 55.88, 31.28, 30.35, 24.50, 18.16, 17.87.

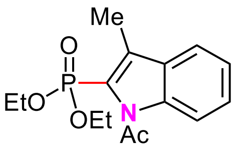

Diethyl (1-acetyl-3-methyl-1H-indol-2-yl)phosphonate (5a): colorless oil was obtained in 49%

isolated yield. ^1H NMR (400 MHz, $CDCl_3$) δ 7.89 (d, *J* = 8.4 Hz, 1H), 7.64 (d, *J* = 7.6 Hz, 1H), 7.45 (t, *J* = 7.8 Hz, 1H), 7.31 (t, *J* = 7.6 Hz, 1H), 4.42 – 4.08 (m, 4H), 2.81 (s, 3H), 2.59 (d, *J* = 2.4 Hz, 3H), 1.45 – 1.32 (t, *J* = 6.8 Hz, 6H). ^{13}C NMR (101 MHz, $CDCl_3$) δ 170.38, 137.44, 137.34, 133.10, 132.93, 130.15, 129.99, 127.60, 123.75, 123.00, 120.45, 114.37, 114.35, 62.68, 62.62, 27.13, 16.30, 16.23, 10.92. ^{31}P NMR (162 MHz, $CDCl_3$) δ 9.71. EI mass spectrometry: m/z calc. 309 [$C_{15}H_{20}NO_4P$], measured 309.

Supplementary References

1. Tomakinian T, Guillot R, Kouklovsky C, Vincent G. Direct oxidative coupling of *N*-acetyl indoles and phenols for the synthesis of benzofuroindolines related to phalarine. *Angew. Chem. Int. Ed.* **53**, 11881-11885 (2014).
2. Jiao L, Bach T. Palladium-catalyzed direct 2-alkylation of indoles by norbornene-mediated regioselective cascade C–H activation. *J. Am. Chem. Soc.* **133**, 12990-12993 (2011).
